# Multistimuli-Responsive Properties of Aggregated Isocyanide Cycloplatinated(II) Complexes

**DOI:** 10.1021/acs.inorgchem.2c01400

**Published:** 2022-07-01

**Authors:** Mónica Martínez-Junquera, Elena Lalinde, M. Teresa Moreno

**Affiliations:** Departamento de Química-Centro de Síntesis Química de La Rioja (CISQ), Universidad de La Rioja, 26006 Logroño, Spain

## Abstract

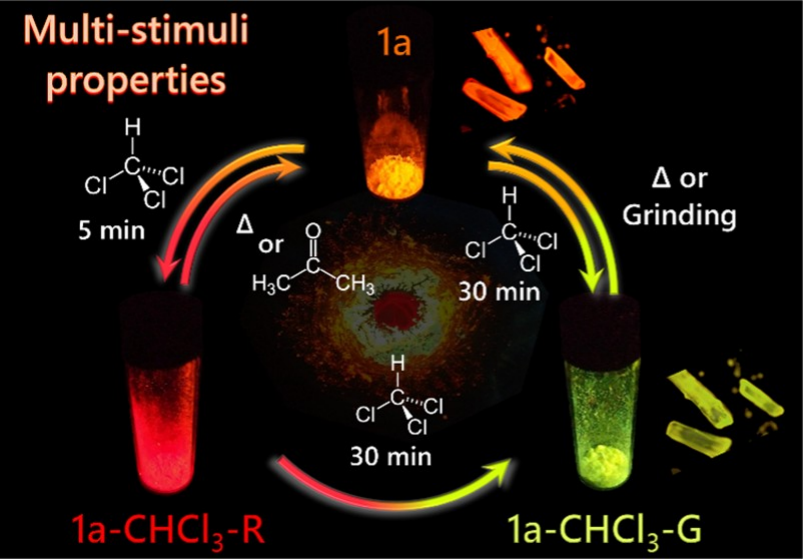

Here, we describe the neutral cyclometalated *tert-*butylisocyanide Pt^II^ complexes, [Pt(C^∧^N)Cl(CNBu*^t^*)] **1**, the double salts [Pt(C^∧^N)(CNBu*^t^*)_2_][Pt(C^∧^N)Cl_2_] **2**, and the cationic complexes [Pt(C^∧^N)(CNBu*^t^*)_2_]ClO_4_**3** [C∧N = difluorophenylpyridine (dfppy, **a**), 4-(2-pyridyl)benzaldehyde (ppy-CHO, **b**)]. A comparative study of the pseudopolymorphs **1a**, **1a·CHCl**_**3**_, **1b**, **1b·0.5Toluene**, **1b·0.5PhF**, and **3a·0.25CH**_**2**_**Cl**_**2**_ reveals strong aggregation through Pt···Pt and/or π···π stacking interactions to give a variety of distinctive one-dimensional (1D) infinite chains, which modulate the photoluminescent properties. This intermolecular long-range aggregate formation is the main origin of the photoluminescent behavior of **1a** and **1b** complexes, which exhibit highly sensitive and reversible responses to multiple external stimuli including different volatile organic compounds (VOCs), solvents, temperatures, and pressures, with distinct color and phosphorescent color switching from green to red. Furthermore, complex **1b** undergoes supramolecular self-assembly *via* Pt···Pt and/or π···π interactions into a polymer thin polystyrene (PS) film 10 wt % in response to toluene vapors, and **3a** exhibits vapochromic and vapoluminescent behavior. Theoretical simulations on the dimer, trimer, and tetramer models of **1a** and **1b** have been carried out to get insight into the photophysical properties in the aggregated solid state.

## Abstract 2

Cyclometalated isocyanide Pt^II^ complexes have shown strong aggregation through Pt···Pt and/or π···π stacking interactions, which modulate the photoluminescent properties and achieve switch color and emission color in response to VOCs, solvents, temperatures, and pressures.

## Introduction

Currently, there is a great deal of interest in producing chromic Pt^II^ complexes, with changes in the color or luminescence upon the application of external stimuli.^1^ Most of the studies focus on changes in luminescence using as external stimuli liquids,^2^ vapors,^3^ temperature,^3d,4^ or mechanical grinding^5^ of interest in memory, sensors, and optoelectronics.^6^ Some excellent reviews about these topics have been published.^7^ Many complexes only respond to a single external stimulus, with the systems that respond to multiple external stimuli being rare.^3d,4b,8^

The versatile chromic behavior of the Pt^II^ complexes is a consequence of their rigid square planar geometry, which enables noncovalent π···π stacking and intra- or intermolecular Pt···Pt interactions *via* open axial positions. These interactions endow supramolecular structures generating ^3^ππ excimeric and metal–metal-to-ligand charge-transfer (^3^MMLCT) transitions, respectively, remarkably red-shifted in comparison to the intraligand (^3^IL), ligand–ligand charge-transfer (^3^LLCT) or metal-to-ligand charge-transfer (^3^MCLT) transitions present in the monomer.^7a,7g,9^ The interplanar and metallophilic interactions, with Pt···Pt separations shorter than the sum of van der Waals radii, can allow the formation of dimers, trimers, oligomers, or one-dimensional (1D)-stacked structures,^3a,7g,10^ and some of these systems have been successfully utilized to develop white light, deep-red to near-infrared organic light-emitting diodes (OLEDs) or data storage devices.^11^ However, intermolecular interactions can also cause nonradiative deactivation.^12^ Considering that the metallophilic Pt···Pt interactions show a bond strength comparable to that of the hydrogen bonding,^13^ these relatively weak interactions can be affected by steric effects or other noncovalent intra-/intermolecular interactions such as solvation, hydrogen/halogen interactions, etc.^7b,14^ In consequence, noncovalent interactions play key roles in the assembly of platinum systems that respond to external stimuli and in the modulation of the photoluminescence properties of the resulting materials.^7a,15^

Among chromic Pt^II^ systems, a number of cyclometalated Pt^II^ complexes have been developed as a means to achieve bright, colorful luminescent materials.^1,2,7c,7e,7i,16^ On the other hand, isocyanides are versatile synthons for photoluminescent platinum complexes, which show stimuli response properties,^17^ and on some occasions, the luminescence can be modulated by noncovalent interactions.^18^ In this field, some chromic cycloplatinated complexes containing the strong field ligand isocyanides have been documented,^14a,19^ and it has been demonstrated that their photophysical properties can be modulated effectively by adjusting steric effects, the strength of the intermolecular interactions, or the medium. In this line, our group has reported two families of chloro-cyclometalated complexes, the benzoquinolinyl [Pt(bzq)Cl(CNR)],^14a^ in which isocyanide was varied, [R = *tert*-butylisocyanide (Bu*^t^*); 2,6-dimethylphenyl (Xyl); 2-naphthyl (2-Np)], and the xylylisocyanide compounds, [Pt(C^∧^N)Cl(CNXyl)], in which the cyclometalated backbone was modified [HC^∧^N = 2-phenylpyridine (ppy); 2,4-difluorophenylpyridine (dfppy); and phenylquinoline (pq)],^19a^ aiming to examine the role of the intermolecular π···π and short- or long-range Pt···Pt stacking interactions on their structural arrays and photophysical properties. The higher self-assembly behavior in the ground and excited state was found for the bzq/CNBu*^t^*, 2-Np and ppy-based complexes.

In this work, we report the preparation, characterization, and optical study of cyclometalated isocyanide Pt^II^ complexes, [Pt(C^∧^N)Cl(CNBu*^t^*)] **1**, the double salts [Pt(C^∧^N)(CNBu*^t^*)_2_][Pt(C^∧^N)Cl_2_] **2**, and the cationic complexes [Pt(C^∧^N)(CNBu*^t^*)_2_]ClO_4_**3** with difluorophenylpyridine (dfppy, **a**) or formyl-functionalized phenylpyridine (ppy-CHO, **b**) cyclometalated ligands. Among them, we discuss in depth the relationship between the structure–optical properties of complexes that display chromism upon the application of one or several stimuli. In particular, we present the differences in the crystal packing and luminescent properties of several pseudopolymorphs of **1a** and **1b** and the effect of solvents (vapochromism and solvatochromism), temperature (thermochromism), and mechanical force (mechanochromism), together with the vapochromism of **3a**.

## Synthesis and Characterization of Complexes

The synthesis of the cycloplatinated chloride–isocyanide complexes [Pt(C∧N)Cl(CNBu*^t^*)] (**1**) and bis-isocyanide [Pt(C^∧^N)(CNBu*^t^*)_2_]ClO_4_ (**3**) complexes was carried out using the corresponding complexes containing HC∧N as an auxiliary ligand as precursors, [Pt(C^∧^N)(HC^∧^N)Cl] (C^∧^N = dfppy **Ia**,^20^ ppy-CHO **Ib**). Precursor **Ib** was prepared in this work following a conventional protocol, which involves the reaction of K_2_PtCl_4_ with an excess of HC^∧^N in an ethoxyethanol/water mixture at 80 °C.

**Scheme 1:**
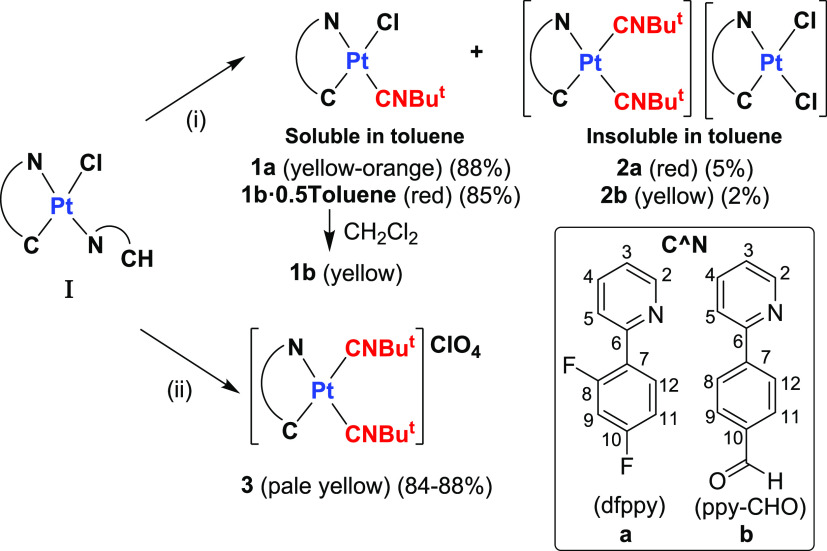
Synthesis of **1**–**3** (**a**, **b**), (i) 1 equiv of CNBu*^t^*, CH_2_Cl_2_; Dryness and Treatment with Toluene, (ii) Excess of KClO_4_ and 2 equiv of CNBu*^t^*, Acetone/CH_2_Cl_2_ (1/1), and 298 K

The reaction of the corresponding precursor **Ia** or **Ib** with 1 equiv of CNBu*^t^* in CH_2_Cl_2_ at room temperature and further treatment of the dry residue with toluene gave a minority solid, insoluble in toluene, and a major product soluble in toluene (Scheme 1i). The main product, identified as [Pt(C^∧^N)Cl(CNBu*^t^*)], was obtained from the toluene solution in high yields (85–88%) as a yellow-orange (C^∧^N = dfppy **1a**) or a red solvated solid (C^∧^N = ppy-CHO **1b·0.5Toluene**). Interestingly, **1b·0.5Toluene** keeps its color in solid for more than 1 month, finally changing to a yellow solid on standing, suggesting that a structural transformation takes place. Indeed, it is also isolated as unsolvated yellow solid (**1b**) by dissolution in CH_2_Cl_2_ and evaporation to dryness. These complexes **1** are rather soluble in common organic solvents. Spectroscopic analysis and X-ray diffraction (XRD) of crystals of **1a** and **1b** from different solvents (see below) reveal that complexes **1** display the stereochemistry shown in Scheme 1 with CNBu*^t^* occupying the trans position to the nitrogen of the C^∧^N ligand, similarly to other chloride–isocyanides published^14a,19a,21^ and consistent with the lower trans influence of Cl^–^ compared to the CNBu*^t^* ligand. They exhibit one ν(Pt–Cl) absorption at 289 cm^–1^, consistent with a terminal Pt–Cl bond trans to C, and one absorption at 2207 **1a** and 2205 cm^–1^**1b**, assignable to ν(C≡N) of terminal CNBu*^t^*, which is shifted to higher frequencies with respect to the free ligand (2125 cm^–1^). Spectroscopically, the microcrystals obtained from different solvents exhibit almost identical ν(C≡N) bands to the pristine solids (2207–2211 **1a**, 2202–2205 cm^–1^**1b**). Their electrospray ionization (ESI)(+)-mass spectrometry (MS) spectra show the peak corresponding to the loss of chloride ([M – Cl]^+^) and that corresponding to the dimer [2M – Cl]^+^, suggesting a strong tendency to form aggregates. ^1^H and ^13^C{^1^H} NMR spectra of complexes **1** display the expected signals for C^∧^N and CNBu*^t^* in a 1:1 intensity ratio, which were assigned on the basis of ^1^H–^1^H and ^13^C–^1^H correlations (see the Experimental Section and Figures S1 and S2). The minority solids were proposed as the double salts [Pt(C^∧^N)(CNBu*^t^*)_2_][Pt(C^∧^N)Cl_2_] (red solid, C^∧^N = dfppy, **2a**; yellow, C^∧^N = ppy-CHO **2b**) in agreement with their matrix-assisted laser desorption ionization time-of-flight (MALDI-TOF) spectra, which show peaks due to the corresponding anion [Pt(C^∧^N)Cl_2_]^−^ and cation [Pt(C^∧^N)(CNBu*^t^*)_2_]^+^ operating in a negative and positive mode, respectively (Figures S3 and S4), and two characteristic ν(C≡N) absorptions due to the two terminal CNBu*^t^* ligands. To corroborate this proposal, red complex **2a** was obtained alternatively by a reaction of (NBu_4_)[Pt(dffpy)Cl_2_] (generated *in situ* from [Pt(dfppy)(μ-Cl)]_2_ with 2 equiv of NBu_4_Cl in refluxing MeOH) and complex **3a**, [Pt(dffpy)(CNBu*^t^*)_2_]ClO_4_. Its insolubility in common organic solvents prevents the characterization by NMR spectroscopy and X-ray spectroscopy. **2a** does not show solid-state evolution by thermal treatment in an oven (100 °C) for 24 h into the corresponding neutral complex [Pt(dfppy)Cl(CNBu*^t^*)], a common feature in double salts.^14a,22^ A similar reaction between (NBu_4_)[Pt(ppy-CHO)Cl_2_] and **3b** also evolves with the formation of the expected salt **2b** but mixed with **1b** due to a relatively fast rearrangement of the salt. This type of behavior is not unusual. Indeed, the salt [Pt(bzq)(CN-2-Np)_2_][Pt(bzq)Cl_2_] was also formed as a subproduct together with [Pt(bzq)Cl(CN-2-Np)] by reacting [Pt(bzq)(μ-Cl)]_2_ and CN-2-Np in a 1:2 molar ratio.^14a^

Cationic complexes [Pt(C^∧^N)(CNBu*^t^*)_2_]ClO_4_ (C^∧^N = dfppy **3a**, ppy-CHO **3b**) were prepared by treatment of a suspension of the corresponding [Pt(C^∧^N)(HC^∧^N)Cl] (**Ia**, **Ib**) precursor with an excess of KClO_4_ and the subsequent addition of 2 equiv of CNBu*^t^* in a mixture of acetone/CH_2_Cl_2_ (1/1) (Scheme 1ii). These complexes were isolated as pale-yellow air-stable solids in good yields (84–88%). They exhibit two terminal ν(C≡N) absorptions at 2236, 2215 **3a**, 2248, and 2223 cm^–1^**3b**, shifted to higher energies than in **1**, and peaks at 1085 and 622–624 cm^–1^, assigned to the ionic ClO_4_^–^. The ESI(+) mass spectra show the corresponding [Pt(C^∧^N)(CNBu*^t^*)_2_]^+^ peaks and the NMR spectra (^1^H and ^13^C{^1^H}) show the expected signals of C^∧^N/CNBu*^t^* in a 1:2 intensity ratio. It is worth noting that in **3**, the most deshielded H^2^ proton appears high-field shifted in relation to complexes **1** (δ H^2^ 8.90 **3a***vs* 9.47 **1a**; 8.78 **3b***vs* 9.80 **1b**) (Figures S5 and S6), as a consequence of the substitution of Cl^–^ by CNBu*^t^*.

## Crystallographic Studies

**Figure 1:**
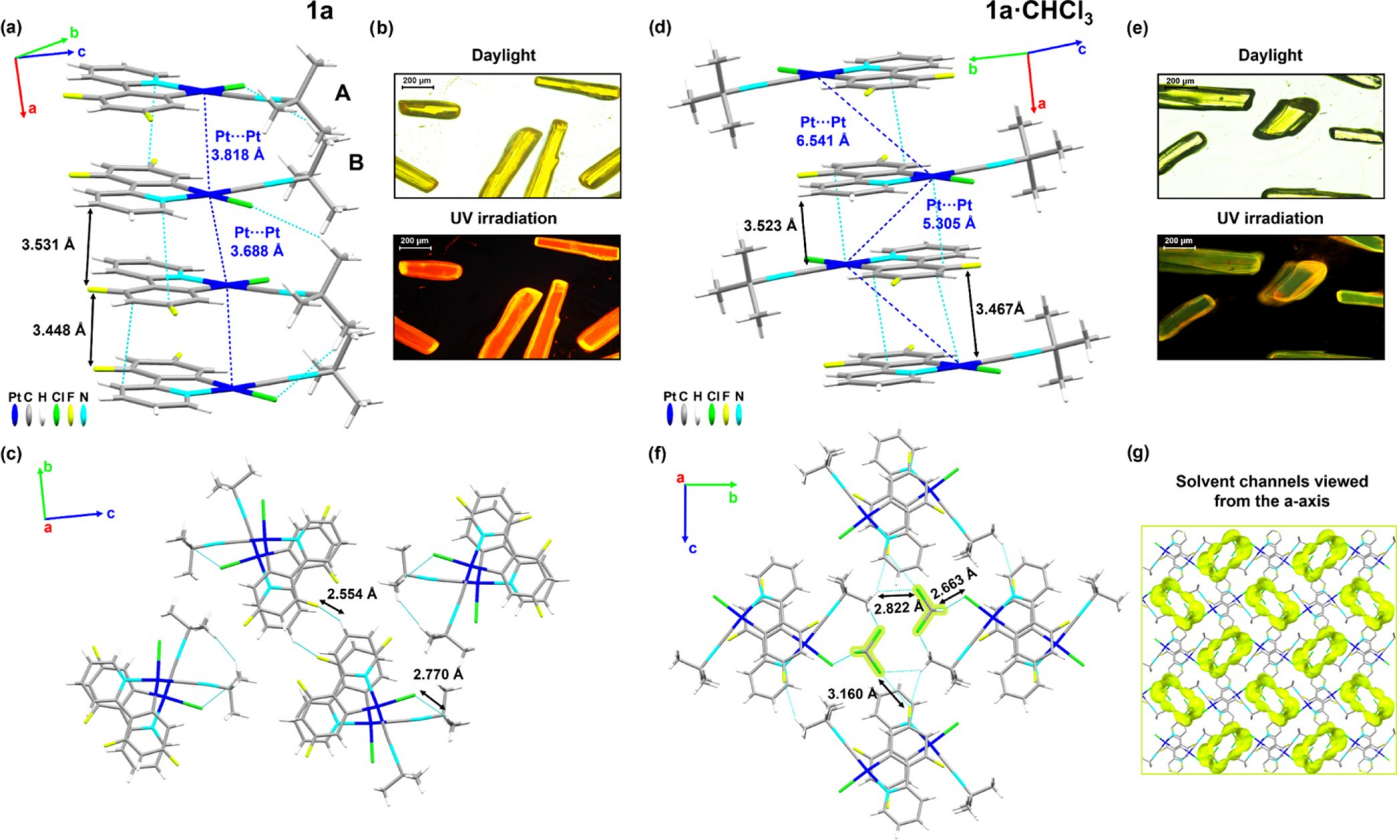
(a) Single infinite crystal stacking along the *a-*axis of molecules AB showing the π···π interplanar and Pt···Pt distances. Hydrogen atoms are omitted for clarity. (b) Pictures of crystals of **1a** under daylight or UV irradiation. (c) Top view from the *a-*axis of four stackings showing also C–H···F contacts between columns. (d) Packing structure of **1a·CHCl**_**3**_ along the *a-*axis showing the π···π interplanar and Pt···Pt distances. (e) Pictures of crystals of **1a·CHCl**_**3**_ under daylight or UV irradiation. (f) Axial visualization of solvent interactions along the stacking direction. (g) View of the packing structure with the solvent channels marked in yellow along the *a-*axis. This picture was illustrated using the Mercury computer program.^24^

**Figure 2:**
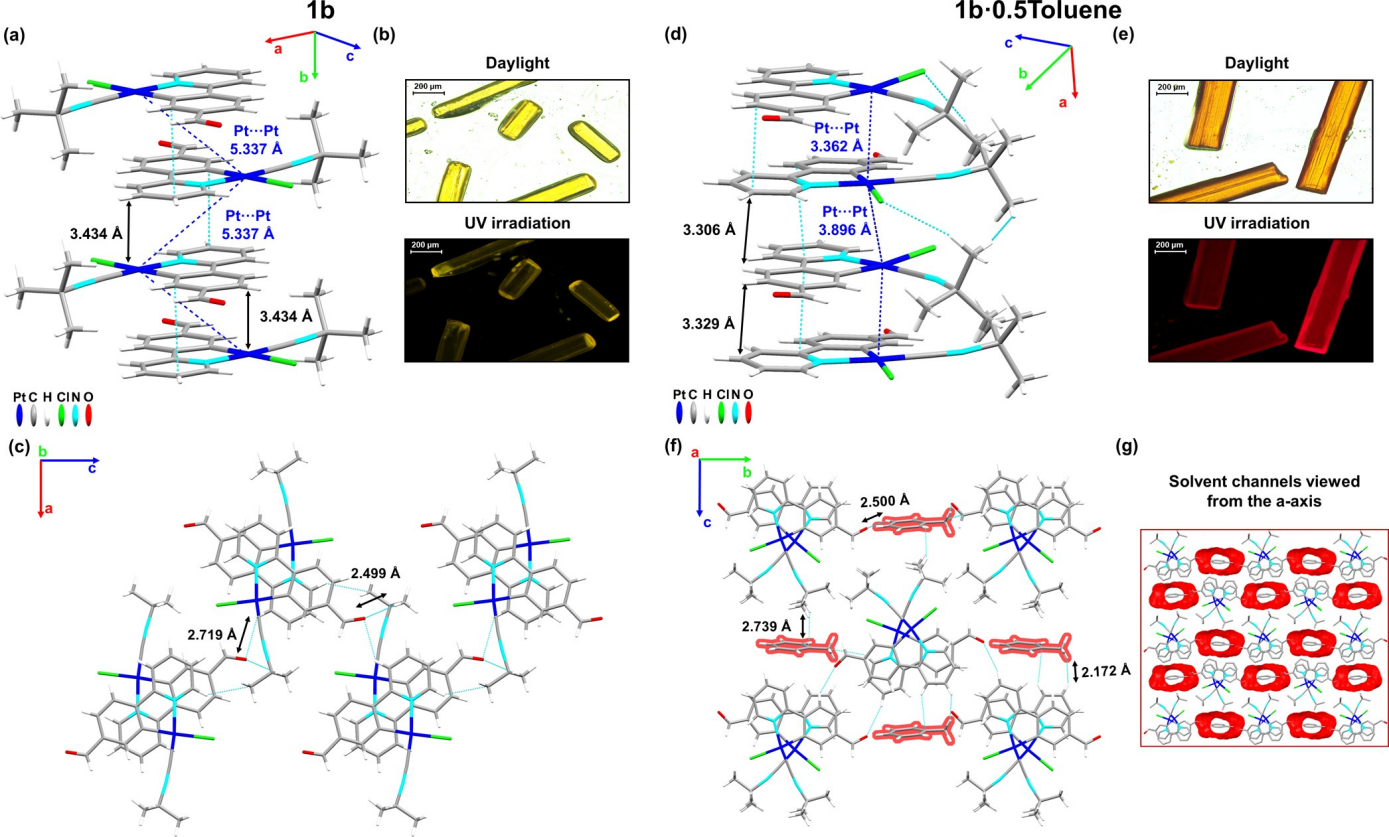
(a) Single infinite crystal packing of **1b** along the *a-*axis showing the π···π interplanar and Pt···Pt distances. (b) Pictures of crystals of **1b** under daylight or UV irradiation. (c) Top view from the *b-*axis of four columns, showing also H_(ppy–CHO)_···O_(ppy–CHO)_ interactions. (d) Columnar stacking along the *a-*axis of **1b·0.5Toluene** showing the π···π interplanar and Pt···Pt distances. (e) Pictures of crystals of **1b·0.5Toluene** under daylight or UV irradiation. (f) Top view from the *a-*axis of five stackings showing the toluene solvent localization and secondary contacts. (g) View of the packing structure with the solvent channels marked in red along the *a-*axis.

For **1a**, crystals suitable for X-ray diffraction were obtained by slow diffusion of *n*-hexane into a CH_2_Cl_2_ solution (**1a**) or by evaporation from a concentrated CHCl_3_ solution (**1a·CHCl**_**3**_), and for **1b**, by slow diffusion at 298 K of *n*-hexane into tetrahydrofuran (THF), toluene, or fluorobenzene solutions, respectively (**1b**, **1b·0.5Toluene**, **1b·0.5PhF**), and they were subjected to single-crystal X-ray analysis. The basic crystallographic data and selected bond distances and angles are summarized in Tables 1 and S1–S3. Their structural analyses reveal the expected distorted square planar geometry with the isocyanide ligand in the trans position with respect to the nitrogen of the cyclometalated groups (Figures 1 and 2). All distances and angles are comparable to those observed in related complexes.^14a,19a,21^

**Table 1:** Color, Color Emission, and Selected Distances (Å) and Angles (deg) of Crystals **1a**, **1a·CHCl**_**3**_, **1b**, **1b·0.5Toluene**, and **1b·0.5PhF**

	**1a**^a^	**1a·CHCl_3_**	**1b**	**1b·0.5Toluene**^a^	**1b·0.5PhF**^a^
color	yellow	pale-yellow	yellow	orange-red	orange-red
emission	orange	green-yellow	yellow	red	red
Pt(1)–Cl(1) (Å)	2.385(2)	2.399(12)	2.3952(8)	2.4054(9)	2.4001(9)
Pt(1)–N(1) (Å)	2.054(5)	2.054(3)	2.057(3)	2.057(3)	2.061(3)
Pt(1)–C_ort_ (Å)	1.986(3)	1.986(4)	1.992(3)	1.992(3)	1.988(4)
Pt(1)–C_CNBu_*_^t^_* (Å)	1.921(7)	1.898(4)	1.894(3)	1.913(4)	1.908(4)
Pt···Pt (Å)	3.818_(dimer)_/3.688	5.305/6.541	5.337	3.362_(dimer)_/3.896	3.370_(dimer)_/3.864
*d*_interplanar_ (Å)^b^	3.448_(dimer)_/3.531	3.467/3.523	3.434	3.329_(dimer)_/3.306	3.298_(dimer)_/3.331
Pt···Pt···Pt (deg)	∼145	73.33	79.70	∼147	∼146
C_α_–Pt–Pt–C_α_ (deg)	75.93/72.84	180.0	180.0	64.79/58.51	63.80/57.78

a Pair of molecules with similar distances and angles were found in the crystals of **1a**, **1b·0.5Toluene**, and **1b·0.5 PhF**. Data are given for molecule A.

b Shortest interplanar distance.

Yellow needles of **1a** show orange emission similar to **1a** powder, whereas the pale-yellow crystals of **1a·CHCl**_**3**_ display a green-yellow emission due to their different molecular stacking (Figure 1). **1a** presents two nearly identical molecules (A and B) in the asymmetric unit (data given for A in Table 1) that form head-to-head slightly twisted parallel dimers (AB), which stack along the *a-*axis (Figure 1a), with alternating C_α_–Pt–Pt–C_α(CNBu*^t^*)_ angles of 75.93/72.84°, interplanar dfppy π···π interactions of 3.448 (dimer)/3.531 Å and Pt···Pt distances of 3.818 (dimer)/3.688 Å, close to the van der Waals limit, which is around 3.5 Å.^23^ The Pt atoms in the columns are rather aligned with a Pt–Pt–Pt angle of ∼145°. Inside the columns, there are also secondary C–H_(Bu*^t^*)_···Cl interactions (2.770 Å) and the columns interact through C–H···F contacts (2.554 Å) (Figure 1c).

The **1a·CHCl**_**3**_ crystals also show a staggered columnar packing. The molecules stack along the *a*-axis in a head-to-tail manner with an antiparallel arrangement, which minimizes the repulsion between the *tert*-butyl moieties (C_α_–Pt–Pt–C_α_ angle of 180°, Figure 1f), and alternating π···π interactions, similar to those found in **1a** (3.467 and 3.523 Å, Figure 1d and Table 1). The Pt–Pt distances are longer than in **1a** (5.305 and 6.541 Å) with a zigzag Pt–Pt–Pt angle of 73.33°. Interestingly, the molecules of the solvent are occluded in continuous channels along the *a*-axis, with two rows of CHCl_3_ per cavity interacting with the complex [Cl···Cl/H_(solvent)_ 2.663 Å, F···Cl_(solvent)_ 3.160 Å, and H_(Bu*^t^*)_···Cl_(solvent)_ 2.822 Å] (Figures 1f,g and S7). The channels have an aperture size of 11.1 Å × 10.3 Å, and the total void volume occupies 26.3% of the unit cell. The green-yellow emission of these crystals changes to orange on standing in the air (4 h), suggesting that the CHCl_3_ molecules are lost, reversing to **1a**. The shorter Pt–Pt distances in **1a** could account for the lower-energy emissions of the crystals of **1a** in relation to **1a·CHCl**_**3**_. The vapochromic behavior of the powder **1a** (see below) could be related to the occurrence of a structural transformation from the initial framework of **1a**, in which the molecules present a head-to-head disposition to the porous channel structure of **1a·CHCl**_**3**_, with the molecules adopting a head-to-tail arrangement. The solvent channels appear to be more accessible to absorb vapors of solvent molecules, thereby resulting in a rapid vapochromic response (see below).

Yellow needles of **1b** display a staggered columnar packing, which resembles **1a·CHCl**_**3**_ (Figure 2), with a regular antiparallel head-to-tail arrangement of neighboring molecules with π···π (ppy-CHO) interactions of 3.434 Å and a long Pt···Pt distance (5.337 Å), indicative of negligible metallophilic interactions. The Pt–Pt–Pt and torsional C_α_–Pt–Pt–C_α_ angles are 79.70 and 180.0°, respectively (Figure 2a,c).

The red needles crystals of **1b·0.5Toluene** and **1b·0.5PhF** display very similar crystallographic data and structural arrangement, with small variations that fit within the experimental error (Figures 2 and S8). Therefore, only the structure of **1b·0.5Toluene** will be discussed. In **1b·0.5Toluene**, the two nearly identical molecules (A and B) found in the asymmetric unit form a head-to-head dimer with a short Pt···Pt distance of 3.362 Å and a π···π (ppy-CHO) interplanar distance of 3.329 Å (Figure 2d,f). The short distances^23^ imply a strong Pt–Pt interaction within the dimeric unit, which could account for the lower-energy emissions of crystals **1b·0.5Toluene** at 298 K, intensified by cooling. The dimers stack along the *a-*axis to form a columnar structure with a C_α_–Pt–Pt–C_α_ torsion angle of 64.79/58.51° and a slight zigzag Pt–Pt–Pt arrangement (angle ∼147°), thus increasing the Pt···Pt distance (3.896 Å) between dimers. These columns are mainly supported by interplanar ppy-CHO π···π (3.306 Å) and secondary Cl···H_(Bu*^t^*)_ (2.893 Å) interactions. A notable structural feature is the presence of a channel with an approximately 6 Å pore diameter along the *a-*axis, running parallel with the Pt···Pt stacks, partly occupied by toluene molecules in this red crystalline form, supported by weak contacts such as O_(ppy-CHO)_···H_Ph/Me(Tol)_ (2.500 Å), H_(ppy-CHO/Bu)_···C_Me(Tol)_ (2.739 Å), and H_(ppy-CHO)_···H_Me(Tol)_ (2.172 Å) (Figures 2g and S9). The solvent-accessible volume occupies 22.7% of the total volume of the unit cell for **1b·0.5Toluene** and 18.1% for **1b·0.5PhF**. Different solvents were studied (THF, xylene, acetone, CHCl_3_, and MeCN), and only red crystals were obtained from toluene and fluorobenzene. This fact suggests that the formation of channels seems to be driven by the required solvent space and the interactions between solvent molecules and the platinum moieties. These structures are stable with time, as solvent molecules are “trapped”, as evidenced by the fact that the red crystal lasted more than 1 month to lose the solvent and its color into the air. Structural analysis of **1b·0.5Toluene** and **1b·0.5PhF** provides insight into the solvent exchange mechanism, enabling rationalization of the vapochromic response that will be detailed in the corresponding section (see below).

**Figure 3:**
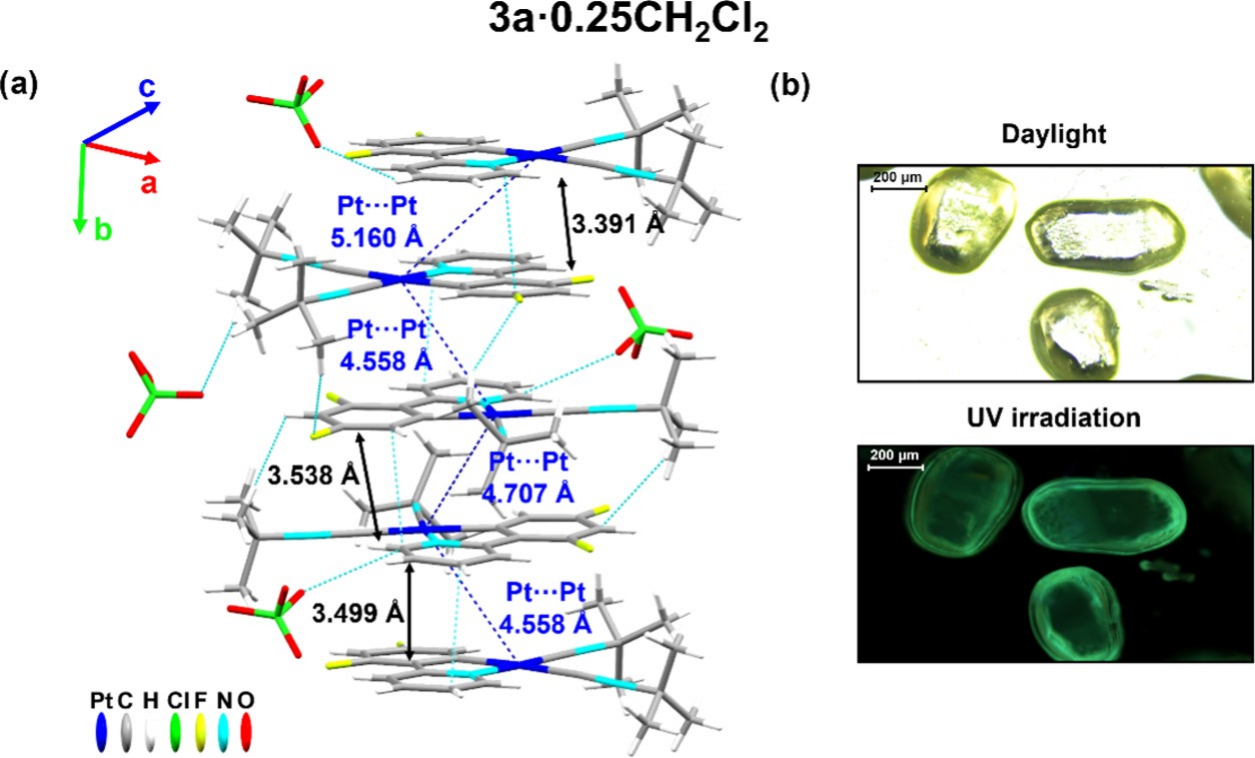
(a) Single infinite crystal packing of **3a·0.25CH**_**2**_**Cl**_**2**_ along the *b-*axis showing the π···π interplanar and Pt···Pt distances. (b) Pictures of crystals of **3a·0.25CH**_**2**_**Cl**_**2**_ under daylight or UV irradiation.

Microcrystals of **3a·0.25CH**_**2**_**Cl**_**2**_ and pale-yellow blocks of **3b** were obtained by slow diffusion of *n*-hexane into a solution of the corresponding compound in CH_2_Cl_2_ at 298 K (Figures 3 and S10 and Table S4). For **3b**, the quality of the data collection was not good enough, and only the connectivity and the packing were established (Figure S11). Crystals of **3a·0.25CH**_**2**_**Cl**_**2**_ contain four nearly identical molecules in the asymmetric unit, and only selected parameters for molecule A are given in Table S5. The cation exhibits the expected distorted square planar geometry formed by the dfppy and two *tert-*butylisocyanide ligands. In the crystal lattice, the cations form columnar structures along the *a*- and *b*-axis *via* partial π-stacking between two dfppy units from neighboring molecules, which have an antiparallel arrangement with short π···π interactions (3.391 Å) and a long Pt···Pt separation (5.160 Å). As shown in the Supporting Information (Figure S10), the dimers packed with other twisted units through a longer π···π (3.499, 3.538 Å) and a shorter Pt···Pt distance (4.558, 4.707 Å) and also show secondary C–H_(Bu*^t^*)_···F_(dfppy)_ (2.472 Å) and H(_dfppy/Bu*^t^*_)···O_(ClO4)_ (2.494–2.525 Å) contacts.

## Photophysical Properties and Theoretical Calculations

### Absorption Spectra in Solution and Density Functional Theory (DFT) Calculations

**Figure 4:**
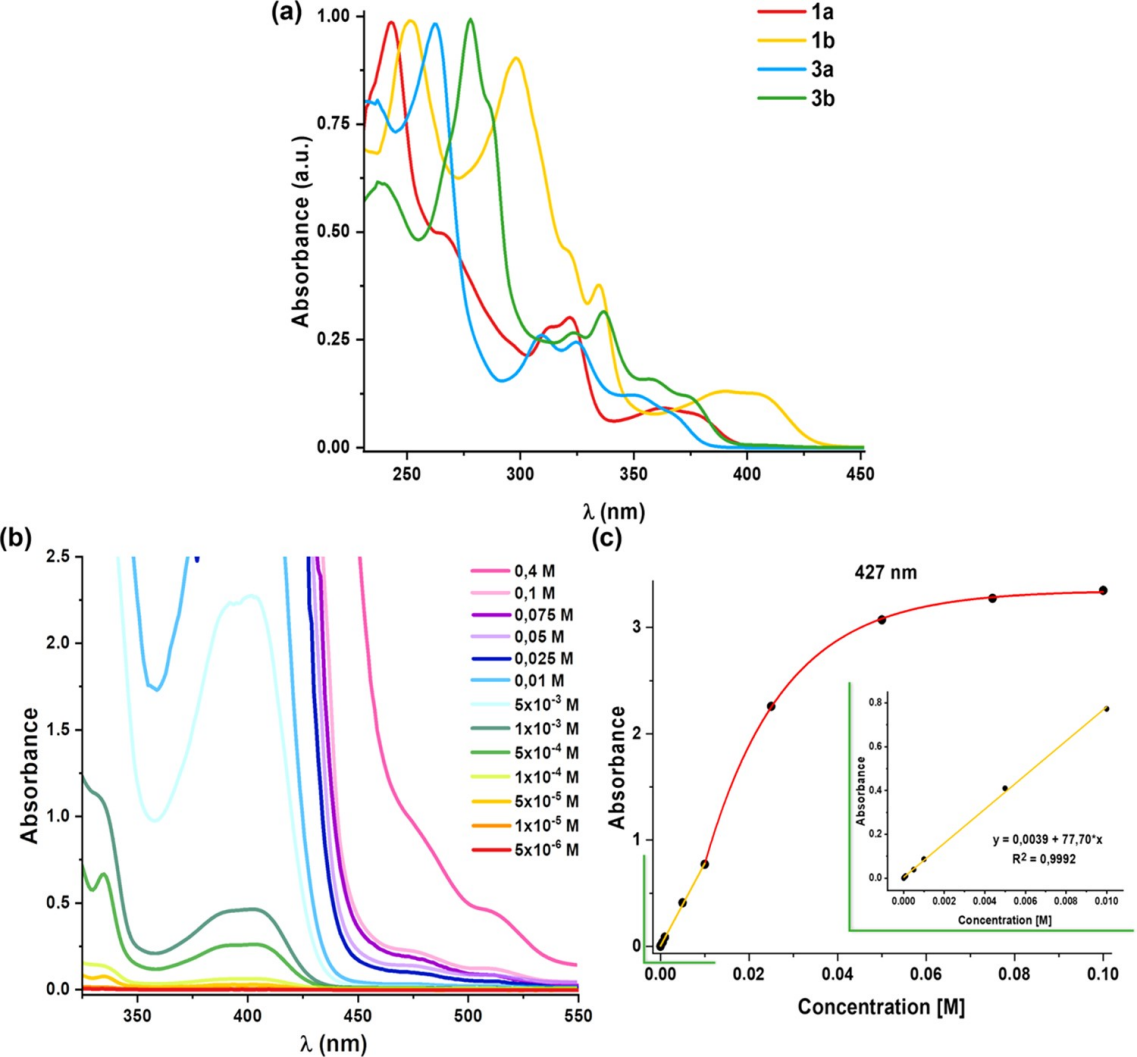
(a) Normalized absorption spectra of complexes **1a**, **1b**, **3a**, and **3b** in CH_2_Cl_2_ at 298 K. (b) Low-energy region of the UV–vis absorption spectra of **1b** in CH_2_Cl_2_ at different concentrations. (c) Representation of the absorbance at the 427 nm band *vs* concentration. Green box: the expansion of the low concentration range (<0.01 M).

The absorption spectra of the complexes **1a**, **1b**, **3a**, and **3b** were recorded in a CH_2_Cl_2_ solution (5 × 10^–5^ M, Figure 4), and also in THF for compounds **1** (Figure S12), and the data are listed in Table S6. All complexes display intense high-energy absorption bands till ∼340 nm, assigned to a mixed intraligand ^1^IL(π–π*), located on the metalated C^∧^N ligand and charge-transfer (^1^MLCT/^1^L′LCT/^1^LL′CT) transitions. They also show a characteristic low-energy (LE) broad feature, red-shifted for the ppy-CHO compounds (**b**) in relation to that of dffpy (**a**) (389, 408 **1b***vs* 362, 379 **1a**; 360, 375 **3b***vs* 351, 368 nm **3a**) and for the neutral Cl/CNBu*^t^* (**1**) in relation to the cationic (CNBu*^t^*)_2_ (**3**), as expected on the basis of the lower energy of the corresponding lowest unoccupied molecular orbital (LUMO) and the highest occupied molecular orbital (HOMO), respectively (ppy-CHO *vs* dfpy on the LUMOs and cationic *vs* neutral on the HOMOs). The absorption spectra in CH_2_Cl_2_ and THF display similar patterns, with a slight negative solvatochromism for the LE band with a red shift on decreasing the polarity of the solvent (362, 379 **1a**, 389, 408 **1b** nm in CH_2_Cl_2_; 382 **1a**, 392, 412 nm **1b** THF), evidencing a certain charge-transfer (CT) nature (Figure S12).

**Figure 5:**
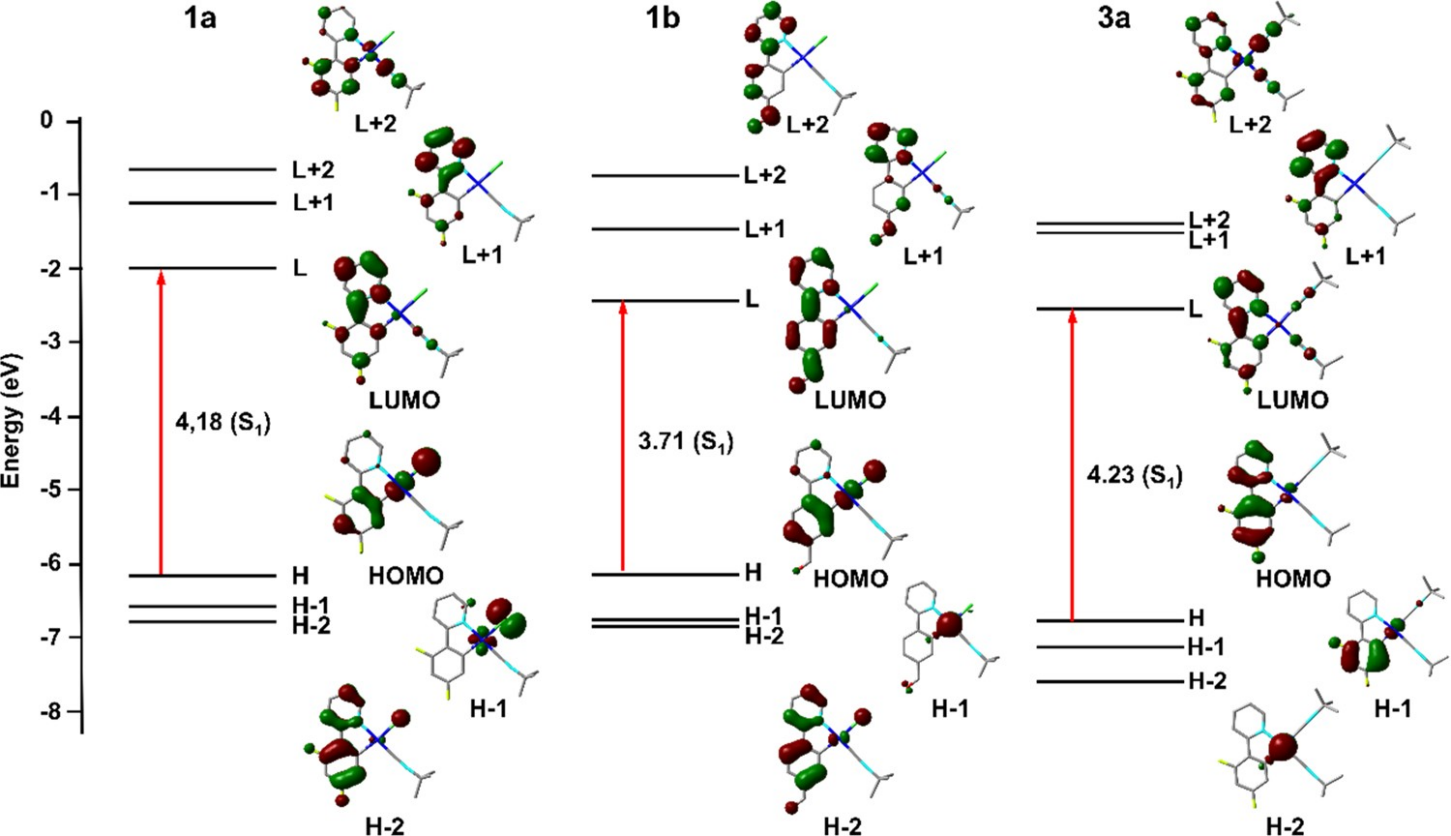
Schematic representation of selected frontier orbitals and excitations of **1a**, **1b**, and **3a**.

DFT/time-dependent (TD)-DFT calculations were carried out for monomeric species of **1a**, **1b**, and **3a** in CH_2_Cl_2_ (Tables S7–S9 and Figures S13–S16). In complexes **1**, the low-energy feature is mainly assigned to a ^1^IL/^1^MLCT admixture with some ^1^XLCT (X = Cl) character, whereas bis-isocyanide complex **3a** has an important ^1^IL character, with some ligand-to-ligand (^1^LL′CT, C^∧^N → C≡N) and ligand-to-metal ^1^LMCT character. The red shift observed for the ppy-CHO complexes (**b**) in relation to the dfppy (**a**) can be related to the stabilization of the LUMO induced by the presence of the aldehyde moiety (−1.98 **1a***vs* −2.43 eV **1b**), whereas the HOMO remains similar (−6.16 **1a***vs* −6.14 eV **1b**), which reduces the HOMO–LUMO gap. As is shown in Figure 5, the substitution of the chloride by other isocyanide ligand displays a blue shift in the LE absorption maxima (362, 379 **1a***vs* 351, 368 nm **3a**), which can be ascribed to the major C^∧^N contribution in **3a** (94%) with respect to **1a** (55%) in the HOMO and to the π-acidity of the isocyanide that stabilizes the HOMO, leading to a larger HOMO–LUMO gap.

A concentration dependence study in CH_2_Cl_2_ was carried out for **1a** and **1b**. In both complexes (Figure 4b,c for **1b**, and Figure S17 for **1a**), the lowest absorption band follows Beer’s law in the concentration range from 5 × 10^–6^ to 5 × 10^–3^ M, suggesting that no obvious ground-state aggregation occurs within these concentrations range. However, at higher concentrations (>5 × 10^–3^ M), a clear deviation from Beer’s law is observed for **1b** (monitored at 427 nm). Weaker absorptions are also discernible at lower energy (∼475 and 510 nm), indicating the formation of aggregates. This agrees with color and emission changes from pale-yellow to dark orange on increasing the concentration from 5 ×10^–3^ to 0.4 M. This aggregation is also reflected in the clear upfield shift of all aromatic proton signals upon increasing the concentration (Figure S18). However, complex **1a** exhibited lower solubility at concentrations >5 × 10^–3^ M, and the study is less conclusive.

### Emission Spectra in the Film, Solution, and TD-DFT Calculations

Photophysical properties of complexes **1a**, **1b**, **3a**, and **3b** were studied in doped polystyrene (PS) films (1–10 wt %) and CH_2_Cl_2_ and THF solutions (298, 77 K) and in the solid state; the relevant data are listed in Tables S10 and 2. Calculations on the lowest-lying (S_0_–T_1_, SI) and spin density distribution of the triplet excited states (T_1_) based on their corresponding optimized S_0_ and T_1_ geometries of the monomers are detailed in the SI.

**Table 2:** Photophysical Data for **1**–**3** (**a**, **b**) in the Solid State at 298 and 77 K

298 K				77 K	
compound	λ_em_/nm (λ_ex_/nm)	τ/μs	ϕ	λ_em_/nm (λ_ex_/nm)	τ/μs
**1a**	625 (420)	1.1 (63%), 0.5 (37%)	0.51	650 (420)	7.5
**1a-CHCl**_**3**_**-R**	662 (550)	0.24 (80%), 1.05 (20%)	0.21	660, 720_max_ (470)	9.5 (660)
				730 (580)	3.7 (717)
					4.2 (730)
**1a-CHCl**_**3**_**-G**	474_sh_, 534^a^ (420)	0.26 (51%), 1.68 (49%) (474); 22.7 (534)	0.11	474^a^ (440)	12.4
**1a-CHCl**_**3**_**-G-ground**	632 (460)	0.31 (35%), 1.35 (65%)	0.38	677 (468)	5.5
**1a(THF)**	627 (470)	0.3 (31%), 1.0 (69%)	0.43	654 (470)	8.9
**1a(MeOH)**	650 (525)	0.3 (70%), 0.9 (30%)	0.14	722 (530)	3.9
**1a(EtOH)**	651 (550)	0.4 (50%), 1.3 (50%)	0.26	718, 748_sh_ (500)	4.3 (718)
					2.5 (748)
**1a(CHCl**_**3**_**)**	660 (570)	0.3 (67%), 0.7 (33%)	0.22	724 (570)	3.2
**1a(CH**_**2**_**Cl**_**2**_**)**	689 (570)	0.2 (54%), 0.9 (46%)	0.21	739 (570)	2.3
**1b**	545^a^ (420)	0.3 (53%), 2.8 (48%)	0.06	550 (420)	22.6
**1b·0.5Toluene**	658	<1%	0.20	550_sh_, 596, 706 (420)	14.8 (550)
				706 (550)	12.6 (596)
					9.8 (706)
**1b-ground**	550^a^ (420)	0.32 (64%), 1.96 (36%)	0.03 (420)	557, 598, 660 (420)	17.2 (557)
	660 (500)	(585)	0.05(500)		10.2 (660)
**2a**	640 (550)	0.22	0.05	717 (550)	2.1
**2b**				650 (515)	2.0
**3a**	474,^a^ 595_max_ (385)	1.4 (595)	0.57	478^a^, 585_max_ (385)	40.7 (478)
				478^a^, 617_max_ (420)	8.9 (617)
**3a·0.25CH**_**2**_**Cl**_**2**_	490, 509_max_ (420)	24.8 (509)	0.15	488 (420)	47.4 (488)
**3a-CHCl**_**3**_	627 (430)	1.09	0.26	482^a^, 624_max_, 638_sh_, 721_sh_ (430)	32.5 (482)
					10.8 (624)
				650 (475)	9.3 (650)
				725 (590)	1.4 (700)
**3a-Acetone**	612 (430)	0.4	0.15	480^a^, 514, 613_max_, 645_sh_, 704_sh_ (430)	67.3 (476)
				640, 659, 696_sh_ (475)	10.7 (613)
				630, 690_max_ (550)	9.5 (650)
					1.5 (690)
**3a-THF**	623 (430)	0.6	0.16	476, 510, 616_max_, 705_sh_ (430)	54.6 (476)
					12.5 (616)
				645 (470)	6.8 (645)
				630, 700 (540)	2.4 (700)
**3b**	530 (440)	0.1 (58%), 0.8 (43%)	0.10	560, 628 (480)	15.8

a ν_0→0_ transition of the structured emission.

**Figure 6:**
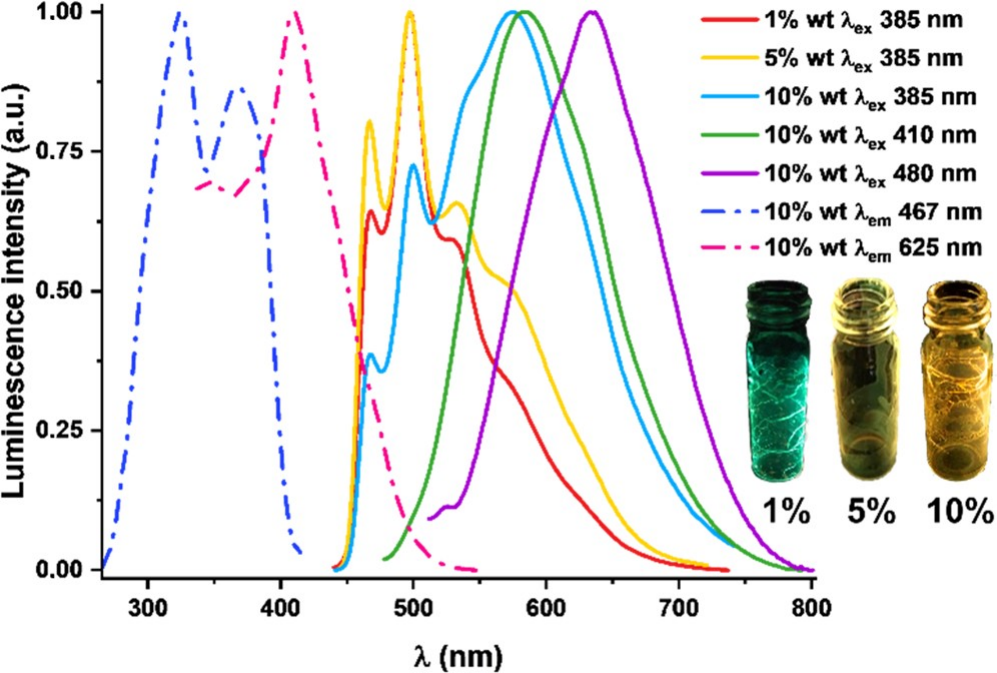
Normalized excitation (dashed line) and emission (solid line) spectra in PS at 1–10 wt % at 298 K in air of **1a**.

Both chloride–isocyanide derivatives **1a** and **1b** display in a diluted PS film (1 wt %) structured phosphorescence bands (λ_em_ = 467 **1a**, 524 nm **1b**, Figures 6 and S19), attributed to monomer emission (^3^IL/^3^MLCT) involving the cyclometalated ligand and the Pt atom, with a predominant ^3^IL character (see calculations below). At higher concentrations, only **1a** forms aggregates. Thus, at a doped concentration of 10 wt %, a broad unstructured red-shifted emission band appears at ∼580 nm, increasing in intensity relative to the peak at 467 nm by excitation from 365 to 410 nm and only a broad red-shifted band at 635 nm with λ_exc_ > 460 nm. These bands are characteristic of the formation of partial ground-state aggregates formed by π···π stacking and/or Pt···Pt interactions, which are supported by the different excitation spectra obtained by monitoring the distinct emission maxima. Because of these contributions, the emission color shifts gradually from green to yellow-orange with increasing concentration (Figure 6), and the quantum yields increase from ϕ = 5% (PS 1 wt %, monomer) to 19% (PS 10 wt %), although without a significant effect in the lifetimes, which fit to two components (τ_average_ = 0.6–0.9 μs).

Only **1b** is emissive in fluid CH_2_Cl_2_ (5 × 10^–4^ M) at 298 K (Figure S20), displaying a typical structured phosphorescent emission, indicative of an emissive state of mixed ^3^IL/^3^MLCT nature with a dominant ^3^IL character, which was further supported by calculations (see below). No significant changes neither in emission maxima nor the quantum yield were observed in a THF solution at 298 K (Table S10). Complex **1b** shows an aggregation of the Pt^II^ monomer in glasses of CH_2_Cl_2_ even in a diluted solution (5 × 10^–5^ M, λ_em_ 640 nm), whereas in THF glasses, the presence of the monomer (λ_em_ 517 nm, Figure S20) is dominant. The effect of the concentration on the emissive behavior was examined in CH_2_Cl_2_ at 298 and 77 K. The distinct emission profiles and color change can be visualized in Figure S21. At 298 K, increasing the concentration (5 × 10^–5^ to 0.4 M) affects the emission color. With concentrations of 5 × 10^–5^ to 0.01 M, only the monomer is observed. From 0.01 to 0.4 M, an aggregation broad peak (λ_em_ ∼ 710 nm), attributed to metal–metal-to-ligand charge transfer (^3^MMLCT), gradually gains intensity, and the emission changes from green to red (Figure S21a). Upon cooling to 77 K, the monomer emission dominates in diluted solutions (5 × 10^–5^ to 10^–3^ M), whereas that aggregated emission peak is observable from 0.01 to 0.4 M, together with the monomer (Figure S21b).

**Figure 7:**
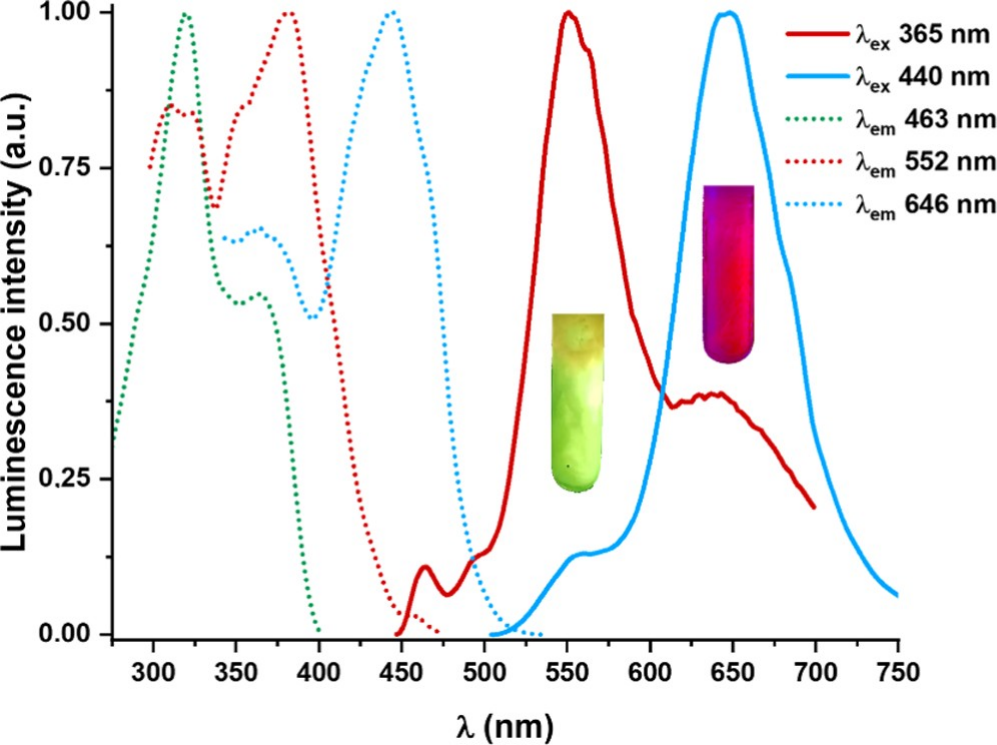
Normalized excitation and emission spectra of **1a** in CH_2_Cl_2_ 5 × 10^–5^ M at 77 K. Photographs taken under lamps of the ∼λ_exc_ indicated and the N_2_ atmosphere.

Complex **1a** is nonemissive in solution probably due to deactivation through molecular motions in fluid or the ease of the thermal activation of the ^3^MC excited states. In a glassy solution at 77 K, it exhibits intense emission profiles, which depend on the solvent, the concentration, and the wavelength excitation. Upon cooling, diluted CH_2_Cl_2_ solutions (5 × 10^–5^ M) show three different emission bands depending on the excitation wavelengths (Figure 7). By excitation to low wavelengths (λ_exc_ 365 nm), a minority band of the monomer (λ_em_ 465 nm), a broad band at 552 nm, and a shoulder at 645 nm are observed, whereas with λ_exc_ > 440 nm, the band at 645 nm dominates. The excitation spectra monitored at the three peaks differ, also suggesting the aggregation processes. At higher concentrations (5 × 10^–4^ M), only two broad emission bands (λ_em_ 570 and 650 nm) are observed by excitation at λ_exc_ < 400 nm, the LE emission band being selectively obtained using LE excitation wavelengths (λ_exc_ 450 nm) (Figure S22). Considering the unstructured emission profiles and their crystal packing, we tentatively ascribe the origin of these LE bands to ligand-centered ^3^ππ* and ^3^MMLCT transitions, respectively. Similar behavior was observed in THF at 77 K (Figure S23). In the glasses, the aggregate lifetimes [τ = 9.7 μs (552), 6.4 μs (645 nm) **1a**, CH_2_Cl_2_ 5 × 10^–5^ M; 10.1 μs (640 nm) (5 × 10^–5^ M), 9.7 μs CH_2_Cl_2_ 0.01 M, **1b**] are shorter than the corresponding monomer decay [τ = 14.9 μs (465), **1a**, CH_2_Cl_2_; 14.8 μs (540) CH_2_Cl_2_**1b**], in agreement with ^3^MMLCT contribution for the lowest-energy band (645 **1a**, 710 nm **1b**) or some triplet–triplet annihilation (TTA)^25^ in the ^3^ππ* excimeric-like feature (552 **1a**, 640 nm **1b**).

**Figure 8:**
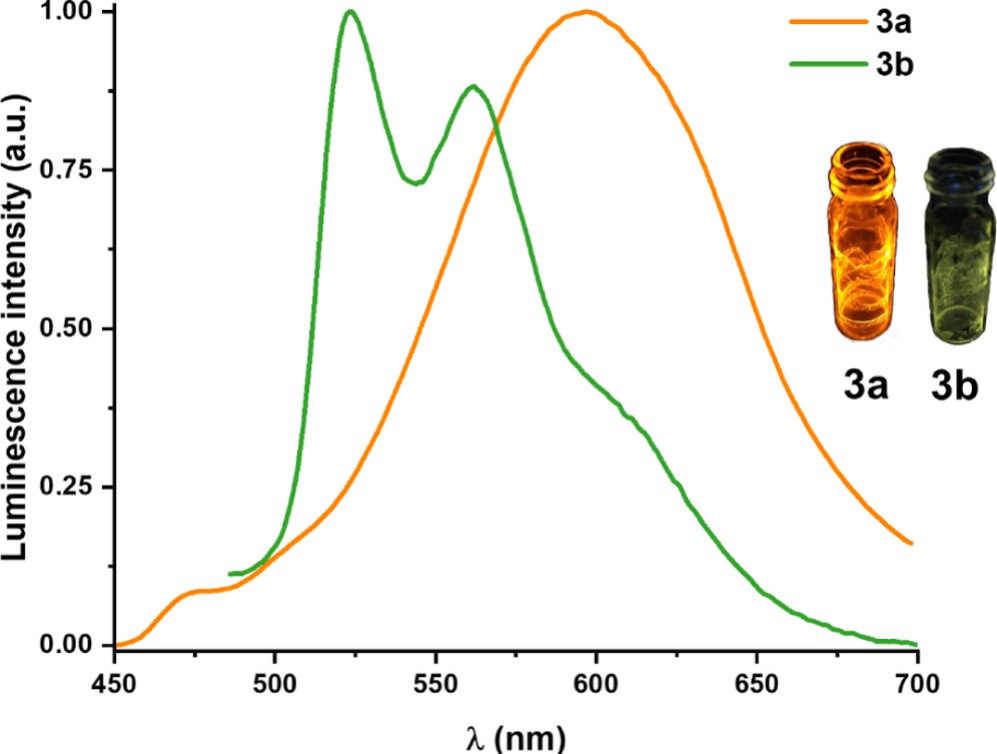
Normalized emission spectra of **3a** (λ_ex_ 365 nm) and **3b** (λ_ex_ 420 nm) in PS (1 wt %) at 298 K in air.

For the bis-isocyanide compounds in PS films (1 wt %), **3b** displays a typical ^3^IL-structured emission (524 nm), similar to **1b**, while in the dfppy derivative (**3a**) dominates an unstructured emission peak [600 nm, τ = 1.8 (61%), 3.0 (39%) μs], characteristic of aggregate emission, in relation to the small intensity of the monomer (474 nm) (Figure 8). In a fluid CH_2_Cl_2_ solution (5 × 10^–4^ M) (Figures S24a and S25a) both compounds show monomer emission (471 **3a**, 511 nm **3b**) with lower quantum yields (ϕ 1% **3a**, 2% **3b**) than in PS films (ϕ 27% **3a**, 9% **3b**). In agreement with its behavior in PS films, compound **3a** shows a higher tendency to aggregate in glasses at 77 K (Figure S24b), whereas **3b** displays essentially monomeric emission with an increase in the LE shoulder (Figure S25b). As in complexes **1**, the aggregate lifetimes [τ = 6.6 (580) **3a**, 14.5 (618) μs **3b**, CH_2_Cl_2_ 77 K] are shorter than the corresponding monomer [τ = 39.1 (475) **3a**, 24.5 (524) μs **3b**, CH_2_Cl_2_ 77 K].

**Figure 9:**
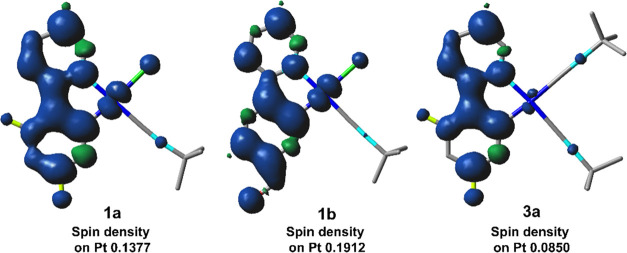
Spin density distribution for the lowest triplet excited state in **1a**, **1b**, and **3a**.

The analysis of the monomer emission of these compounds indicates that the variation of the cyclometalated ligand has a notable effect on the emission maximum, with the ppy-CHO complexes (**b**) red-shifted with respect to the dfppy (**a**) derivatives, consistent with the higher energy gap for the π–π* orbitals of the dfppy complexes. The nature of the emissions was studied in **1a**, **1b**, and **3a** through calculations of the lowest-lying T_1_ (S_0_ → T_1_) (Table S9) and spin density distribution for the triplet excited states (T_1_) (Figure 9), based on their corresponding optimized S_0_ and T_1_ geometries, respectively. The calculated lowest-lying T_1_ state involves mainly the HOMO → LUMO transition (66% **1a**, 77% **1b**, 83% **3a**), resulting in a ^3^IL transition with a minor contribution of ^3^MLCT and ^3^XLCT for complexes **1** and ^3^LL′CT (C^∧^N → CNBu*^t^*) and ^3^LMCT (C^∧^N → Pt) for **3a**. However, the calculated spin density distribution in the optimized T_1_ state (Figure 9) is located mainly on the cyclometalated ligand and to a lesser extent in platinum (Pt, 0.1377 **1a**, 0.1912 **1b**, 0.0850 **3a**), with a negligible contribution of the other coligands, thus supporting a predominant ^3^IL state with ^3^MLCT contribution higher in the neutral complexes **1** than in **3**. This result is in coherence with numerous works on cyclometalated isocyanide metal complexes, in which the large separation between the ^3^MLCT and ^3^IL states lead to a weak configuration interaction and a predominant ^3^IL character in T_1_.^26^ In agreement with this, the calculated contribution of the Pt center in the SOMO-1 (Figure S26 and Table S11) decreases (Pt 9% **1a**, 16% **1b**, 3% **3a**) in the optimized T_1_ state in relation to the optimized S_0_ geometry, especially for **1** (HOMO, 30% **1a**, 34% **1b**, 5% **3a**), suggesting distortion upon excitation. The calculated emission wavelengths [**1a** (521), **1b** (619), **3a** (531 nm)] are consistent with the monomer emission observed [**1a** (467), **1b** (524), **3a** (474 nm), PS 1 wt %], although with expected overestimated values.

### Emission Spectra in the Solid State

The photophysical characteristics in the solid state are compiled in Tables 2 (emission) and S12 (absorption spectra calculated from their reflectance spectra). Interestingly, complexes **1a** and **1b** exhibit multistimuli behavior, and **3a** shows vapochromic behavior. As is seen in the structural section, the different possibility of packing of the complexes seems to be decisive in the observed vapochromic (**1a**, **1b**, **3a**), solvatochormic, and mechanochromic (**1a**, **1b**) behavior.

**Figure 10:**
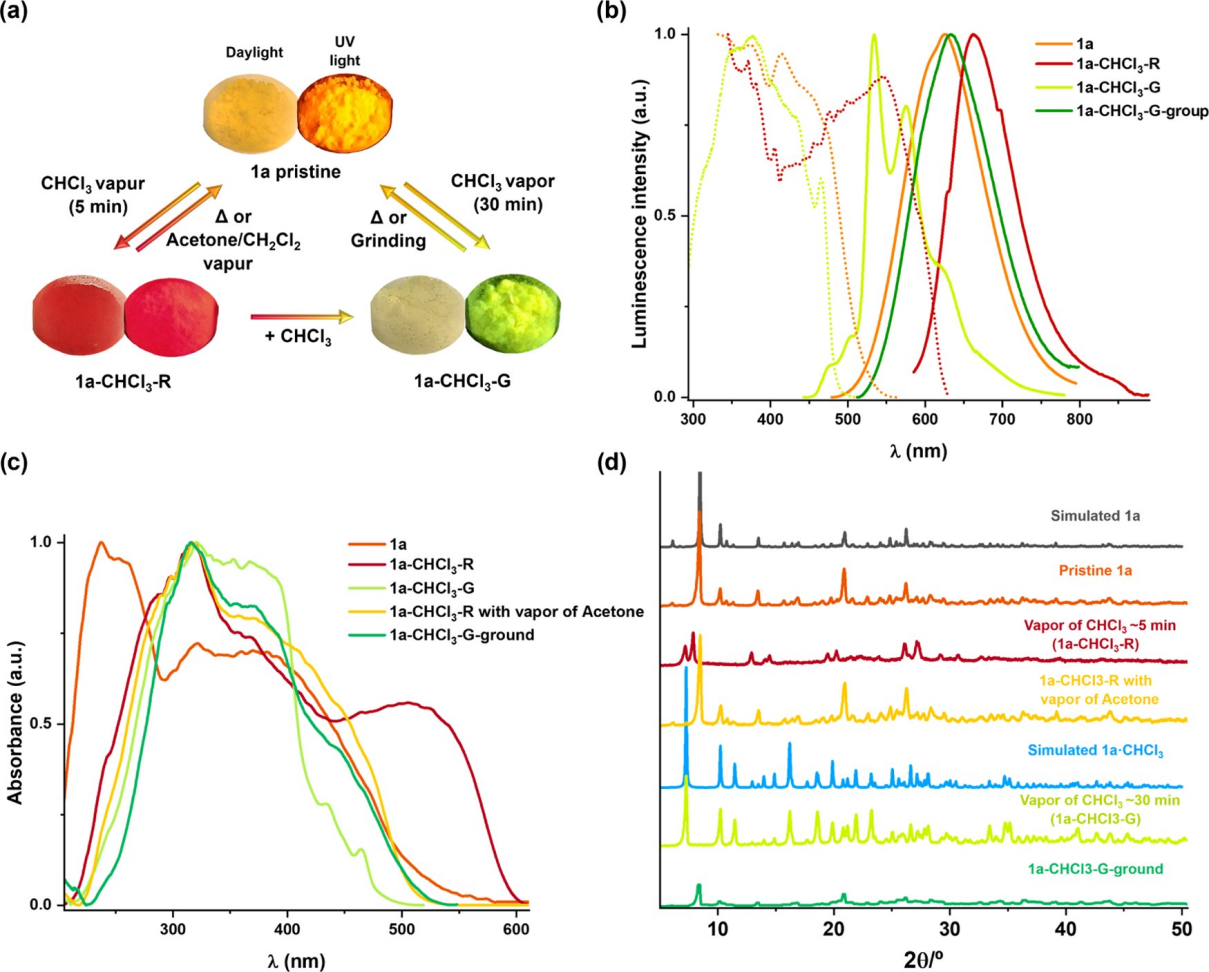
(a) Scheme and photographs of the external stimuli-responsive color and emission changes in **1a**; left: under ambient light and right: under UV light irradiation (λ_ex_ 365 nm). Emission color changes of **1a** after exposure to CHCl_3_ vapors for 5 min (**1a·CHCl**_**3**_**-R**) or more than 30 min (**1a·CHCl**_**3**_**-G**). (b) Normalized excitation (red, λ_em_ 660 nm; orange λ_em_ 625 nm; green λ_em_ 535 nm) and emission spectra of **1a** powder (λ_ex_ 420 nm), **1a·CHCl**_**3**_**-R** (λ_ex_ 550 nm), **1a·CHCl**_**3**_**-G** (λ_ex_ 400 nm), and **1a·CHCl**_**3**_**-G-ground** (λ_ex_ 450 nm) in air. (c) Normalized absorption spectra calculated from their reflectance spectra in the solid state. (d) Powder XRD (PXRD) patterns of different forms of **1a**.

Powdered as-obtained yellow-orange samples or crystals of **1a** show a bright orange structureless emission band (625 nm), narrower and red-shifted (650 nm) to 77 K (Figures 10 and S27), which is associated with a mixed ^3^MMLCT/^3^ππ* manifold due to the formation of dimers in the ground-state stacking in columns with relatively close Pt···Pt and π···π contacts. However, crystals of **1a·CHCl**_**3**_ display a yellow-green monomer ^3^IL/^3^MLCT emission (534 nm) with a minor structured ^3^IL band at 474 nm, which dominates at 77 K (Figure S28), in accordance with their staggered columnar packing with longer Pt···Pt separations. Interestingly, upon fuming the yellow-orange powder **1a** with vapors of CHCl_3_ for only ∼5 min, the sample changes to a kinetic red form, with a red emission (**1a-CHCl**_**3**_**-R** form) (Figures 10a and S29). The elemental and thermogravimetric analyses (TGA) of the **1a-CHCl**_**3**_**-R** form revealed a phase transition at 75 °C arising from the desorption of CHCl_3_ from the lattice (Figure S30a). When a red sample of **1a-CHCl**_**3**_**-R** was heated up to 175 °C, the lost weight (*ca*. ∼19%) corresponds to about one lattice CHCl_3_ molecule per complex, giving **1a**. This **1a-CHCl**_**3**_**-R** form displays a red shift structureless band centered at 662 nm, which narrows and considerably shifts to lower energies (λ_max_ 730 nm, λ_exc_ 580 nm) at 77 K (Figure S29). The emission is attributed to an ^3^MMLCT transition from long-range kinetic aggregates (trimers, tetramers, etc.) formed by fast incorporation of the solvent and likely having shorter Pt···Pt intermolecular interactions than that found in **1a**. Interestingly, CHCl_3_ is easily removed in this kinetic red form (**1a-CHCl**_**3**_**-R**), and upon treatment with acetone or CH_2_Cl_2_ vapors, the unsolvated **1a** is recovered. By contrast, if the fuming time of **1a** with vapors of CHCl_3_ increases to ∼30 min at 298 K, we observed that the color changes gradually from red (**1a-CHCl**_**3**_**-R**) to a final thermodynamically more stable yellow form (**1a-CHCl**_**3**_**-G)** (Figure 10a), with a green-yellow structured emission identical to that measured for crystals **1a·CHCl**_**3**_. TGA analysis shows a phase transition from **1a-CHCl**_**3**_**-G** at 75 °C, corresponding to the transformation to **1a**, by the loss of CHCl_3_ (Figure S30b). If the solid **1a-CHCl**_**3**_**-G** is kept in a close vial, it is stable for weeks, but if it is heated to 65 °C, it reverts to **1a**, although without passing through the previous kinetic red form. Interestingly, the color of the phase **1a-CHCl**_**3**_**-G** changed from pale-yellow to dark yellow by manual grinding in a mortar (Figure S31). The **1a-CHCl**_**3**_**-G-ground** form is an amorphous orange emissive phase, which displays a broad unstructured band at 632 nm, similar to the as-prepared powder **1a**, which is red-shifted at 77 K (677 nm). This indicates that mechanical stress in the yellow phase favors the ground-state interchromophore interactions. The emission quantum yields of these forms range from ϕ 11% in **1a-CHCl**_**3**_**-G**, 21% **1a-CHCl**_**3**_**-R**, and 38% **1a-CHCl**_**3**_**-G-ground** to 51% **1a** (Table 2). These values are comparable to or higher than those in polystyrene.

The absorption properties of all forms in the solid state are shown in Figure 10c and Table S12. The yellow-orange pristine solid **1a** shows a broad absorption band up to 550 nm, whereas the **1a-CHCl**_**3**_**-G** form is characterized by a blue-shifted absorption band up to 500 nm, attributed to mixed ^1^LLCT/^1^MLCT transitions. The **1a-CHCl**_**3**_**-R** phase shows an absorption spectrum extending to 600 nm, most likely due to ^1^MMLCT transitions based on Pt···Pt interactions, in coherence with its color. As expected, the absorption spectrum of **1a-CHCl**_**3**_**-G-ground** and **1a-CHCl**_**3**_**-R** treated with acetone vapors are similar to that obtained for pristine **1a**. The powder X-ray diffraction (PXRD) patterns show that pristine **1a** is crystalline in nature, the PXRD peaks being consistent with the major diffraction peaks simulated from the cif files of the structure of larger single crystals of **1a** (Figure 10d), suggesting that microcrystalline solid has a molecular packing similar to that of their larger crystalline samples. After exposing the sample of **1a** to CHCl_3_ fumes for 5 min to obtain the red form **1a-CHCl**_**3**_**-R**, new peaks appeared (red line), indicating that the incorporation of a molecule of CHCl_3_ induces a structural transformation. The peaks of the pristine sample **1a** can be mainly recovered once the red sample **1a-CHCl**_**3**_**-R** is exposed to acetone or CH_2_Cl_2_ vapors or is heated above 75 °C (yellow line, Figure 10d), indicating that both phases are dynamically related and that the positions of CHCl_3_ are not occupied by the incoming solvents. This behavior suggests the ease of loss of CHCl_3_ either by displacement with more volatile vapors or by heating above the boiling point of CHCl_3_ (61 °C). The vapochromic luminescent behavior of **1a** in CHCl_3_ and **1a-CHCl**_**3**_**-R** in acetone is reversible for at least five cycles without perceivable performance degradation (Figure S32). Furthermore, when the red sample **1a-CHCl**_**3**_**-R** is exposed to air without stimuli, it gradually changes its color and emissive behavior from red to yellow-orange (more than 2 weeks), indicating that the desolvated **1a** species is recovered. On the other hand, the PXRD peaks of the pale-yellow solid **1a-CHCl**_**3**_**-G**, obtained by treatment of **1a** with vapors of CHCl_3_ for ∼30 min (green line, Figure 10d), show consistency with the major diffraction peaks simulated from the cif file of crystals of **1a·CHCl**_**3**_ (blue line). Finally, the grinding process of **1a-CHCl**_**3**_**-G** induces a considerable decrease in its crystallinity, transforming it into an amorphous phase.

**Figure 11:**
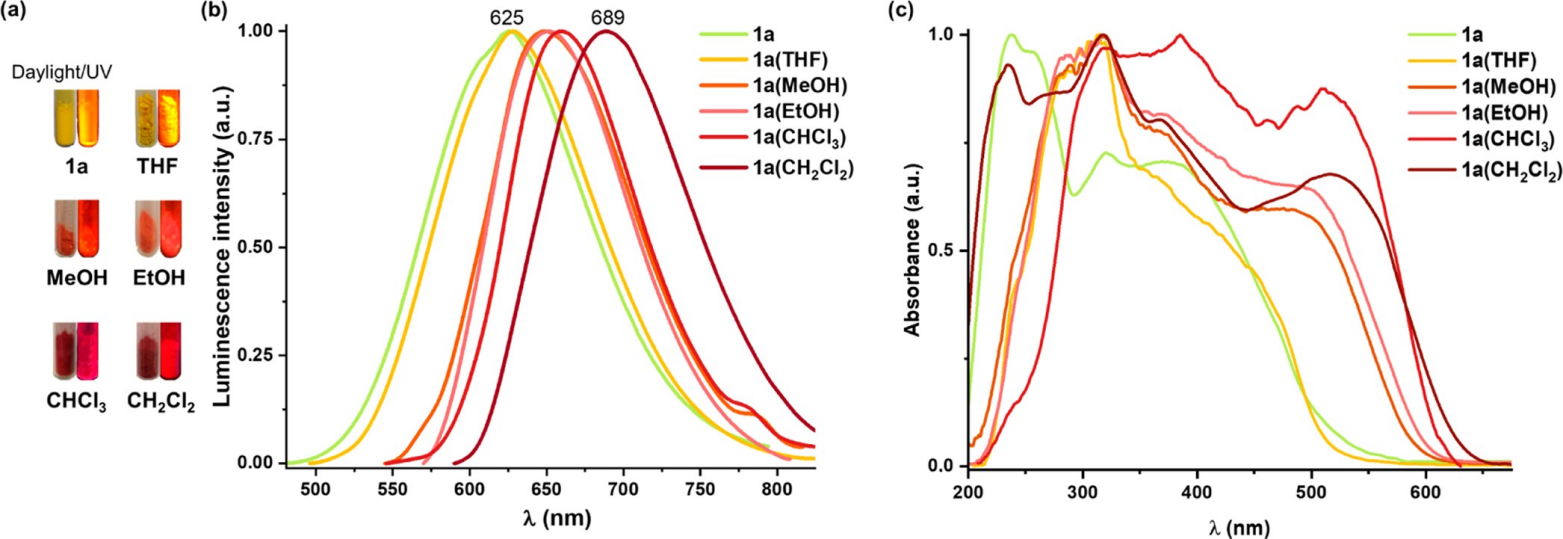
(a) Photographs under ambient light, air atmosphere, and UV light irradiation (λ_ex_ = 365 nm) of **1a** (solid) and solids obtained by the evaporation of the appropriate solution of **1a** in different solvents. (b) Normalized emission spectra. (c) Normalized absorption spectra calculated from their reflectance spectra in the solid state.

In addition to responding to vapors and pressure, complex **1a** displays reversible color, color emission, and quantum efficiency change depending on the crystallization solvents (Figure 11a). The colors of the solids obtained by evaporation after the dissolution of **1a** in different solvents vary from yellow to red and the emission ranges from 627 nm (THF) to 689 nm (CH_2_Cl_2_) (Figure 11b), red-shifted at 77 K (654 nm, THF to 739 nm, CH_2_Cl_2_) (Figure S33 and Table 2). With MeOH or EtOH, the shift observed is ∼25 nm (298 K)/∼70 nm (77 K) and with CHCl_3_ or CH_2_Cl_2_ it reaches up to ∼64 nm (298 K)/∼90 nm (77 K). This discernible behavior can be also observed in the UV–vis absorption solid spectra. The solids obtained from alcohols show, in relation to **1a**, a red-shifted LE band up to 550 nm and from chloride solvents up to 630 nm (Figure 11c and Table S12). The notable red shift of the red solvates could be mainly ascribed to ^1^MMLCT transitions favored by the presence of different aggregates with distinct π···π/Pt···Pt interactions, which seems to be stronger in chloride solvents. The emission color changes are accompanied by a dramatic decrease in the emission brightness from ϕ = 51%, **1a** to 14%, **1a(MeOH)**, typically attributed to aggregation caused quenching. Interestingly, the formed phases in these solvents are not stable with time and the initial form **1a** (emission and quantum efficiencies) can be restored on standing *ca.* 72 h or by stirring the solids in hexane for 1 h. This fact suggests that crystallization from chloride solvents probably gives rise to structures with short Pt···Pt distances, which slowly undergo switching to more stable structures surely with longer Pt···Pt separations.

**Figure 12:**
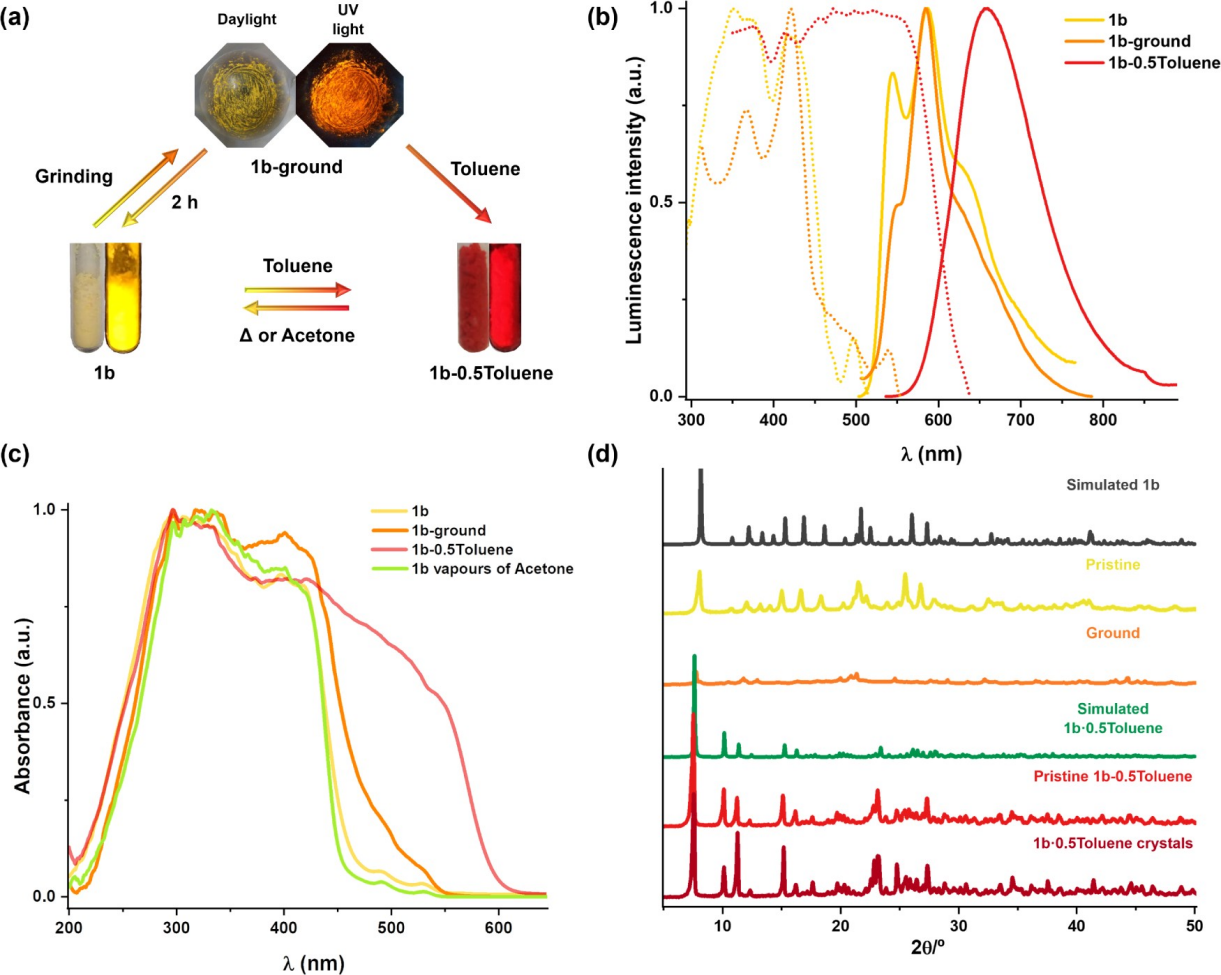
(a) Scheme and photographs of the external stimuli-responsive color and emission changes in **1b**; left: under ambient light and right: under UV light irradiation (λ_ex_ 365 nm). The emission color of **1b** changes under treatment with toluene vapors for 2 h or addition of a drop of toluene (**1b·0.5Toluene**) and by mechanical grinding to afford **1b-ground**. (b) Normalized excitation (yellow, λ_em_ 544 nm; orange, λ_em_ 585 nm; red, λ_em_ 660 nm) and emission spectra of **1b** (λ_ex_ 430 nm), **1b·0.5Toluene** (λ_ex_ 480 nm), and **1b-ground** (λ_ex_ 420 nm) in air. (c) Normalized absorption spectra calculated from their reflectance spectra in the solid state. (d) Powder XRD patterns of different forms of **1b**.

Complex **1b** also exhibits remarkable luminescence switching properties as a result of their self-assembly behavior under application of different external stimuli. The two pseudopolymorphs, **1b** and **1b·0.5Toluene**, can be exchanged under vapor/solvent exposure or heating. Furthermore, a new phase (**1b-ground**), obtained by mechanical grinding of the monomer-emissive form **1b**, is described. The related luminescence images and emission spectra of the three phases are shown in Figure 12a,b. The yellow powder **1b** (and also microcrystals of **1b**) exhibits a yellow structured emission at 545 nm (ϕ = 6%), only slight red-shifted at 77 K (550 nm) and, as expected, with more prolonged lifetime (τ_av_ = 1.5 μs, 298 K; 22.6 μs, 77 K) (Figures 12 and S34). Red microcrystals of **1b·0.5Toluene** show a broad red emission at 658 nm with a ϕ value of 20% and a short lifetime (<1 μs) at 298 K, which is red-shifted at 77 K (706 nm; 9.8 μs) (Table 2 and Figure S35). This emission is ascribed to ^3^MMLCT in accordance with the short Pt··Pt distance (3.362 Å) found in the crystals of **1b·0.5Toluene**. After **1b** was ground, the resulting amorphous orange-yellow powder (**1b-ground**) developed a lesser structured band at 550 nm upon excitation at 420 nm, and a broad LE band at 660 nm by exciting to 500 nm (Figure S36), which are red-shifted at 77 K, indicating the formation of an amorphous solid with closer Pt units. This solid is not stable and **1b** is recovered on standing by 2 h.

When samples of **1b** or **1b-ground** were exposed to toluene liquid or vapors (around 2 h), the yellow powder turned vivid red with red luminescence. The emission spectra displayed an identical pattern to those of the crystals of **1b·0.5Toluene**. The diffuse reflectance spectrum showed a broad band reaching 610 nm, clearly red-shifted in relation to those obtained with samples of **1b** or **1b-ground** tail to 550 nm) (Figure 12c). TGA of **1b·0.5Toluene** revealed the phase transition arising from desorption of the lattice toluene molecules because the TGA curve of up to ∼170 °C represented a weight loss of *ca.* 7.8% corresponding to a half molecule per complex, as the crystal structure of **1b·0.5Toluene** (Figure S37). However, on standing in the solid, the toluene molecules are also lost over time, and after 1 month of exposure to the air, it showed a gradual change to **1b** (Figure S38). Moreover, **1b** is recovered by exposition to acetone liquid/vapors or heating over 110 °C, as is reflected in its color change and its diffuse reflectance spectrum (Figure 12c).

The powder X-ray diffraction pattern of **1b** coincides well with the simulated powder pattern of **1b**_crystal_ (Figure 12d). Mechanical force destroys or changes the ordered molecular packing and induces a crystal-to-amorphous transformation in the PXRD pattern of **1b-ground**, forming a much looser packing where more active molecular motions can occur. A direct crystal-to-crystal phase transition from **1b** to **1b·0.5Toluene** was observed for the PXRD of **1b·0.5Toluene**, obtained from **1b** under saturated toluene vapor or **1b·0.5Toluene**_crystal_, as shown in Figure 12d, which coincides with the simulated PXRD pattern of **1b·0.5Toluene**_crystal_. Toluene fuming can facilitate molecular motions and lead to an arrangement of molecules to form ordered packing again.

**Figure 13:**
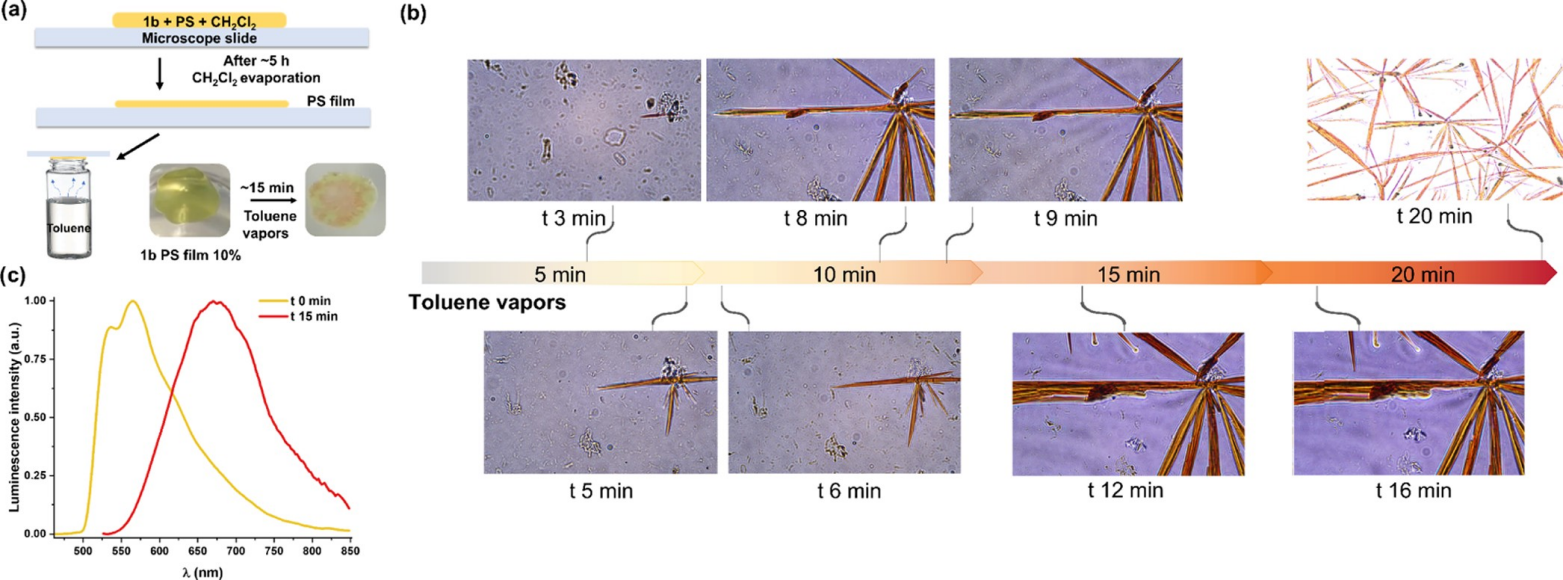
(a) Schematic drawings showing the fabrication of the PS film and the exposition to toluene vapors. Naked eye images of the PS film of **1b** doped at 10% under room light before and after the toluene vapors. (b) Microscopy images of the PS film while the crystals grow by exposure to the solvent for more than 20 min. Images up to 16 min have a magnification of 40× (numerical aperture 0.95), while the image of 20 min has a magnification of 10× (numerical aperture 0.45). (c) Emission color changes of **1b** in a film of PS at 10% (line yellow, λ_ex_ 400 nm) and after exposition to toluene vapors (line red, λ_ex_ 500 nm) at 298 K.

Aggregation of Pt^II^ compounds embedded in polymeric matrices can lead to sensory properties, which are not present in the starting compound. This type of process has been studied in depth for its use as dyes in the fabrication of smart materials.^16a,27^ In this sense, we decided to evaluate the self-assembly behavior of **1b** inside the polymer matrix. Two films of polystyrene (PS) were obtained after doping them with 1 and 10%, respectively, of **1b** in CH_2_Cl_2_. As is shown in Figure 13a, one drop of each mixture was deposited in a glass holder followed by slow evaporation at 298 K (∼5 h). To investigate if the compound **1b** inside the polymer exhibits vapochromic response, the two polymer thin PS films were exposed to vapors of toluene for 20 min, and then their luminescence was measured. For that, the glass with the thin film was placed on top of a vial containing toluene, allowing the vapor to come into contact with the polymer. In the case of the film doped at 1%, it exhibits the typical yellow emission of the monomer species (λ 524 nm). It seems that the low doping is not enough to self-assemble and form aggregates. However, for the higher concentrated film (10%), the change is notable in the presence of toluene vapor both in the naked eye and in the microscope (Figure 13b,c). In the beginning, the polymer film is a yellow-emitting (λ_max_ 566 nm) translucent sheet with some imperfect points. Within 3 min of being exposed to the solvent, orange-red crystalline needles grow inside the matrix from a starting nucleation point. After 15 min, multiple needles with a width of ∼50 μm have grown alongwith the film, showing a remarkable red shift of the emission with the aggregation band centered at 675 nm. This indicates that molecules are self-assembled *via* intermolecular π···π and/or Pt···Pt interactions. The emission of these orange-red needles resembles that of the **1b·0.5Toluene** crystals and the pristine solid **1b·0.5Toluene**, suggesting that the needles are formed by incorporation of toluene channels, as in the crystal packing. The needles are stable for more than 2 months inside the polymer matrix, but after that time, presumably, the toluene gradually is lost (Figure S39), losing its crystallinity and changing its color and emission to yellow.

The double salt **2a** is emissive in the solid state at 298 and 77 K and **2b** only at low temperature. **2a** shows a broad band centered at 640 nm (τ = 0.22 μs, ϕ = 5%), red-shifted to 77 K (717 nm, Δ = 77 nm; τ = 2.1 μs) and in relation to **2b** at 77 K (649 nm) that can be ascribed to a ^3^MMLCT in the salt (Figure S40). Crystals of the cationic bis-isocyanide complex **3a·0.25CH**_**2**_**Cl**_**2**_ displays a monomer green phosphorescence (λ_em_ = 490 nm, ϕ = 15%, τ = 24.8 μs) at 298 K, more structured at 77 K (Table 2 and Figure S41a). However, **3a-pristine** solid shows a bright orange phosphorescence, with very high efficiency (ϕ = 57%) at 298 K, formed by a minor structured band corresponding to the monomer, together with a structureless band at 595 nm, red-shifted at 77 K (617 nm), ascribed to an ^3^MMLCT emission. **3b-pristine** exhibits a broad feature, blue-shifted (λ_em_ = 530 nm) in relation to that observed in **3a-pristine** and with a lower quantum yield (ϕ = 10%), which might be attributed to excimer-like ^3^ππ* emission (Figure S41b). At 77 K, it exhibits a dual emission with maxima at 560 and 628 nm, which is tentatively assigned to excimer-like ^3^ππ* and ^3^MMLCT, respectively. The solid **3a-pristine** showed reversible changes in its color and emission response to vapors of THF, acetone, and CHCl_3_ at room temperature for 6 h. In all cases, there is a change in its color from pale-yellow to pinkish-orange, reflected in the diffuse reflectance spectra, which show new bands extending to ∼600 nm in relation to the **3a**-**pristine** (480 nm). In addition, the emission band is slightly red-shifted from 595 (**3a-pristine**) to 612 (**3a-acetone**), 623 (**3a-THF**), and 627 nm (**3a-CHCl**_**3**_) (Figure S42). At 77 K, the solvate species exhibit a different pattern depending on the excitation wavelength (from 615 to 725 nm), suggesting the formation of different aggregates at low temperatures (Figure S43).

**Figure 14:**
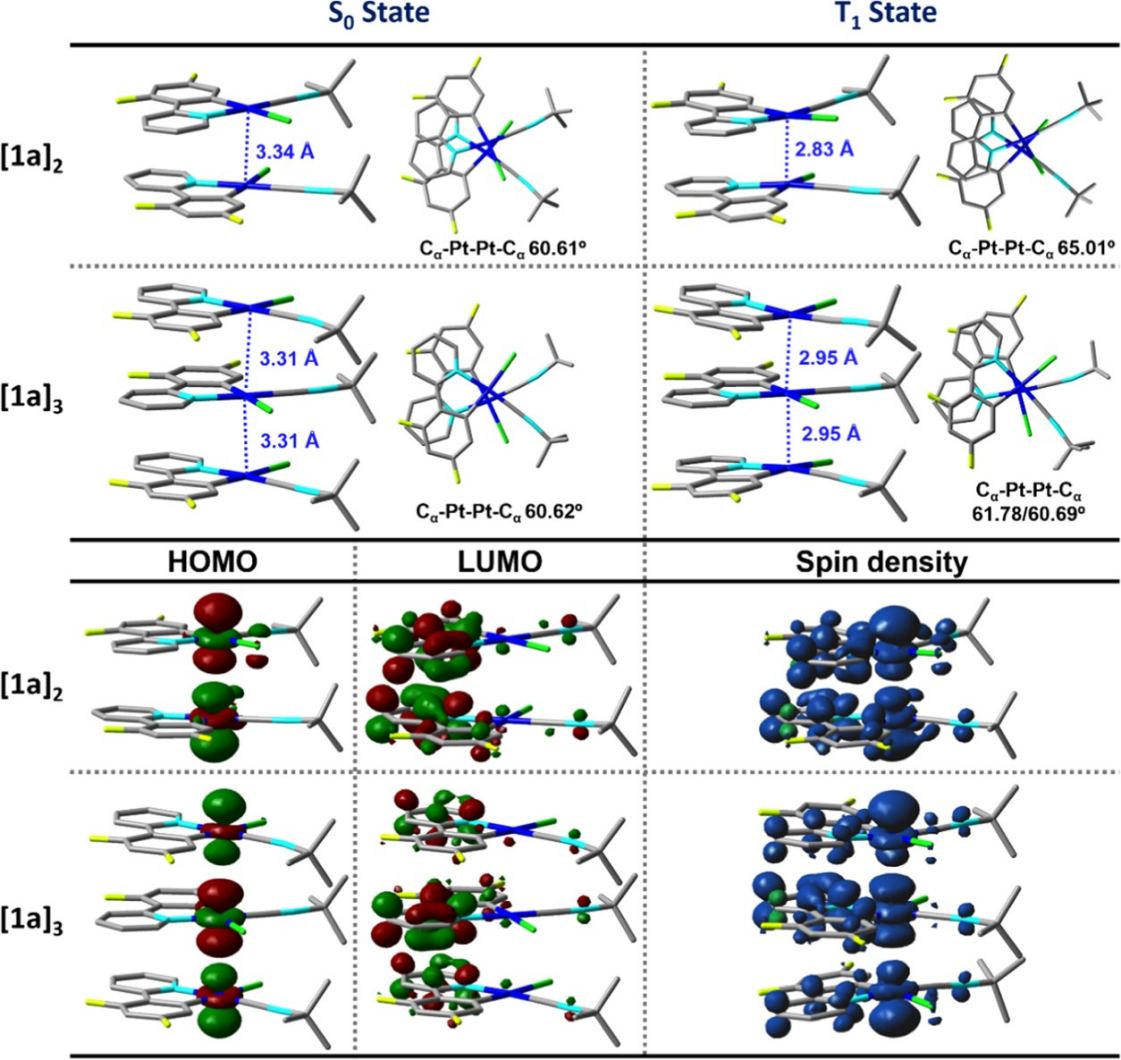
Optimized geometries of **[1a]**_**2**_**and [1a]**_**3**_ models at the S_0_ and T_1_ states. Contour plots of HOMO and LUMO at the S_0_ and spin density at the T_1_-optimized geometries [B3LYP/6–31G(d,p)].

With the aim of understanding the observed red shift in the UV–vis absorption and emission spectra of the aggregated species, the geometries in the ground (S_0_) and the first triplet excited (T_1_) states of the dimer, trimer, and tetramer models were optimized in the gas phase based on **1a** and **1b** crystal structures. Two main intermolecular forces drive the dimer and tetramer assembly, the π···π and Pt···Pt intermolecular interactions. The geometries of calculated structures of **[1a]**_**2**_, **[1a]**_**3**_, and **[1b]**_**4**_ with their Pt···Pt distances and C_α_–Pt–Pt–C_α_ angles and the orbitals involved in the electronic transitions (HOMO/LUMO) as well as the spin density plots are plotted in Figures 14 and S44. For **1a**, the computed Pt···Pt distance of the lowest-energy dimer **[1a]**_**2**_ and trimer **[1a]**_**3**_ in the S_0_ is 3.34 and 3.31 Å, respectively, which is in agreement with the data of X-ray structural analyses. The metallophilic interactions are more evident in the T_1_-optimized geometries (**[1a]**_**2**_ 2.83, **[1a]**_**3**_ 2.95 Å), being shorter than in the X-ray structure. The S_1_ transitions of simulated **[1a]**_**2**_ and **[1a]**_**3**_ were mainly derived of the HOMO → LUMO transitions (>97%, Table S13). The HOMO is located at the Pt atoms (84% **[1a]**_**2**_, 86% **[1a]**_**3**_), whereas the LUMO is mainly localized in the dffpy cyclometalated ligand (84% **[1a]**_**2**_, 82% **[1a]**_**3**_), highlighting the prominent ^1^MMLCT of the low-energy band, red-shifted in relation to the monomer (S_1_ 417 **[1a]**_**2**_, 488 **[1a]**_**3**_*vs* 383 nm **1a**, Table 3). However, in the tetramer model **[1a]**_**4**_, although the Pt···Pt distances are coherent with the X-ray structure, the energy of the low-energy transition with stronger oscillator strength (S_2_ 391 nm) does not fit, and thus this tetramer model is not considered. With respect to the emission, the character of the lowest triplet excited state changes from ^3^IL/^3^MLCT character in the monomer to a mixed ^3^MMLCT/^3^IL in the dimer and trimer, with a higher ^3^MMLCT character for these systems, as is seen in their spin density plots (Figure 14). In agreement with the experimental evidence, the predicted emission maxima are red-shifted in the trimer in relation to the dimer and the monomer, respectively (762 **[1a]**_**3**_, 624 **[1a]**_**2**_*vs* 519 nm **1a**). The value of the trimer, 762 nm, can be compared to the emission of the kinetic red form **1a-CHCl**_**3**_**-R** (662 nm), whereas the value of the dimer fits better to the orange emission of unsolvated crystals **1a** (625 nm, also pristine solid).

**Table 3:** Calculated S_1_ Vertical Excitation Energies and Emission Energy Computed in the Gas Phase (SI)

complex	state	λ (nm)	assignment	Δ*E* emission (T_1_–S_0_ opt) (nm)
**1a**	S_1_	383	HOMO → LUMO (98%)	519
**[1a]**_**2**_	S_1_	417	HOMO → LUMO (98%)	624
**[1a]**_**3**_	S_1_	488	HOMO → LUMO (98%)	762
**1b**	S_1_	430	HOMO → LUMO (97%)	621
**[1b]**_**4**_	S_1_	527	HOMO → LUMO (95%)	943

For **1b**, the optimized geometries of **[1b]**_**2**_ and **[1b]**_**3**_ models do not fit with the experimental X-ray diffraction structure of **1b·0.5Toluene**. **[1b]**_**2**_ and **[1b]**_**3**_ show Pt···Pt distances in their S_0_ of 3.73 and 3.81 Å, respectively, longer than in the 1D chain **1b·0.5Toluene**, although shorter in the T_1_ (2.87 **[1b]**_**2**_, 2.97 Å **[1b]**_**3**_). However, the computed Pt···Pt distances of the lowest-energy tetramer **[1b]**_**4**_ are 3.26 and 3.78 Å, with an orientation of the monomers in perfect agreement with the data of X-ray structural analyses of **1b·0.5Toluene**. As expected, the metallophilic Pt···Pt distances turn significantly shorter in the T_1_-optimized geometry (2.94, 3.03, 3.60 Å). The S_1_ transition of **[1b]**_**4**_ is derived from the HOMO → LUMO transition (95%), the HOMO being composed mainly of Pt (81%) and the LUMO of the ppy-CHO ligand (88%). This evidences the red-shifted low-energy absorptions in **[1b]**_**4**_ (527 nm) with significant MMLCT in relation to the monomer (S_1_ 430 nm). In relation to the emission, the predicted emission maxima of 943 nm, arising from the lowest-energy tetramer **[1b]**_**4**_*vs* 621 nm, obtained for the monomer evidence a high ^3^MMLCT contribution for the lowest triplet excites state, as is reflected in its spin density (Figure S44). These results confirm that the calculated red-shifted absorption and emission are induced by a packing arrangement better in dimers and trimers in **1a** and tetramers in **1b**, which improves the intermolecular interactions, leading to an increased MMLCT character.

## Conclusions

In summary, we report the synthesis, characterization, and photophysical investigations of new phosphorescent *tert*-butylisoyanide Pt^II^ complexes with difluorophenylpyridine or formyl-functionalized phenylpyridine cyclometalated (C^∧^N = dfppy **a**, ppy-CHO **b**) ligands: neutral, [Pt(C^∧^N)Cl(CNBu*^t^*)] (**1**), double salts [Pt(C^∧^N)(CNBu*^t^*)_2_][Pt(C^∧^N)Cl_2_] (**2**), and cationic [Pt(C^∧^N)(CNBu*^t^*)_2_]ClO_4_ (**3**). A comparative study of the crystal packing of the pseudopolymorphs of **1a** (**1a**, **1a·CHCl**_**3**_), **1b** (**1b**, **1b·0.5Toluene**, **1b·0.5PhF**), and **3a·0.25 CH**_**2**_**Cl**_**2**_ shows a great ability to be assembled by intermolecular Pt···Pt and/or π···π interactions in the solid state, so generating 1D columnar stackings. The structures with head-to-tail packing exhibit larger Pt···Pt distances (4.556–6.541 Å) than the structures with a head-to-head disposition (3.362–3.896 Å), which is reflected in a change of the emission from greenish or yellow to orange/red or red. We discovered that complexes **1a** and **1b** exhibit multistimuli-responsive properties, displaying vapochromism, vapoluminescence, solvatochromism, thermochromism, and mechanochromism in the solid state, which can be attributed to modulation of Pt···Pt and/or π···π interactions with distinct color switching from green to red. The solvates **1a·CHCl**_**3**_, **1b·0.5Toluene**, and **1b·0.5PhF** contain channels running parallel to the 1D columns, occupied by the corresponding solvent, thus rationalizing the vapochromic/vapoluminescent/solvatochromic response of solvent-free **1a** and **1b** materials. The interactions can be established even in a polymer matrix. Thus, **1b** undergoes also supramolecular self-assembly *via* Pt···Pt and/or π···π interactions into a polystyrene film (PS, 10 wt %) in response to toluene vapors. Grinding the pristine solids **1a** and **1b** affects their molecular packing in the solid state, producing a color emission change to orange with the loss of crystallinity (PXRD). Theoretical simulations confirm that Pt···Pt contacts are relevant in dimer and trimer geometries in **1a** and tetramers in **1b**, especially in their T_1_ states, leading to an increased MMLCT character in the solid-state aggregates.

## Supporting Information Available

Experimental section and tables and figures giving spectroscopic, structural, photophysical, and theoretical data for compounds prepared in this paper (PDF)
