# Supplementary material for: Multistimuli-Responsive Properties of Aggregated Isocyanide Cycloplatinated(II) Complexes
Source: Inorg Chem. 2022 Jul 1;61(28):10898–914. doi: 10.1021/acs.inorgchem.2c01400 (PMC9348835; doi:10.1021/acs.inorgchem.2c01400)
Supplement: Supplementary file 1 — ic2c01400_si_001.pdf [file ic2c01400_si_001.pdf]

Supporting Information

Multi-Stimuli Responsive Properties of Aggregated Isocyanide Cycloplatinated(II) Complexes

Mónica Martínez-Junquera, Elena Lalinde and M. Teresa Moreno**

Departamento de Química-Centro de Síntesis Química de La Rioja, (CISQ), Universidad de La Rioja, 26006, Logroño, Spain. E-mails: elena.lalinde@unirioja.es teresa.moreno@unirioja.es

Contents: Page

1.- Experimental Section……………………………………………………... S2
2.- Characterization of complexes……………………………………………… S9
3.- Crystal Structures…………………………………………………………… S15
4.- Photophysical Properties and Theoretical calculations…………………... S24

1.- Experimental section

General comments. All reactions were carried out under an atmosphere of dry N_2, using standard Schlenk techniques. Solvents were obtained from a solvent purification system (M-BRAUN MB SPS-800). Elemental analyses were carried out with a Carlo Erba EA1110 CHNS-O or a Perkin-Elmer CHNS/O 2400 Series II microanalyzer. Mass spectra were recorded on a Microflex MALDI-TOF Bruker (MALDI) spectrometer operating in the linear and reflector modes using dithranol as matrix or on a HP-5989B mass spectrometer (ESI). IR spectra of powders (**3**) were obtained on a Perkin Elmer Spectrum UATR Two FT-IR Spectrometer with the diamond crystal ATR accessory covering the region between 4000 and 450 cm^{-1}. IR spectra of **1** and **2** (Nujol emulsions) were obtained on a Nicolet Nexus FT-IR Spectrometer covering the region between 4000 and 200 cm^{-1}. NMR spectra were recorded on a Bruker Avance 400 spectrometer at 293 K. Chemical shifts are reported in parts per million (ppm) relative to external standards ($SiMe_4$), and all coupling constants are given in hertz (Hz). NMR labelling is given in Scheme 1. The UV−vis absorption spectra were measured with a Hewlett-Packard 8453 spectrophotometer. Diffuse Reflectance UV-vis (DRUV) spectra were carried out in SiO_2 pellets, using a Shimazdu UV-3600 spectrophotometer with a Harrick Praying Mantis accessory, and recalculated following the Kubelka Munk function. Excitation and emission spectra were obtained in a Shimazdu RF-60000. The measurements in solid state and PS films were carried out on air and in solutions under N_2 atmosphere. The lifetime measurements up to 10 μs at 298 K at all samples at 77 K were performed with a Jobin Yvon Horiba Fluorolog operating in the phosphorimeter mode (with an F1-1029 lifetime emission PMT assembly, using a 450 W Xe lamp) and the Jobin Yvon software packing, that works with Origin 6.0. The decay data were analysed by tail fitting to the functions "One-phase exponential decay function with time constant parameter" (ExpDec1) and "Two-phase exponential decay function with time constant parameters (ExpDec2)". The lifetimes below 10 μs at 298 K were measured with a Datastation HUB-B with a nanoLED controller, using the technique "Time Correlated Single Photon Counting" (TCSPC). The nanoLEDs employed for lifetime measurements were of 450, 390 and 370 nm with pulse lengths of 0.8–1.4 ns. The decay data were treated with the software DAS6 (Jobin Yvon-Horiba). In our hands, the estimated uncertainty in the phosphorimeter mode is ±10% or better and with the nanoLEDs is below ±5% of the quoted value. Quantum yields of solids, solutions and PS films were measured using a Hamamatsu Absolute PL

Quantum Yield Measurement System C11347-11. For all measurements (τ and ϕ), the emission and/or excitation used are those indicated in each case in the Tables 2 and S10. The estimated uncertainty in the quantum yield in this way on the instrumentation employed is in our hands of ~±1% for very emissive samples and below ±5% for weak emissive samples. The powder X-ray diffraction (XRD) patterns were obtained at room temperature by using a Rigaku Miniflex II with graphite-monochromated CuK_{α} operating at 30 kV and 15 mA. PXDR patterns were collected between 2θ of 3° and 60° with a 2θ stepping angle of 0.03 and an angle dwell of 1s. Thermogravimetric analysis (TGA) was performed under the N_2 atmosphere using a SDT2960 Simultaneous DTA-TGA instrument at atmospheric pressure with a 5 °C/min heating rate. All digital images of pictures of the vapochromic changes and crystals were acquired by using a Nikon Eclipse Ti2 microscope and a Photometrics prime 95B 25 mm camera with objectives at 10x magnification (numerical aperture 0.45) and 40× (numerical aperture 0.95). The images were automatically stitched by the Nikon NIS-Elements AR image analysis software. The microscope was attached to an Edinburgh FLS 1000 fluorescence spectrometer where their luminescent spectra were obtained. Complexes [Pt(dfppy)(Hdfppy)Cl],[1] $[Pt(dfppy)(\mu\text{-}Cl)]_2$ [2] and $[Pt(ppy\text{-}CHO)(\mu\text{-}Cl)]_2$ [3] was prepared as reported in the literature. Other commercially available reagents were used as received.

Preparation of [Pt(ppy-CHO)(Hppy-CHO)Cl] (Ib). It was prepared using the same procedure as complex [Pt(dfppy)(Hdfppy)Cl],[1] by reaction of K_2PtCl_4 (1.804 g, 4.347 mmol) with 2 equiv. of 4-(2-pyridyl)benzaldehyde (1.593 g, 8.694 mmol) in a mixture of 2-ethoxyethanol and water (3:1, v/v; 50 mL) at 80 ºC for 12 h. After cooling, the resulting orange precipitate was filtered off and washed consecutively with water (15 mL), ethanol (10 mL) and diethyl ether (10 mL) to give an orange solid (2.214 g, 85 %). IR (cm^{-1}): ν(C=O) 1635 (vs). ESI(+): *m/z* (%): 560 $[M\text{-}Cl]^+$ (100). Anal. Calcd for $C_{24}H_{17}ClN_2O_2Pt$ (595.95): C, 48.37; H, 2.88; N, 4.70. Found: C, 48.98; H, 2.96; N, 5.00. ^1H NMR (400 MHz, $CDCl_3$, δ) (tentatively assigned): 9.92 (s, $CHO_{ppy\text{-}CHO}$), 9.75 (s, $CHO_{Hppy\text{-}CHO}$), 9.68 (d, $J_{H\text{-}H} = 6.3$, $^3J_{Pt\text{-}H} = 50$, $H^2_{ppy\text{-}CHO}$), 9.25 (d, $J_{H\text{-}H} = 5.8$, $^3J_{Pt\text{-}H} = 46$, $H^2_{Hppy\text{-}CHO}$), 8.21 (d, $J_{H\text{-}H} = 7.7$, 2H, $H^{9/11}_{Hppy\text{-}CHO}$), 8.07 (t, $J_{H\text{-}H} = 7.3$, $H^4_{Hppy\text{-}CHO}$), 7.84 (t, $J_{H\text{-}H} = 7.5$, $H^4_{ppy\text{-}CHO}$), 7.78 (d, $J_{H\text{-}H} = 7.7$, 2H, $H^{8/12}_{Hppy\text{-}CHO}$), 7.72 (d, $J_{H\text{-}H} = 7.7$, $H^5_{Hppy\text{-}CHO}$), 7.63 (d, $J_{H\text{-}H} = 8.2$, $H^5_{ppy\text{-}CHO}$), 7.50 (t, $J_{H\text{-}H} = 7.3$, $H^3_{Hppy\text{-}CHO}$), 7.47-7.44 (m, H^9, H^8), 7.21 (t, $J_{H\text{-}H} = 6.4$, $H^3_{ppy\text{-}CHO}$), 6.69 (s, $^3J_{Pt\text{-}H} = 47$, $H^{11}_{ppy\text{-}CHO}$).

Preparation of [Pt(dfppy)Cl(CNBu^t)] (1a). To a yellow suspension of [Pt(dfppy)(Hdfppy)Cl] (0.665 g, 1.087 mmol) in CH_2Cl_2 (10 mL), CNBu^t (0.122 mL, 1.087 mmol) was added to give an orange-yellow solution. After 90 min of stirring, the solvent was evaporated to dryness. The residue was treated with 30 mL of toluene and an insoluble red solid (0.050 g, 4.5 %) was filtered, which was proposed as the double salt **[Pt(dfppy)(CNBu^t)$_2$][Pt(dfppy)Cl$_2$] (2a)**. The filtrate was evaporated to dryness and the yellow residue was treated with hexane (15 mL) to give **1a** as a yellow-orange solid (0.481 g, 88 %). Compound **1a:** IR (cm^{-1}, Nujol): ν(C≡N) 2207 (vs); ν(Pt-Cl) 289 (w). ESI (+): *m/z* (%): 468 [M-Cl]$^+$ (100), 971 [2M-Cl]$^+$ (37). Anal. Calcd for $C_{16}H_{15}ClF_2N_2Pt$ (503.84): C, 38.14; H, 3.00; N, 5.56. Found: C, 38.49; H, 3.40; N, 6.07. ^{1}H NMR (400 MHz, $CDCl_3$, δ): 9.47 (d, $J_{H\text{-}H}$ = 5.8, $^3J_{Pt\text{-}H}$ = 34, H^2), 7.98 (d, $J_{H\text{-}H}$ = 8.2, H^5), 7.84 (t, $J_{H\text{-}H}$ = 7.8, H^4), 7.23 (t, $J_{H\text{-}H}$ = 6.5, H^3), 6.89 (dd, $^3J_{H\text{-}F}$ = 7.9, $^3J_{Pt\text{-}H}$ = 87, H^{11}), 6.5 (ddd, $^3J_{H\text{-}F}$ = 9.2, J = 2.7, H^9), 1.66 (s, 9H, CH_3, Bu^t). ^{13}C{^{1}H} NMR (100.6 MHz, $CDCl_3$, δ): 163.8 (dd, $^1J_{C\text{-}F}$ = 256, $^3J_{C\text{-}F}$ = 12, $^3J_{Pt\text{-}C}$= 122, C$^{10}_{dfppy}$), 163.2 (d, $^2J_{Pt\text{-}C}$ = 104, $^3J_{C\text{-}F}$ = 7.3, C$^6_{dfppy}$), 160.3 (dd, $^1J_{C\text{-}F}$ = 259, $^3J_{C\text{-}F}$ = 13, $^3J_{Pt\text{-}C}$= 86, C$^8_{dfppy}$), 149.0 (s, $^2J_{Pt\text{-}C}$ = 21, C$^2_{dfppy}$), 144.1 (d, $^3J_{C\text{-}F}$ = 7.7, $^1J_{C\text{-}Pt}$ = 1045, C$^{12}_{dfppy}$), 140.5 (s, C$^4_{dfppy}$), 127.9 (m, $^2J_{C\text{-}F}$ = 39, C$^7_{dfppy}$), 122.3 (d, $^4J_{C\text{-}F}$ = 20, $^3J_{Pt\text{-}C}$ = 50, C$^5_{dfppy}$), 122.0 (s, $^3J_{Pt\text{-}C}$ = 26, C$^3_{dfppy}$), 118.5 (dd, $^2J_{C\text{-}F}$ = 49, $^4J_{C\text{-}F}$ = 3.2, $^2J_{Pt\text{-}C}$ = 121, C$^{11}_{dfppy}$), 118.1 (m, $^1J_{Pt\text{-}C}$ = 1808, *C*≡N), 100.4 (t, $^1J_{C\text{-}F}$ = 26, C$^9_{dfppy}$), 58.9 (m, *C*(CH_3)$_3$, Bu^t), 30.3 (s, CH_3 Bu^t). ^{19}F NMR (376.5 MHz, $CDCl_3$, δ): -106.9 (m, $^4J_{F\text{-}Pt}$ = 65, F^{10}), -109.2 (m, $^4J_{F\text{-}Pt}$ = 56, F^8).

Synthesis of [Pt(dfppy)Cl$_2$][Pt(dfppy)(CNBu^t)$_2$] (2a). To a suspension of [Pt(dfppy)(μ-Cl)]$_2$ (0.094 g, 0.111 mmol) in 15 mL of MeOH, (NBu_4)Cl (0.065 g, 0.233 mmol) was added. After 12 h of reflux, the yellow solution formed of (NBu_4)[Pt(dfppy)Cl$_2$][4] was filtered through celite, [Pt(dfppy)(CNBu^t)$_2$]ClO_4 (0.144 g, 0.222 mmol) was added and the solution was concentrated to 5 mL. After 1 h of stirring, the suspension was filtered and **2a** was obtained as a red solid (0.084 g, 36%). Compound **2a:** IR (cm^{-1}, Nujol): ν(C≡N) 2247 (vs), 2213 (vs). MALDI-TOF (+): *m/z* (%): 551 (100) [Pt(dfppy)(CNBu^t)$_2$]$^+$; MALDI-TOF (-): *m/z* (%): 456 (100) [Pt(dfppy)(Cl)$_2$]$^-$. Anal. Calcd for $C_{32}H_{30}Cl_2F_4N_4Pt_2$ (1006.11): C, 38.14; H, 3.00; N, 5.56. Found: C, 38.38; H, 3.06; N, 6.07. Not enough soluble to be characterized by NMR spectroscopy.

Preparation of [Pt(ppy-CHO)Cl(CNBu^t)] (1b). To an orange suspension of [Pt(ppy-CHO)(Hppy-CHO)Cl] (**Ib**) (0.426 g, 0.715 mmol) in CH_2Cl_2 (15 mL), CNBu^t (0.081 mL, 0.715 mmol) was added to give an orange-yellow solution. After 2 h of stirring, the

solvent was evaporated to dryness and treated with 30 mL of toluene, precipitating a yellow solid (0.04 g, 2.2 %), which was filtered and proposed as the double salt **[Pt(ppy-CHO)(CNBu^t)$_2$][Pt(ppy-CHO)Cl$_2$]** (**2b**). The filtrate was evaporated to dryness and treated with 15 mL of *n*-hexane, to give a red solid (0.321 g, 85 %) corresponding to **1b·0.5 Toluene** (confirmed by TGA, elemental analysis and X-ray). When **1b·0.5 Toluene** was dissolved in CH_2Cl_2 (10 mL), evaporated to dryness and treated with *n*-hexane (10 mL), a yellow-orange solid (0.304 g, 83 %) was obtained corresponding to **1b**. IR (cm^{-1}, Nujol): **1b** ν(C≡N) 2205 (vs); ν(Pt-Cl) 289 (w). Anal. Calcd for **1b** $C_{17}H_{17}ClN_2OPt$ (495.87): C, 41.18; H, 3.46; N, 5.65. Found: C, 41.60; H, 3.81; N, 5.95. IR (cm^{-1}) **1b·0.5 Toluene**, ν(C≡N) 2202 (vs). Anal. Calcd for **1b·0.5 Toluene** $C_{20.5}H_{21}ClN_2OPt$ (541.94): C, 45.43; H, 3.91; N, 5.17. Found: C, 45.39; H, 3.59; N, 5.22. ESI (+): *m/z* (%): 460 [M-Cl]$^+$ (47), 956 [2M-Cl]$^+$ (100). ^{1}H NMR (400 MHz, $CDCl_3$, δ): 9.91 (s, CHO), 9.50 (d, J_{H-H} = 5.8, $^3J_{Pt-H}$ = 34, H^2), 7.92-7.86 (m, H^4, $^3J_{Pt-H}$ = 68, H^{11}), 7.75 (d, J_{H-H} = 7.7, H^5), 7.61-7.52 (m, H^9, H^8), 7.31 (t, J_{H-H} = 6.4, H^3), 1.70 (s, 9H, CH_3, Bu^t). $^{13}C\{^1H\}$ NMR (100.6 MHz, $CDCl_3$, δ): 192.2 (s, $CHO_{ppy-CHO}$), 164.6 (s, $^2J_{Pt-C}$ = 94, $C^6_{ppy-CHO}$), 149.8 (s, $^2J_{Pt-C}$ = 37, $C^7_{ppy-CHO}$), 149.2 (s, $^2J_{Pt-C}$ = 21, $C^2_{ppy-CHO}$), 141.1 (s, $^1J_{C-Pt}$ = 1029, $C^{12}_{ppy-CHO}$), 140.4 (s, $C^4_{ppy-CHO}$), 137.3 (s, $^3J_{Pt-C}$ = 67, $C^{10}_{ppy-CHO}$), 136.2 (s, $^2J_{Pt-C}$ = 110, $C^{11}_{ppy-CHO}$), 126.7 (s, $C^9_{ppy-CHO}$), 124.2 (s, $^3J_{Pt-C}$ = 40, $C^8_{ppy-CHO}$), 123.5 (s, $^3J_{Pt-C}$ = 27, $C^3_{ppy-CHO}$), 119.8 (s, $^3J_{Pt-C}$ = 34, $C^5_{ppy-CHO}$), 118.4 (s, $^1J_{Pt-C}$ = 1823, *C*≡N), 59.0 (m, $C(CH_3)_3$, Bu^t), 30.3 (s, CH_3 Bu^t).

Compound **2b**: MALDI-TOF (+): *m/z* (%): 543 (100) [Pt(ppy-CHO)(CNBu^t)$_2$]$^+$; MALDI-TOF (-): *m/z* (%): 447 (100) [Pt(ppy-CHO)Cl$_2$]$^-$. IR (cm^{-1}, Nujol): ν(C≡N) 2242 (s), 2218 (s). Anal. Calcd for $C_{34}H_{34}Cl_2N_4O_2Pt_2$ (991.7): C, 41.18; H, 3.46; N, 5.65. Found: C, 40.98; H, 3.12; N, 5.39. Not enough soluble to be characterized by NMR spectroscopy.

Alternative reaction between (NBu_4)[Pt(ppy-CHO)Cl$_2$] [prepared with [Pt(ppy-CHO)(μ-Cl)]$_2$ and 2 equiv. of NBu_4Cl in MeOH at reflux (12h)] and [Pt(ppy-CHO)(CNBu^t)$_2$]ClO_4 dissolved in acetone, gave a deep yellow solid, which is a mixture of **2b** and **1b**.

Preparation of [Pt(dfppy)(CNBu^t)$_2$]ClO_4 (3a). To a yellow suspension of [Pt(dfppy)(Hdfppy)Cl] (0.303 g, 0.495 mmol) in CH_2Cl_2 (10 mL)/acetone (10 mL) was added excess of $KClO_4$ (0.686 g, 4.95 mmol). After 30 min of stirring, *tert*-butylisocyanide (0.123 mL, 1.088 mmol) (1:2.2) was added to give a red suspension, which gradually became yellow after 2 h. Then, the mixture was evaporated to dryness

and extracted with CH_2Cl_2/H_2O (1/1) (3 x 40 mL). The organic phase was dried over Mg_2SO_4 and filtered through celite. The solvent was removed under reduced pressure and the residue was treated with hexane (20 mL) to afford a pale-yellow solid (0.284 g, 88 %). IR (cm^{-1}): ν(C≡N) 2236 (vs), ν(C≡N) 2215 (vs); ν(ClO_4^-) 1085 (vs), 622 (s). ESI(+): *m/z* (%): 551 (100) $[M-ClO_4]^+$. Anal. Calcd for $C_{21}H_{24}ClF_2N_3O_4Pt$ (650.97): C, 38.75; H, 3.72; N, 5.45. Found: C, 38.50; H, 3.45; N, 5.92. ^1H NMR (400 MHz, $CDCl_3$, δ): 8.90 (d, $J_{H\text{-}H}$ = 5.8, $^3J_{Pt\text{-}H}$ = 39, H^2), 8.14 – 8.02 (m, 2H, H^5, H^4), 7.73 (t, $J_{H\text{-}H}$ = 6.0, H^3), 6.91 (dd, $J_{H\text{-}H}$ = 2.4, $^3J_{H\text{-}F}$ = 7.9, $^3J_{Pt\text{-}H}$ = 68, H^{11}), 6.66 (ddd, $J_{H\text{-}H}$ = 2.4, $^3J_{H\text{-}F}$ = 8.7, $^3J_{H\text{-}F}$ = 11.6, H^9), 1.76 (s, 9H, CH_3, Bu^t), 1.75 (s, 9H, CH_3, Bu^t). $^{13}C\{^1H\}$ NMR (100.6 MHz, $CDCl_3$, δ): 163.6 (dd, $^1J_{C\text{-}F}$ = 258, $^3J_{Pt\text{-}C}$ = 12, C^{10}_{dfppy}), 163.0 (d, $^2J_{Pt\text{-}C}$ = 76, $^3J_{C\text{-}F}$ = 7.3, C^6_{dfppy}), 160.3 (dd, $^1J_{C\text{-}F}$ = 262, $^3J_{Pt\text{-}C}$ = 13, C^8_{dfppy}), 154.7 (s, $^2J_{Pt\text{-}C}$ = 33, C^2_{dfppy}), 152.0 (d, $^3J_{C\text{-}F}$ = 6.9, $^1J_{C\text{-}Pt}$ = 884, C^{12}_{dfppy}), 142.1 (s, C^4_{dfppy}), 131.9 (m, *C*≡$N_{trans\text{-}C}$), 130.3 (m, $^2J_{Pt\text{-}C}$ = 98, C^7_{dfppy}), 126.1 (s, $^3J_{Pt\text{-}C}$ = 33, C^3_{dfppy}), 123.3 (d, $^4J_{C\text{-}F}$ = 22, $^3J_{Pt\text{-}C}$ = 51, C^5_{dfppy}), 119.6 (dd, $^2J_{C\text{-}F}$ = 20, $^4J_{C\text{-}F}$ = 2.7, C^{11}_{dfppy}), 112.0 (d, *C*≡$N_{trans\text{-}N}$), 103.0 (t, $^1J_{C\text{-}F}$ = 26, C^9_{dfppy}), 60.7 (s, *C*(CH_3)$_3$, Bu^t), 60.6 (m, *C*(CH_3)$_3$, Bu^t), 30.1 (s, CH_3 Bu^t), 29.9 (s, CH_3 Bu^t). ^{19}F NMR (376.5 MHz, $CDCl_3$, δ): -105.4 (m, $^4J_{F\text{-}Pt}$ = 50, F^{10}), -107.2 (m, $^4J_{F\text{-}Pt}$ = 40, F^8).

Preparation of [Pt(ppy-CHO)(CNBu^t)$_2$]ClO$_4$ (3b). This compound was prepared as a pale yellow solid (0.330 g, 83 %) following the procedure described for **3a** using a yellow suspension of [Pt(ppy-CHO)(Hppy-CHO)Cl] (0.367 g, 0.616 mmol) in CH_2Cl_2 (10 mL)/acetone (10 mL) with an excess of $KClO_4$ (0.853 g, 6.16 mmol). IR (cm^{-1}): ν(C≡N) 2223 (vs), ν(C≡N) 2248 (vs); ν(ClO_4^-) 1085 (vs), 624 (s). ESI(+): *m/z* (%): 543 $[M-ClO_4]^+$ (63). Anal. Calcd for $C_{22}H_{26}ClN_3O_5Pt$ (643.00): C, 41.10; H, 4.08; N, 5.51. Found: C, 41.05; H, 3.95; N, 5.06. ^1H NMR (400 MHz, CD_2Cl_2, δ): 10.01 (s, CHO), 8.78 (d, $J_{H\text{-}H}$ = 5.3, $^3J_{Pt\text{-}H}$ = 41, H^2), 8.19 (t, $J_{H\text{-}H}$ = 7.6, H^4), 8.06 (s, $^3J_{Pt\text{-}H}$ = 53, H^{11}), 8.03 (d, $J_{H\text{-}H}$ = 8.4, H^5), 7.87 (d, $J_{H\text{-}H}$ = 8.0, H^9), 7.77 (dd, $J_{H\text{-}H}$ = 7.9, H^8), 7.63 (t, $J_{H\text{-}H}$ = 6.1, H^3), 1.77 (s, 9H, CH_3, Bu^t), 1.73 (s, 9H, CH_3, Bu^t). $^{13}C\{^1H\}$ NMR (100.6 MHz, CD_2Cl_2, δ): 191.7 (s, $CHO_{ppy\text{-}CHO}$), 165.3 (s, $C^6_{ppy\text{-}CHO}$), 153.5 (s, $C^2_{ppy\text{-}CHO}$), 152.2 (s, $C^7_{ppy\text{-}CHO}$), 149.2 (s, $C^{12}_{ppy\text{-}CHO}$), 142.2 (s, $C^4_{ppy\text{-}CHO}$), 137.5 (s, $C^{10}_{ppy\text{-}CHO}$), 136.2 (s, $C^{11}_{ppy\text{-}CHO}$), 129.4 (s, $C^9_{ppy\text{-}CHO}$), 126.5 (s, $C^3_{ppy\text{-}CHO}$), 124.9 (s, $C^8_{ppy\text{-}CHO}$), 121.5 (s, $C^5_{ppy\text{-}CHO}$), 60.8 (s, *C*(CH_3)$_3$, Bu^t), 60.5 (s, *C*(CH_3)$_3$, Bu^t), 29.8 (s, CH_3 Bu^t), 29.7 (s, CH_3 Bu^t).

X-ray Crystallography. Details of the X-ray analyses are summarized in Tables S1-S5. Yellow (**1a** and **1b**), red (**1b·0.5Toluene** and **1b·0.5Fluorobenzene (PhF)**) and pale

yellow (**3a·0.25CH$_2$Cl$_2$**) crystals were obtained by slow diffusion of *n*-hexane into solutions of the complexes in CH_2Cl_2 (**1a, 3a·0.25CH$_2$Cl$_2$**), THF (**1b**), toluene (**1b·0.5Toluene**) and fluorobenzene (**1b·0.5PhF**) at room temperature. Slow evaporation of a $CHCl_3$ solution of **1a·CHCl$_3$** (298 K) gave pale-yellow crystals. The diffraction data were collected using graphite-monochromatic Mo-K_α radiation with a Bruker APEX-II diffractometer at 298 K (**1a**, **1a·CHCl$_3$** and **3a·0.25CH$_2$Cl$_2$**) and 100 K (**1b**, **1b·0.5Toluene** and **1b·0.5PhF**) using the APEX-II software. The structures were solved with the WINGX program suite[5] by intrinsic phasing using SHELXT program[6] and refined by full-matrix least squares on F^2 with SHELXL.[7] All non-hydrogen atoms were assigned anisotropic displacement parameters. All the hydrogen atoms were constrained to idealized geometries and assigned isotropic displacement parameters equal to 1.2 times the U_{iso} value of their respective attached carbon for the aromatic and CH_2 hydrogen atoms, except those of methyl groups, which were fixed to 1.5 times the U_{iso} value of their attached carbons. For **1a·CHCl$_3$, 1b·0.5Toluene**, **1b·0.5PhF** and **3a·0.25CH$_2$Cl$_2$**, one $CHCl_3$ molecule, a half toluene and fluorobenzene molecule and a 0.25 dichloromethane molecule, respectively, were properly resolved from the difference density map. During the refinement, several restraints and constraints had to be applied. DFIX, FLAT, EADP, RIGU and TWIN/BASF instructions were employed to model the molecules' geometry and temperature parameters. For **1a**, the planarity of the disordered phenyl ring fragment was reached with FLAT and EADP. For **1a·CHCl$_3$**, the tertbutyl group was modelled as a rotational disorder over two positions in 50:50 ratios. The **3a·0.25CH$_2$Cl$_2$** data included instructions DFIX restraining two C-C bonds of two tertbutyl and three Cl-O bonds in perchlorate anions to have equal lengths. Moreover, four tertbutyl moieties EADP constrains were applied to chemically equivalent atoms. One of the perchlorate anion (ClO_4) was obtained as a distorted fragment and RIGU restraints was applied to the U_{if} coefficients of this anion. In **1a**, **1b·0.5Toluene** and **1b·0.5PhF**, the twinning was treated with the appropriate TWIN law and BASF parameter to refine the twin components due to a small but non-zero Flack parameter.

Computational details. Calculations were carried out with the Gaussian 16 package[8] for compounds **1a**, **1b** and **3a**, using Becke´s three-parameter functional combined with Lee-Yang-Parr´s correlation functional (B3LYP).[9] Optimizations on the singlet state (S_0) were performed using as a starting point the molecular geometry obtained through X-ray diffraction analysis. No negative frequency was found in the vibrational frequency

analysis of the final equilibrium geometries. The basis set used was the LanL2DZ effective core potential for Pt and 6-31G(d,p) for the ligand atoms.[10] DFT and TD-DFT calculations were carried out using the polarized continuum model approach[11] (PCM) implemented in the Gaussian 16 software, in the presence of dichloromethane. To study the packing interaction, the dimeric, trimeric and tetrameric **1a** and **1b** geometries in the ground (S_0) and the first triplet excited (T_1) were optimized in gas phase based on **1a** and **1b·0.5Toluene** crystals structures by using a pair of symmetry-related platinum(II) moieties (dimers), trimers and tetramers with the shortest intermolecular Pt···Pt distance. In **1b·0.5Toluene,** as the solvated molecules of toluene were found to exert insignificant influence on the calculated results, the calculations were performed without considering the toluene molecules. The calculations were carried out through the Grimme approach using atom pair-wise additive schemes using dispersion-corrected B3LYP-D3 method to elucidate the dispersion effects for non-bonding interaction.[12] The emission energy was calculated as the difference of the optimized T_1 and S_0 state in the optimized T_1 geometry (adiabatic electronic transition). The results were visualized with GaussView 6. Overlap populations between molecular fragments were calculated using the GaussSum 3.0 software.[13]

2.- Characterization of complexes

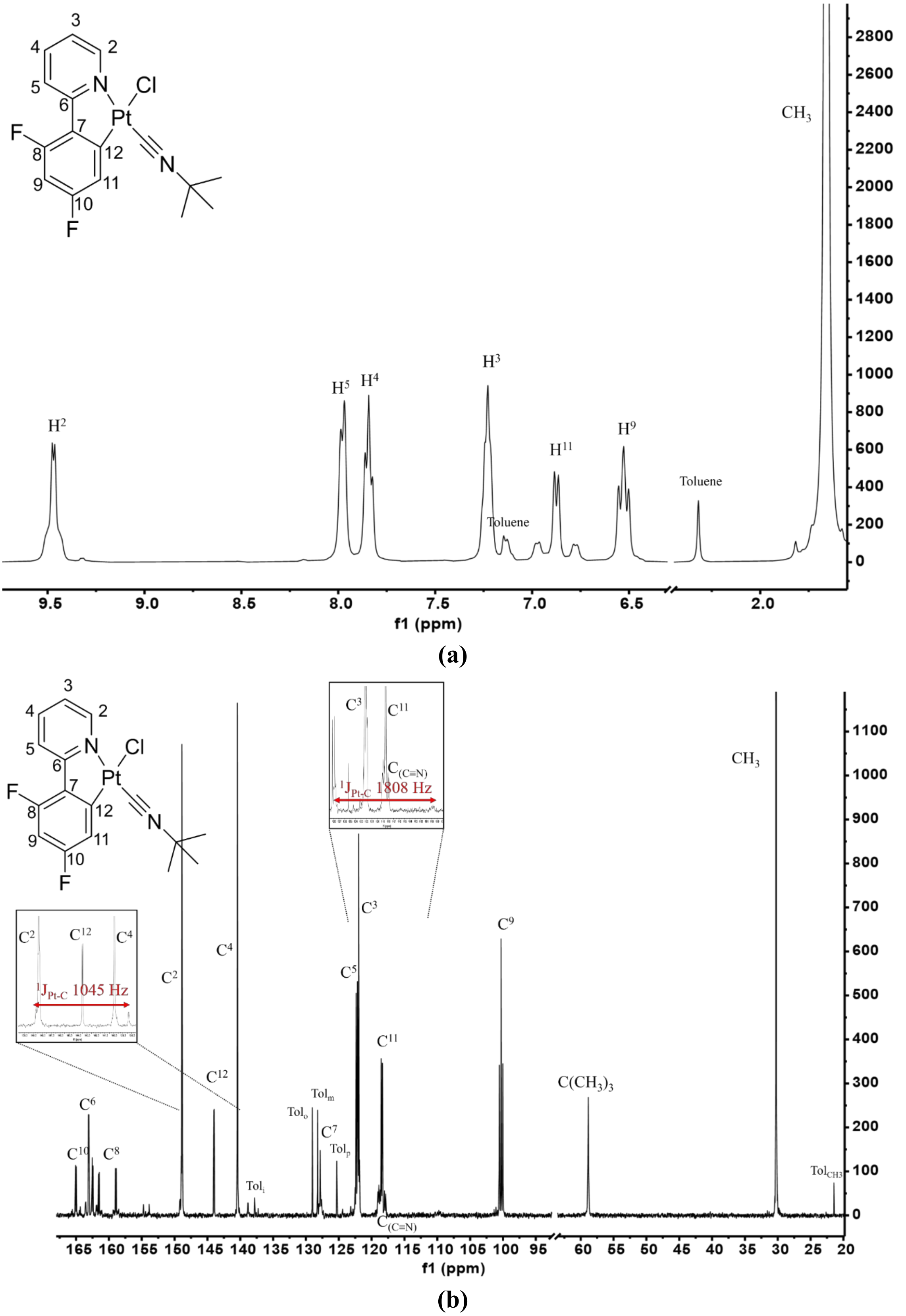

Figure S1. NMR spectra of **1a** in $CDCl_3$ at 298 K, (a) ^1H, (b) $^{13}C\{^1H\}$.

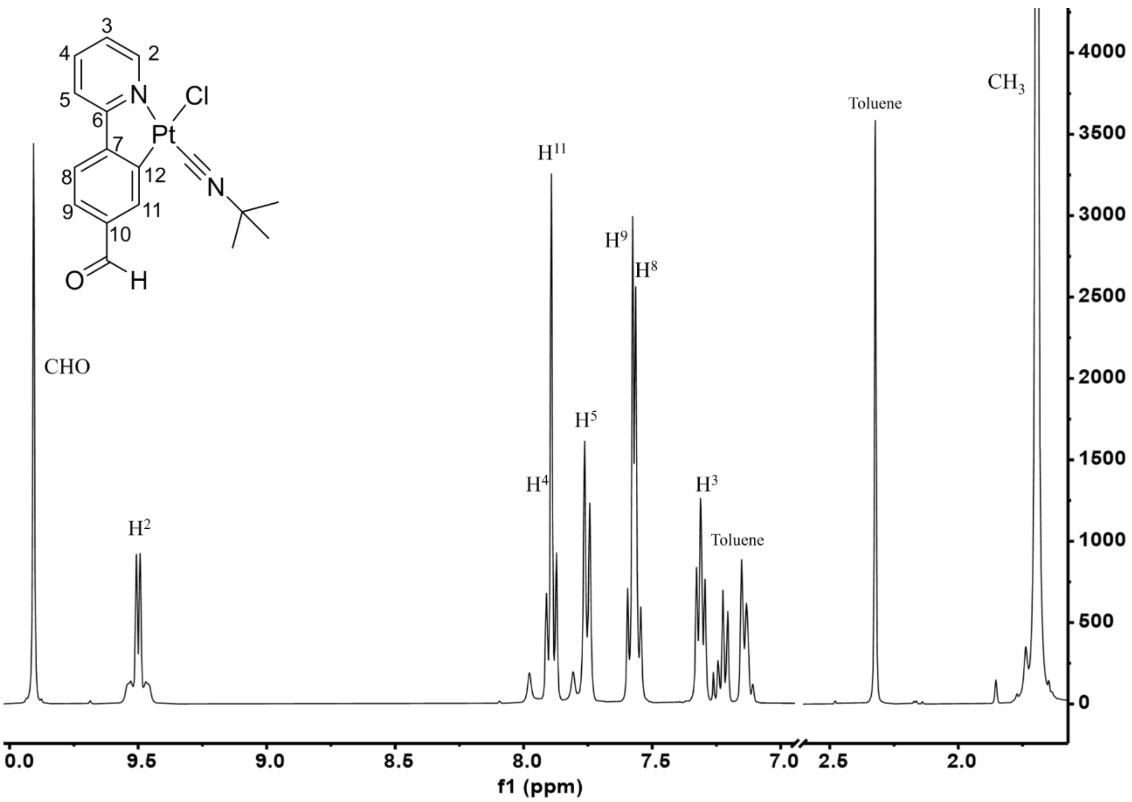

(a)

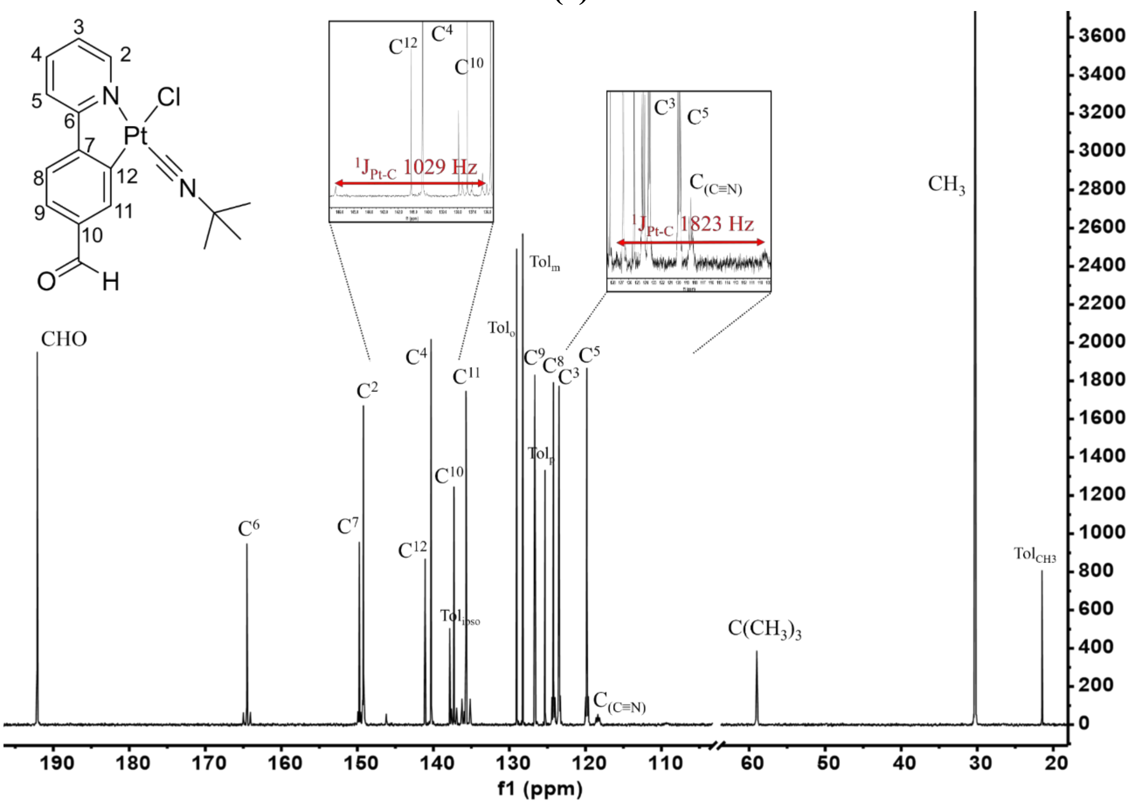

(b)

Figure S2. NMR spectra of **1b** in $CDCl_3$ (0.4 M) at 298 K, (a) ^1H, (b) $^{13}C\{^1H\}$.

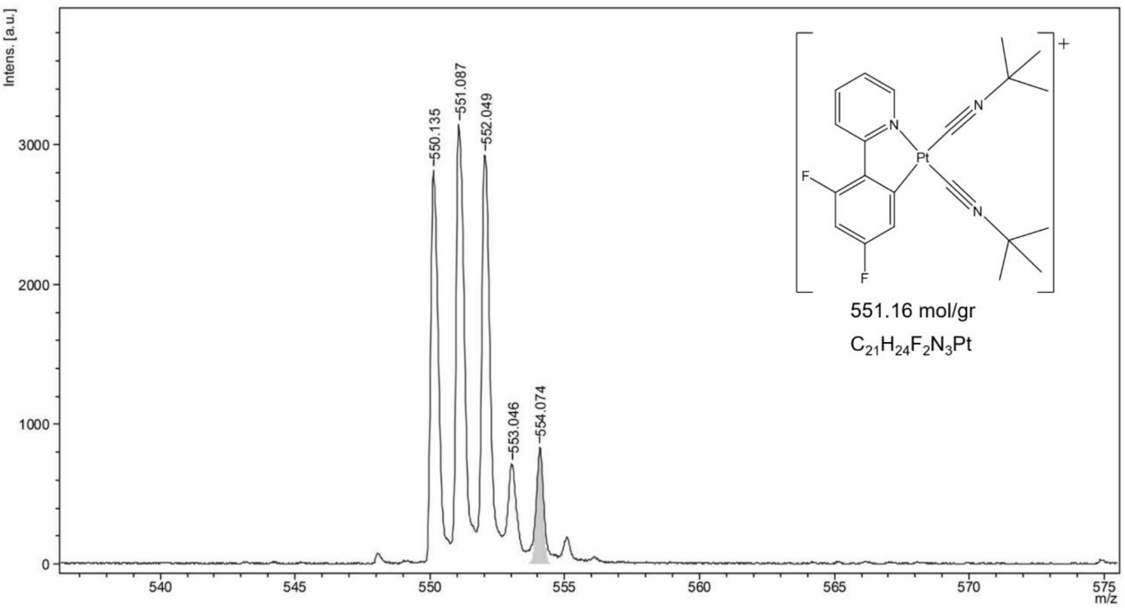

(a)

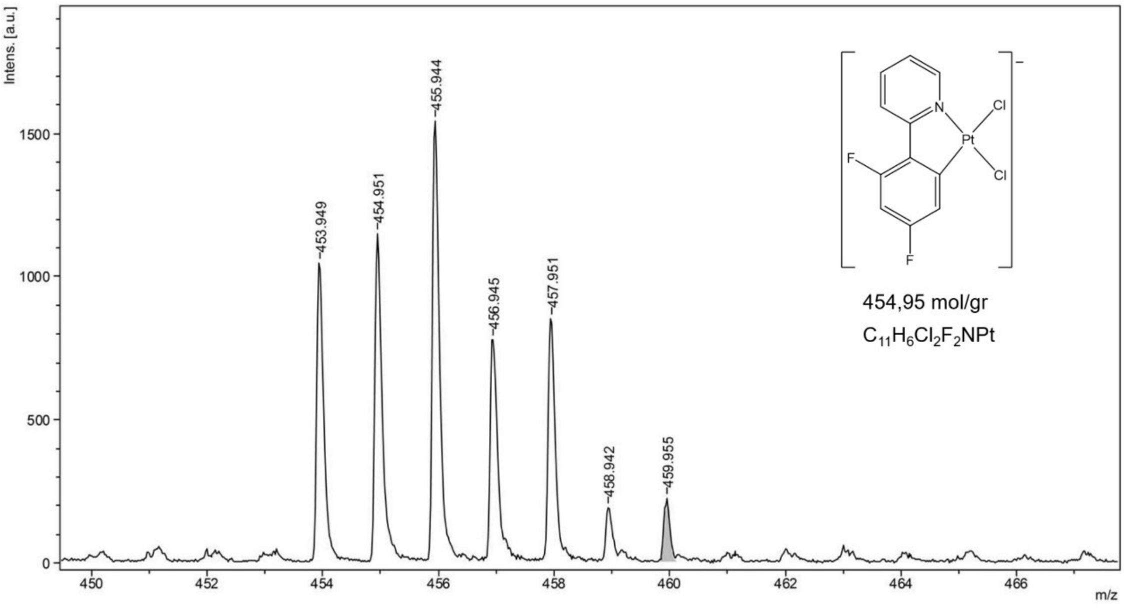

(b)

Figure S3.- MALDI-TOF spectra of **2a** a) in the positive mode and b) in the negative mode

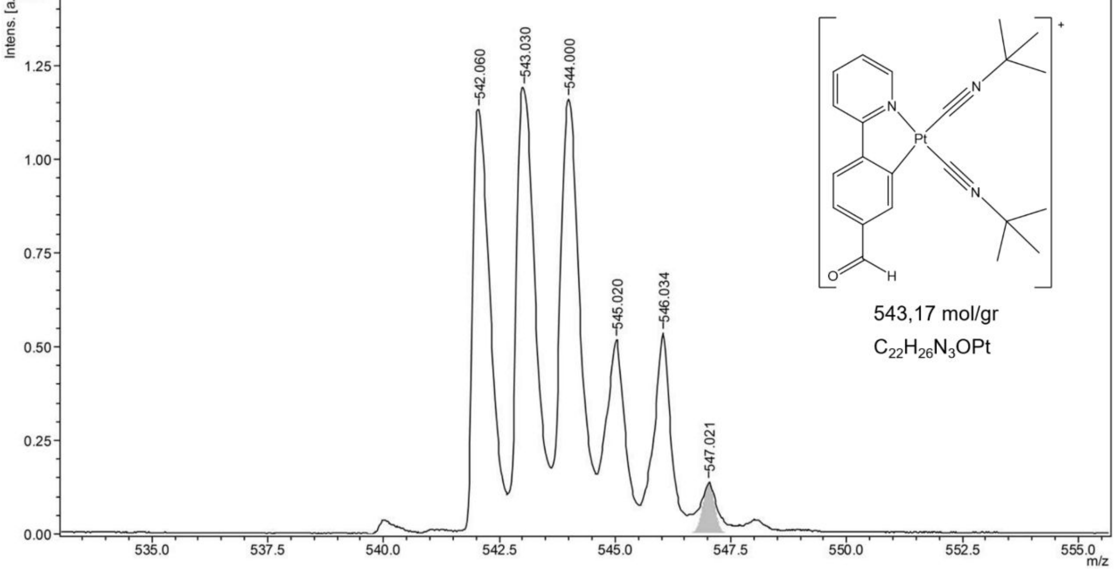

(a)

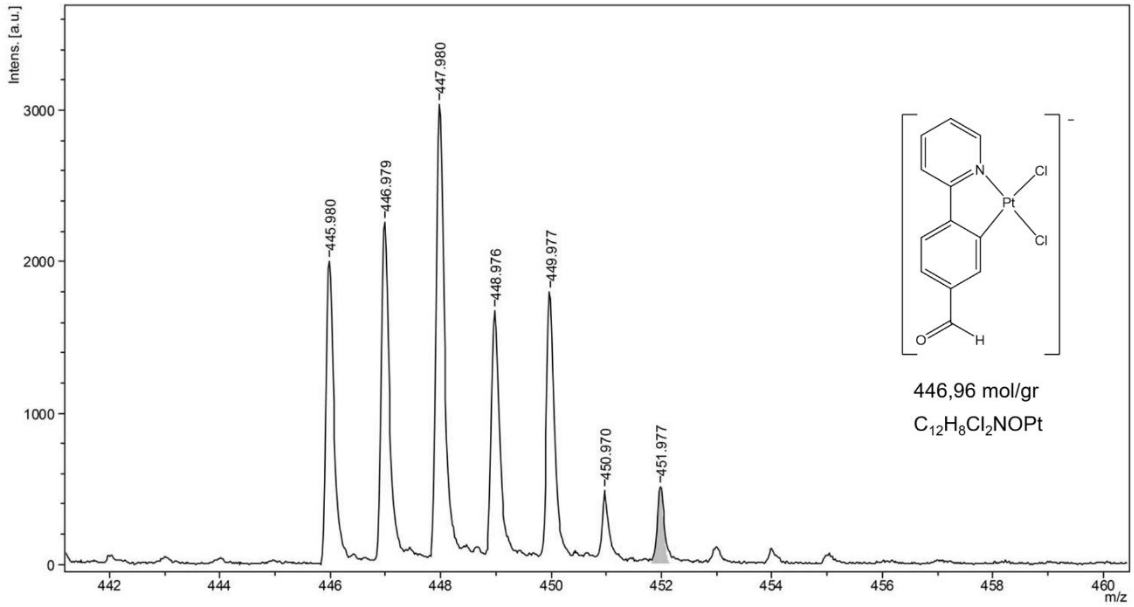

(b)

Figure S4.- MALDI-TOF spectra of **2b** a) in the positive mode and b) in the negative mode

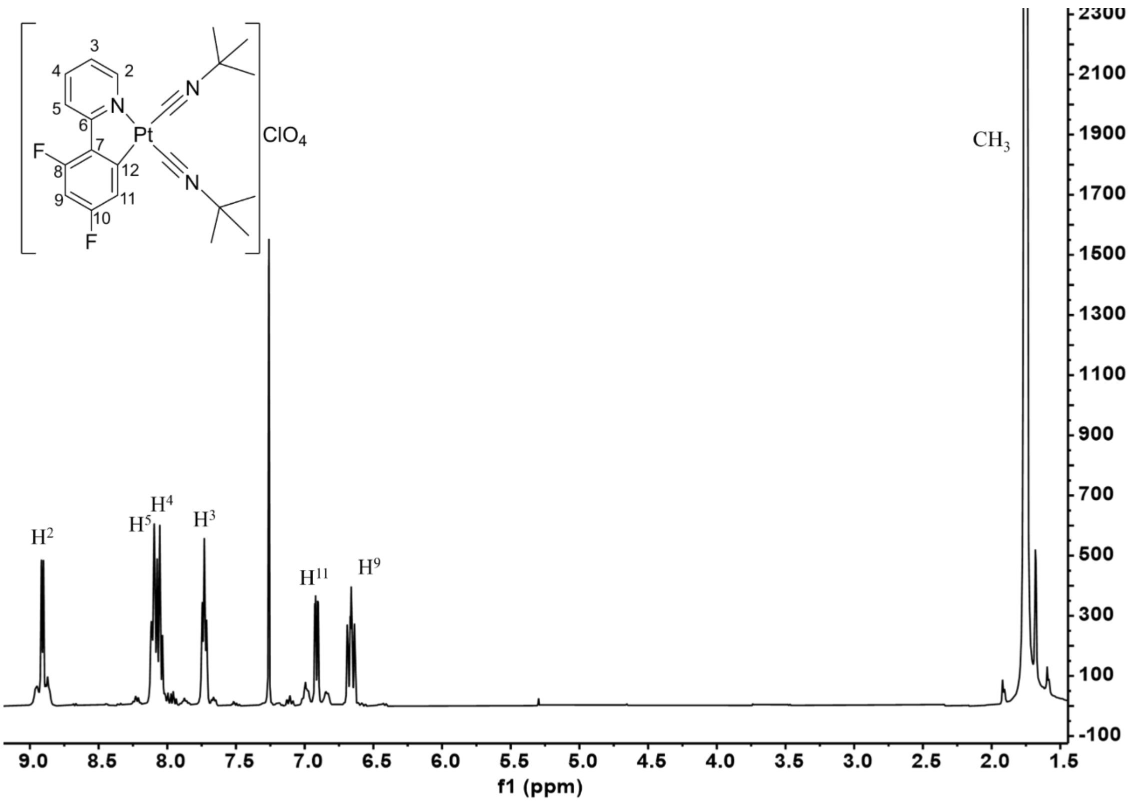

(a)

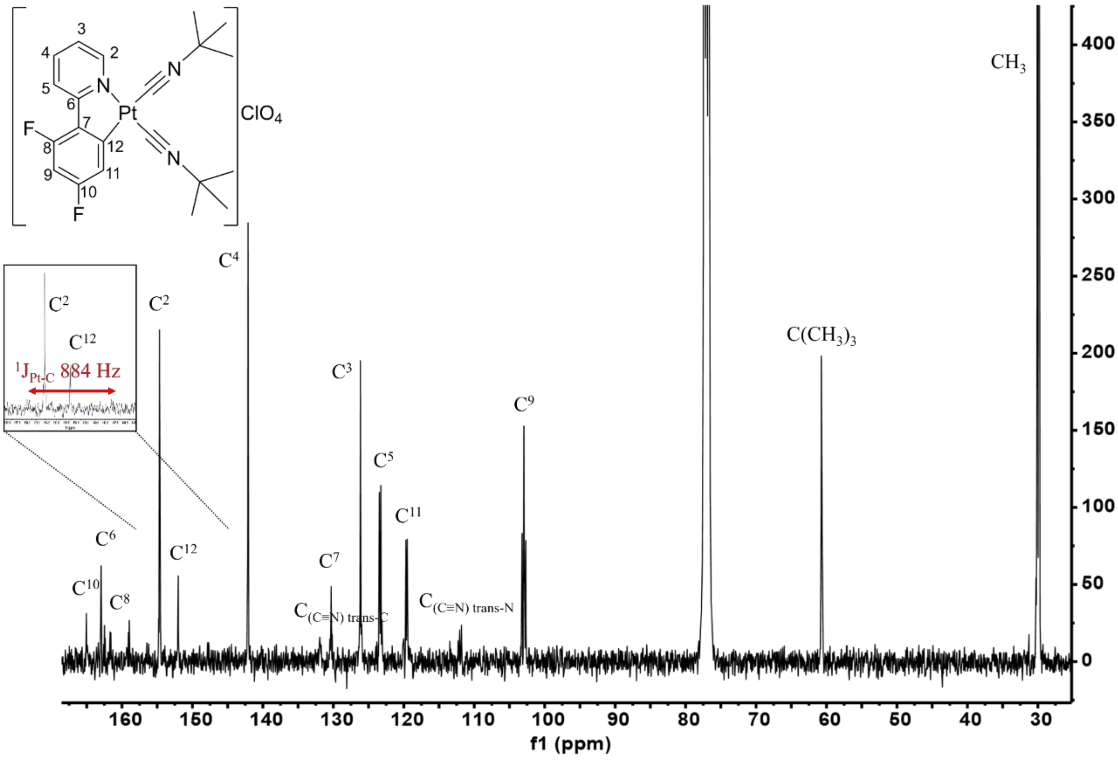

(b)

Figure S5. NMR spectra of **3a** in $CDCl_3$ at 298 K, (a) ^{1}H, (b) $^{13}C\{^{1}H\}$.

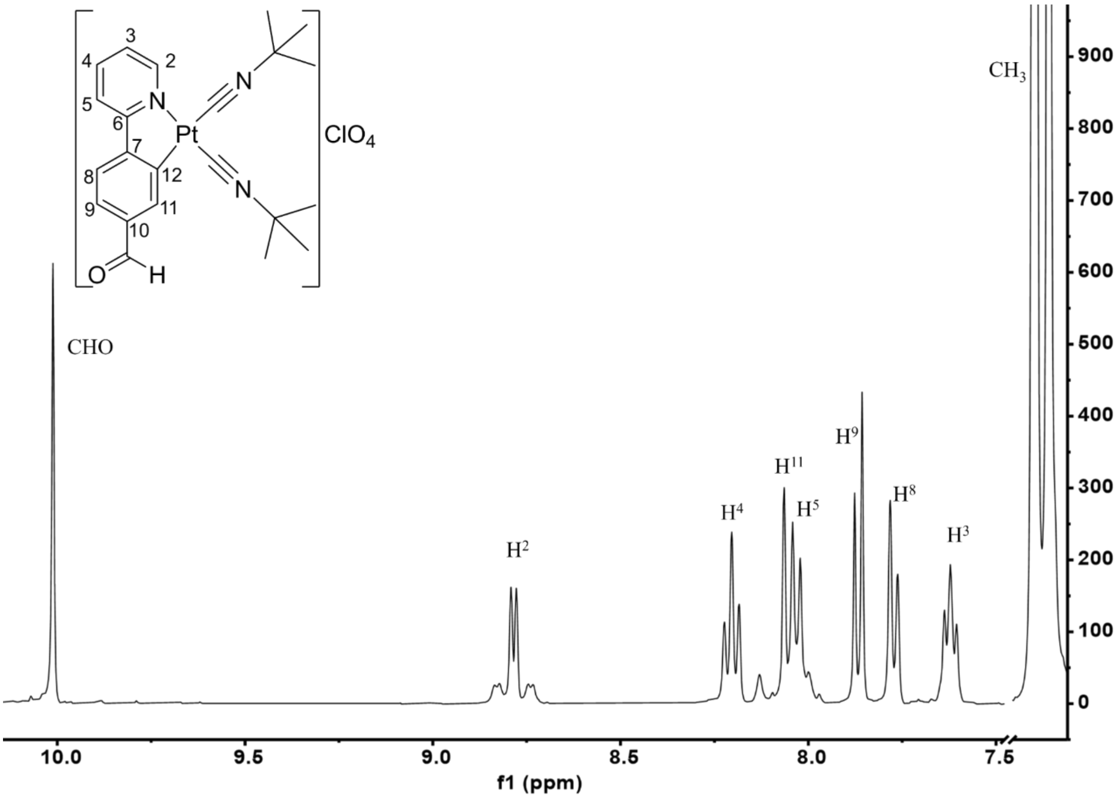

(a)

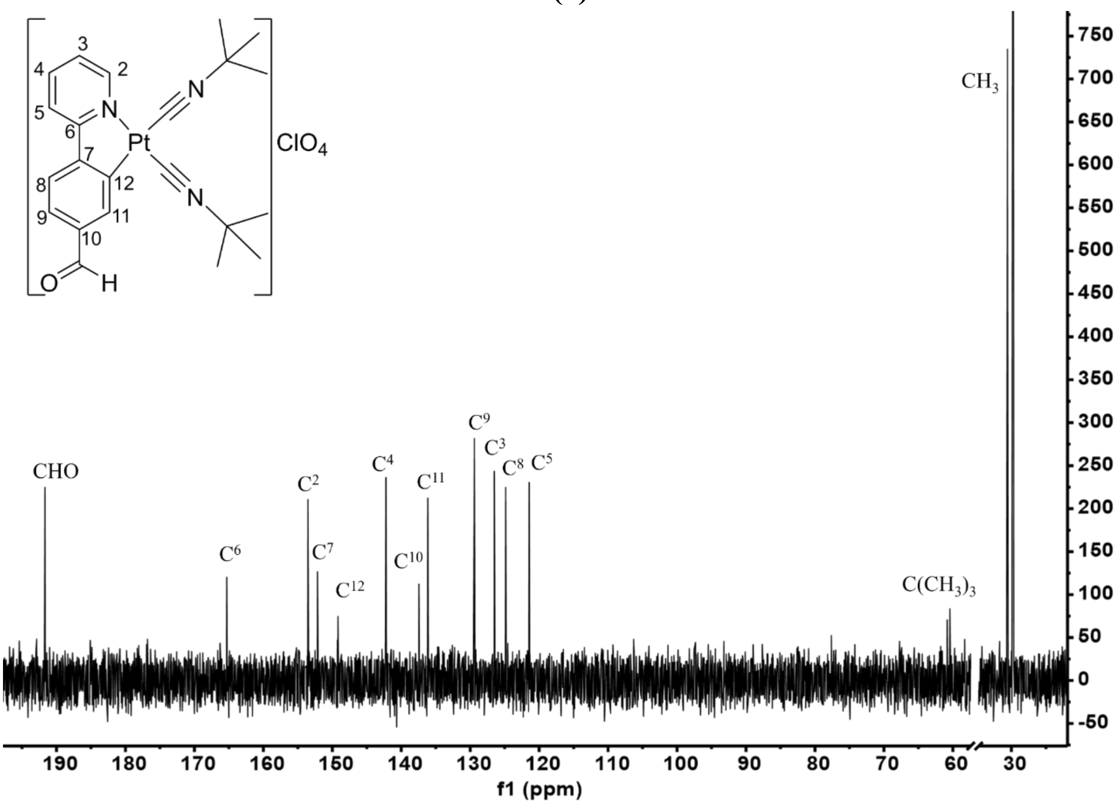

(b)

Figure S6. NMR spectra of **3b** in CD_2Cl_2 at 298 K, (a) ^{1}H, (b) $^{13}C\{^{1}H\}$.

3.- Crystal Structures

Table S1. X-ray Crystallographic Data for **1a** and **1a·CHCl$_3$**.

	**1a**	**1a·CHCl$_3$**
**Empirical formula**	C_{16} H_{15} Cl F_2 N_2 Pt	C_{17} H_{16} C_{l4} F_2 N_2 Pt
**F_w**	503.84	623.21
**T (K)**	298(2)	293(2)
**Wavelength (Å)**	0.71076	0.71076
**Crystal system**	Monoclinic	Monoclinic
**Space group**	P 21/c	P 21/n
**Crystal size (mm^3)**	0.31 x 0.10 x 0.04	0.35 x 0.09 x 0.09
**a (Å)**	7.1648(6)	7.1424(2)
**b (Å)**	17.3048(12)	17.0802(5)
**c (Å)**	26.219(2)	17.3858(6)
**α (°)**	90	90
**β (°)**	90.408(3)	95.8610(10)
**γ (°)**	90	90
**V (Å^3)**	3250.7(4)	2109.87(11)
**Z**	8	4
**D_{calcd} (Mg/m^3)**	2.059	1.962
**Absorption coefficient (mm^{-1})**	8.814	7.178
**F(000)**	1904	1184
**θ range for data collection (deg)**	2.821 to 27.878	2.986 to 27.929
**Index ranges**	-9<=h<=9, -22<=k<=22, -34<=l<=34	-9<=h<=9, -22<=k<=22, -22<=l<=22
**Reflections collected**	207979	111603
**Independent reflections**	7771 [R(int) = 0.0554]	5038 [R(int) = 0.0424]
**Data / restraints/ parameters**	7771 / 3 / 356	5038 / 0 / 262
**Goodness-of-fit on $F^{2\,a}$**	1.157	1.052
**Final R indices [I > 2σ(I)]^a**	R_1 = 0.0332 wR$_2$ = 0.0666	R_1 = 0.0235 wR$_2$ = 0.0598
**R indices (all data)^a**	R_1 = 0.0377, wR$_2$ = 0.0694	R_1 = 0.0320, wR$_2$ = 0.0681
**Largest diff. peak and hole (e. Å^{-3})**	1.481 and -1.509	0.690 and -0.981

[a] $R_1 = \sum(|F_o| - |F_c|)/\sum|F_o|$; $wR_2 = [\sum w(F_o{}^2 - F_c{}^2)^2/\sum wF_o{}^2]^{1/2}$; goodness of fit = $\{\sum[w(F_o{}^2 - F_c{}^2)^2]/(N_{obs} - N_{param})\}^{1/2}$;$w=[\sigma^2(F_o)+(g_1P)^2 + g_2P]^{-1}$; $P = [max(F_o{}^2 ;0 + 2F_c{}^2)/3$.

Table S2. X-ray Crystallographic Data for **1b, 1b·0.5 Toluene** and **1b·0.5 PhF**.

	**1b**	**1b·0.5 Toluene**	**1b·0.5 PhF**
**Empirical formula**	C_{17} H_{17} Cl N_2 O Pt	$C_{20.50}$ H_{21} Cl N_2 O Pt	C20 H19.50 Cl F0.50 N2 O Pt
**F_w**	495.86	541.93	543.91
**T (K)**	100(2)	100(2) K	100(2) K
**Wavelength (Å)**	0.71075	0.71076	0.71076
**Crystal system**	Orthorhombic	Monoclinic	Monoclinic
**Space group**	P n m a	P 21	P 21
**Crystal size (mm^3)**	0.28 x 0.08 x 0.05	0.17 x 0.13 x 0.05	0.24 x 0.06 x 0.04
**a (Å)**	16.347(2)	6.9560(8)	6.9231(5)
**b (Å)**	6.8397(9)	17.430(2)	17.1451(13)
**c (Å)**	14.4809(19)	15.5724(19)	15.6681(11)
**α (°)**	90	90	90
**β (°)**	90	92.380(4)	92.561(3)
**γ (°)**	90	90	90
**V ($Å^3$)**	1619.1(4)	1886.4(4)	1857.9(2)
**Z**	4	4	4
**D_{calcd} (Mg/m^3)**	2.034	1.908	1.945
**Absorption coefficient (mm^{-1})**	8.834	7.591	7.711
**F(000)**	944	1044	1044
**θ range for data collection (deg)**	3.294 to 28.076	2.931 to 25.683	3.167 to 27.925
**Index ranges**	-21<=h<=21, -9<=k<=9, -19<=l<=19	-8<=h<=8, -21<=k<=21, -18<=l<=18	-9<=h<=9, -22<=k<=22, -20<=l<=20
**Reflections collected**	82601	56454	104182
**Independent reflections**	2107 [R(int) = 0.0314]	7095 [R(int) = 0.0255]	8827 [R(int) = 0.0250]
**Data / restraints/ parameters**	2107 / 0 / 130	7095 / 1 / 461	8827 / 1 / 465
**Goodness-of-fit on F^2 [a]**	1.121	1.071	1.079
**Final R indices [I > 2σ(I)][a]**	R_1 = 0.0146<br>wR_2 = 0.0366	R_1 = 0.0101<br>wR_2 = 0.0246	R_1 = 0.0110<br>wR_2 = 0.0294
**R indices (all data)[a]**	R_1 = 0.0189, wR_2 = 0.0372	R_1 = 0.0102, wR_2 = 0.0247	R_1 = 0.0111, wR_2 = 0.0295
**Largest diff. peak and hole (e. $Å^{-3}$)**	0.979 and -1.737	0.528 and -0.559	1.910 and -0.606

[a] $R_1 = \sum(|F_o| - |F_c|)/\sum|F_o|$; $wR_2 = [\sum w(F_o{}^2 - F_c{}^2)^2/\sum wF_o{}^2]^{1/2}$; goodness of fit = $\{\sum[w(F_o{}^2 - F_c{}^2)^2]/(N_{obs} - N_{param})\}^{1/2}$;$w=[\sigma^2(F_o)+(g_1P)^2 + g_2P]^{-1}$; $P = [\max(F_o{}^2 ;0 + 2F_c{}^2)/3$.

Table S3. Selected distances (Å) and angles (°) for complexes **1a**, **1a·$CHCl_3$**, **1b**, **1b·0.5 Toluene** and **1b·0.5 PhF**

**1a (Molecule A)**			
**Distances (Å)**		**Angles (°)**	
Cl(1)-Pt(1)	2.385(2)	N(1)-Pt(1)-Cl(1)	93.43(18)
N(1)-Pt(1)	2.054(5)	C(11)-Pt(1)-N(1)	80.6(2)
C(11)-Pt(1)	1.986(3)	C(12)-Pt(1)-C(11)	94.6(2)
C(12)-Pt(1)	1.921(7)	C(12)-Pt(1)-Cl(1)	91.4(2)
C(12)-N(2)	1.134(8)	N(2)-C(12)-Pt(1)	177.8(7)
C(5)-C(6)	1.459(9)		
**1a (Molecule B)**			
**Distances (Å)**		**Angles (°)**	
Cl(2)-Pt(2)	2.3901(17)	N(3)-Pt(2)-Cl(2)	94.33(16)
N(3)-Pt(2)	2.048(5)	C(27)-Pt(2)-N(3)	81.2(3)
C(27)-Pt(2)	1.993(6)	C(28)-Pt(2)-C(27)	95.2(3)
C(28)-Pt(2)	1.911(6)	C(28)-Pt(2)-Cl(2)	89.2(2)
C(28)-N(4)	1.144(8)	N(4)-C(28)-Pt(2)	176.7(6)
C(22)-C(23)	1.397(10)		
**1a·$CHCl_3$**			
**Distances (Å)**		**Angles (°)**	
Pt(1)-Cl(1)	2.3988(12)	N(1)-Pt(1)-Cl(1)	95.20(10)
Pt(1)-N(1)	2.054(3)	C(11)-Pt(1)-N(1)	80.86(16)
Pt(1)-C(11)	1.986(4)	C(12)-Pt(1)-C(11)	94.19(17)
Pt(1)-C(12)	1.898(4)	C(12)-Pt(1)-Cl(1)	89.75(13)
C(12)-N(2)	1.140(5)	N(2)-C(12)-Pt(1)	179.8(5)
C(5)-C(6)	1.467(6)		
**1b**			
**Distances (Å)**		**Angles (°)**	
Pt(1)-Cl(1)	2.3952(8)	N(1)-Pt(1)-Cl(1)	96.59(8)
Pt(1)-N(1)	2.057(3)	C(12)-Pt(1)-N(1)	80.60(13)
Pt(1)-C(12)	1.992(3)	C(13)-Pt(1)-C(12)	91.81(13)
Pt(1)-C(13)	1.894(3)	C(13)-Pt(1)-Cl(1)	91.00(9)
N(2)-C(13)	1.150(4)	N(2)-C(13)-Pt(1)	175.7(3)
C(5)-C(6)	1.460(5)		
**1b·0.5 Toluene (Molecule A)**			
**Distances (Å)**		**Angles (°)**	
Pt(1)-Cl(1)	2.4054(9)	N(1)-Pt(1)-Cl(1)	94.57(8)
Pt(1)-N(1)	2.057(3)	C(12)-Pt(1)-N(1)	80.54(13)
Pt(1)-C(12)	1.992(3)	C(13)-Pt(1)-C(12)	92.93(14)
Pt(1)-C(13)	1.913(4)	C(13)-Pt(1)-Cl(1)	91.95(10)
N(2)-C(13)	1.148(5)	N(2)-C(13)-Pt(1)	177.3(3)
C(5)-C(6)	1.470(5)		
**1b·0.5 Toluene (Molecule B)**			
**Distances (Å)**		**Angles (°)**	
Pt(2)-Cl(2)	2.3968(9)	N(3)-Pt(2)-Cl(2)	94.15(9)
Pt(2)-N(3)	2.062(3)	C(29)-Pt(2)-N(3)	80.57(13)
Pt(2)-C(29)	1.990(4)	C(30)-Pt(2)-C(29)	92.15(15)
Pt(2)-C(30)	1.909(4)	C(30)-Pt(2)-Cl(2)	93.16(10)
N(4)-C(30)	1.151(5)	N(4)-C(30)-Pt(2)	176.9(3)
C(22)-C(23)	1.468(5)		
**1b·0.5 PhF (Molecule A)**			

Distances (Å)		Angles (°)	
Cl(1)-Pt(1)	2.4001(9)	N(1)-Pt(1)-Cl(1)	94.23(9)
N(1)-Pt(1)	2.061(3)	C(12)-Pt(1)-N(1)	80.88(14)
C(12)-Pt(1)	1.988(4)	C(13)-Pt(1)-C(12)	92.87(15)
C(13)-Pt(1)	1.908(4)	C(13)-Pt(1)-Cl(1)	92.02(11)
C(13)-N(2)	1.152(5)	N(2)-C(13)-Pt(1)	177.6(3)
C(5)-C(6)	1.461(5)		
**1b·0.5 PhF (Molecule B)**			
**Distances (Å)**		**Angles (°)**	
Cl(2)-Pt(2)	2.3935(9)	N(3)-Pt(2)-Cl(2)	94.17(9)
N(3)-Pt(2)	2.062(3)	C(29)-Pt(2)-N(3)	80.78(14)
C(29)-Pt(2)	1.988(4)	C(30)-Pt(2)-C(29)	92.42(15)
C(30)-Pt(2)	1.905(4)	C(30)-Pt(2)-Cl(2)	92.66(11)
C(30)-N(4)	1.153(5)	N(4)-C(30)-Pt(2)	177.1(3)
C(22)-C(23)	1.465(6)		

Table S4. X-ray Crystallographic Data for **3a·0.25CH$_2$Cl$_2$**.

	**3a·0.25CH$_2$Cl$_2$**
**Empirical formula**	$C_{21.25} H_{24.50} Cl_{1.50} F_2 N_3 O_4$ Pt
**F$_w$**	672.20
**T (K)**	298(2) K
**Wavelength (Å)**	0.71073 Å
**Crystal system**	Triclinic
**Space group**	P -1
**Crystal size (mm^3)**	0.170 x 0.160 x 0.140 mm^3
**a (Å)**	14.3923(13)
**b (Å)**	14.4072(11)
**c (Å)**	26.822(2)
**α (°)**	103.518(3)°
**β (°)**	104.516(3)°
**γ (°)**	95.340(3)°
**V (Å^3)**	5166.4(8)
**Z**	8
**D$_{calcd}$ (Mg/m3)**	1.728
**Absorption coefficient (mm^{-1})**	5.631
**F(000)**	2612
**θ range for data collection (deg)**	2.962 to 25.682°
**Index ranges**	-17<=h<=17, -17<=k<=17, -32<=l<=32
**Reflections collected**	238281
**Independent reflections**	19517 [R(int) = 0.0396]
**Data / restraints/ parameters**	19517 / 3 / 1112
**Goodness-of-fit on F$^{2\ a}$**	1.102
**Final R índices [I > 2σ(I)]^a**	R$_1$ = 0.0391<br>wR$_2$ = 0.1045
**R indices (all data)^a**	R$_1$ = 0.0527, wR$_2$ = 0.1171
**Largest diff. peak and hole (e. Å^{-3})**	1.604 and -1.109 e.Å^{-3}

^{a} $R_1 = \sum(|F_o| - |F_c|)/\sum|F_o|$; $wR_2 = [\sum w(F_o{}^2 - F_c{}^2)^2/\sum wF_o{}^2]^{1/2}$; goodness of fit = $\{\sum[w(F_o{}^2 - F_c{}^2)^2]/(N_{obs} - N_{param})\}^{1/2}$;$w=[\sigma^2(F_o)+(g_1P)^2 + g_2P]^{-1}$; $P = [\max(F_o{}^2 ;0 + 2F_c{}^2)/3$.

Table S5. Selected distances (Å) and angles (°) for **3a·0.25CH_2Cl_2**

**3a·0.25CH_2Cl_2 (molecule A)**			
**Distances (Å)**		**Angles (°)**	
Pt(1)-C(12)	1.940(9)	C(12)-Pt(1)-C(17)	88.9(4)
Pt(1)-C(17)	2.011(10)	C(12)-Pt(1)-C(11)	94.6(3)
Pt(1)-C(11)	2.019(7)	C(17)-Pt(1)-N(1)	95.5(3)
Pt(1)-N(1)	2.042(6)	C(11)-Pt(1)-N(1)	81.2(3)
C(12)-N(2)	1.139(10)	N(2)-C(12)-Pt(1)	178.7(8)
C(17)-N(3)	1.133(12)	N(3)-C(17)-Pt(1)	175.5(10)
C(6)-C(5)	1.451(10)		
Pt···Pt	4.56 / 5.16 / 4.71		
$d_{interplanar}^{a}$	3.391 / 3.538		

[a] The shortest interplanar distance

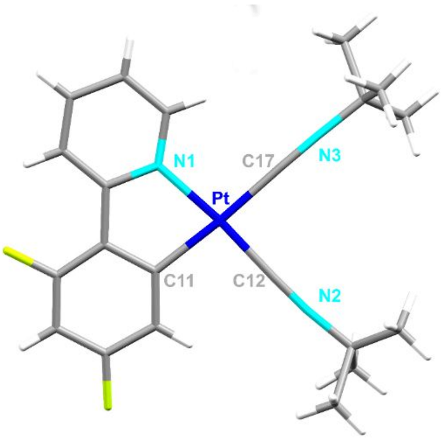

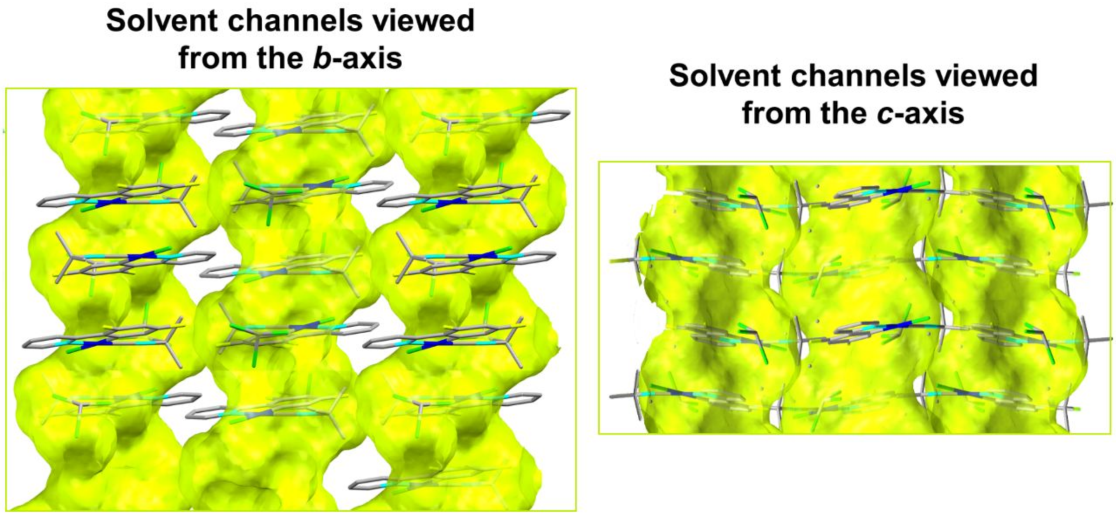

Figure S7. Different views of the crystal packing of **1a·CHCl$_3$** with the solvent channels marked in green. These channels have an aperture size of 11.1 Å x 10.3 Å (estimated from the diagonal atom pairs F2$_{(1/2\text{-x, -1/2+y, 1.5-z})}$···F2$_{(1/2\text{+x, ½-y, -1/2+z})}$ and Cl1$_{(\text{x,y,z})}$···Cl1$_{(1\text{-x,-y,1-z})}$) and the total void volume occupies 26.3% of the unit cell (calculated by the PLATON program).[14]

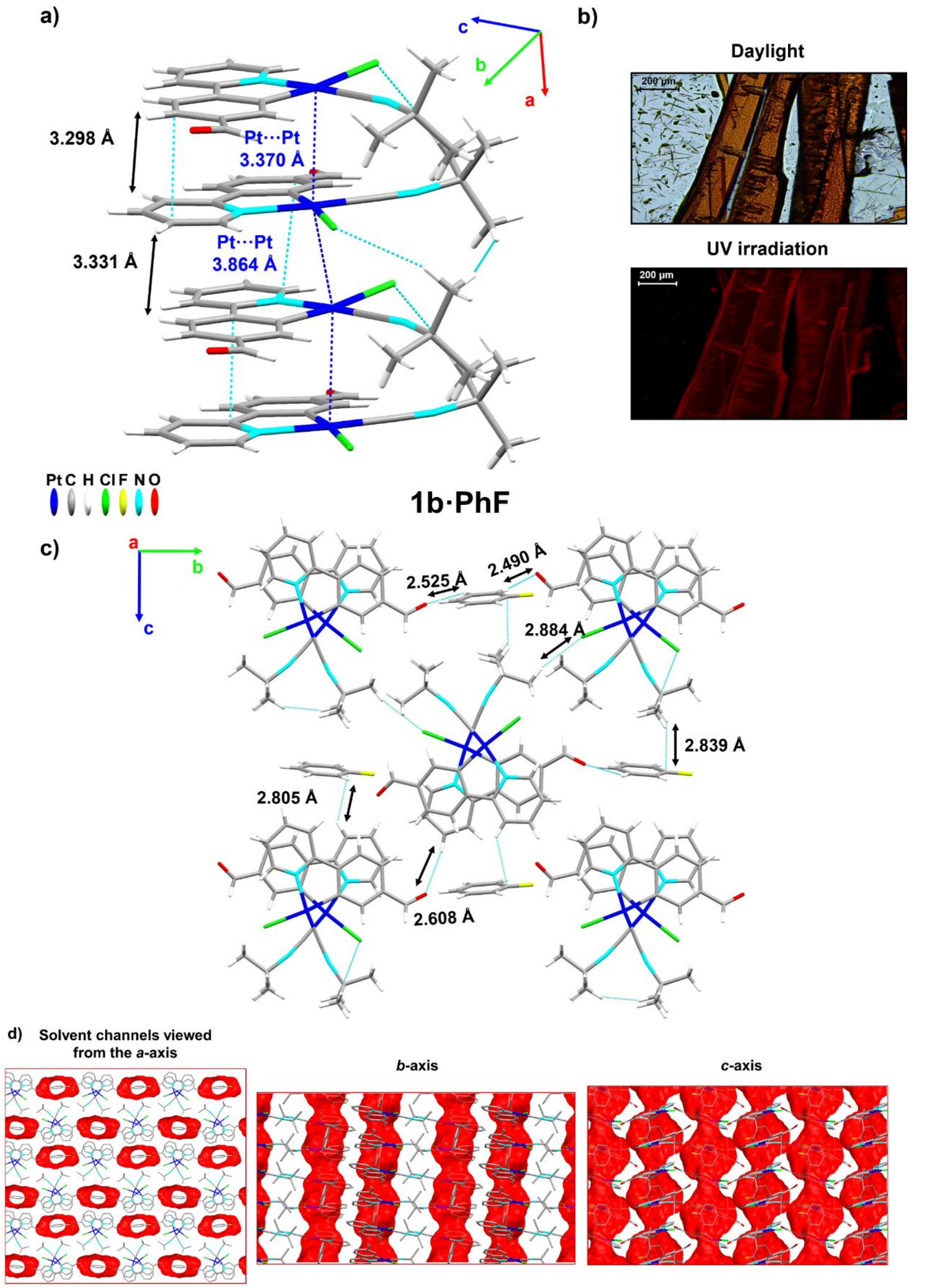

Figure S8. Crystal packing of **1b·0.5 PhF**, a) columnar stacking along the *a* axis showing the π···π interplanar and Pt···Pt distances. b) Pictures of crystals under daylight or UV irradiation. c) Top view from the *a* axis of five stackings showing the toluene solvent localization and secondary contacts. d) View of packing structure with the solvent channels marked in red along the *a, b* and *c* axis, respectively.

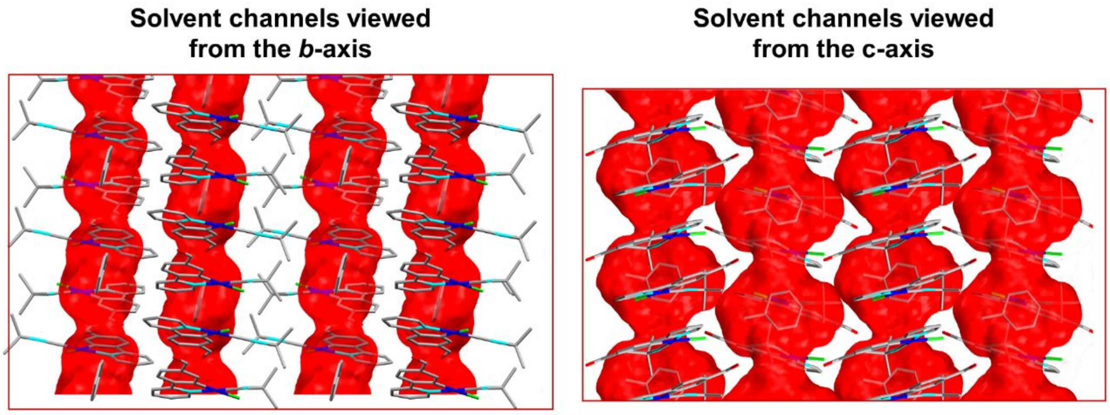

Figure S9. Different views of the crystal packing of **1b·0.5 Toluene** with the solvent channels marked in red. The total solvent-accessible volume occupies 22.7 % of the total volume of the unit cell (or 213.7 Å^3 per toluene molecule) for the **1b·0.5Toluene** structure and 18.1 % for **1b·0.5PhF**.

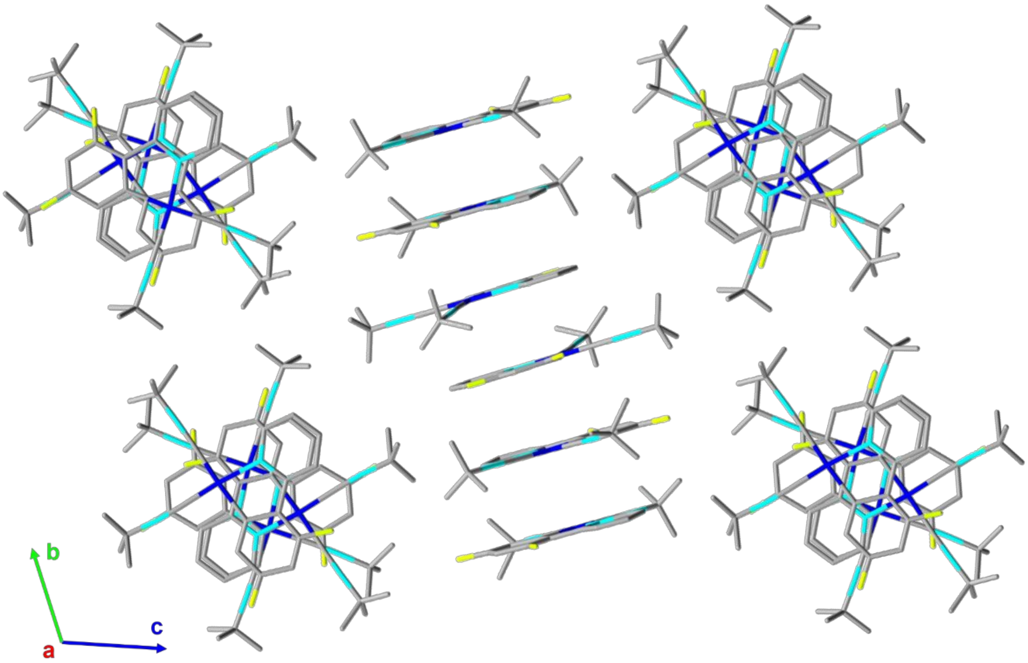

Figure S10. Top view of columns from the *a* axis in the single infinite crystal packing of **3a·0.25CH$_2$Cl$_2$**

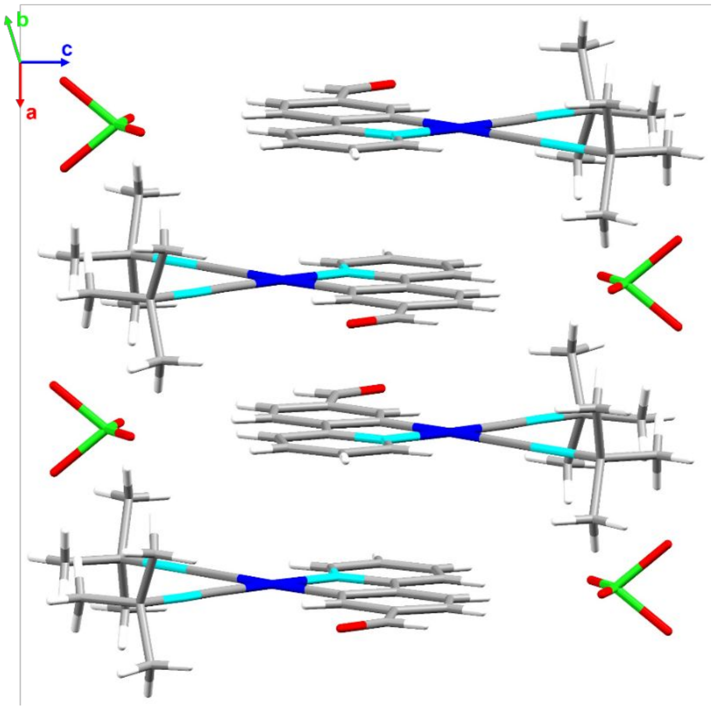

Figure S11. Connectivity and crystal packing of **3b** showing a head-to-head stacking of the cations in a columnar way along the *a* axis.

4.- Photophysical Properties and Theoretical Calculations

Table S6. Absorption data for compounds **1a,b** and **3a,b** (Solutions 5 × 10^{-5} M)

Compound	Media	λ_{abs}/nm (ε x 10^{-3} M^{-1} cm^{-1})
**1a**	CH_2Cl_2	243 (45.9), 267 (22.8), 312 (12.8), 322 (13.9), 362 (4.2), 379 (3.4)
	THF	243 (43.7), 255_{sh} (21.4), 271 (15.8), 311 (9.4), 321 (11.3), 362 (2.9), 382 (2.7)
**1b**	CH_2Cl_2	252 (53.0), 297 (49.1), 322_{sh} (24.1), 335 (20.3), 389 (6.9), 408 (6.3)
	THF	251 (26.6), 301 (22.0), 322_{sh} (11.4), 335 (8.6), 392 (3.1), 412 (3.3)
**3a**	CH_2Cl_2	237 (29.1), 263 (35.8), 309_{sh} (9.4), 325 (8.8), 351 (4.4), 368 (2.7)
**3b**	CH_2Cl_2	239 (25.1), 278 (40.0), 287_{sh} (32.8), 323 (10.8), 337 (12.9), 360 (6.3), 375 (4.7)

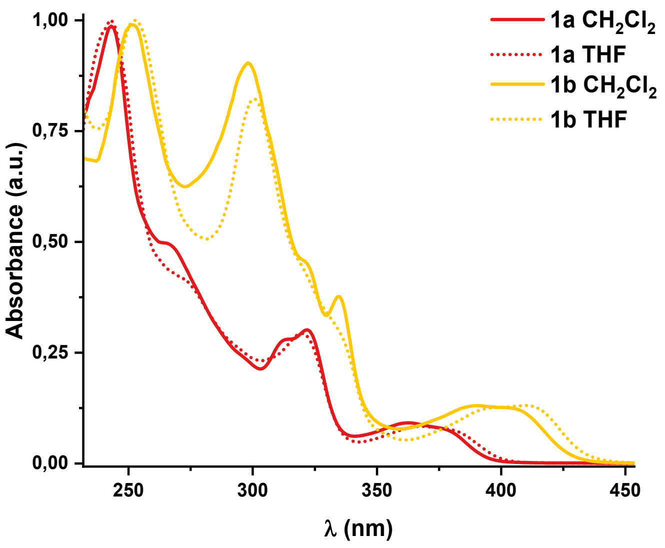

Figure S12. Normalized absorption spectra of complexes **1a** and **1b** in CH_2Cl_2 and THF (5 × 10^{-5} M).

Table S7. DFT optimized geometries for ground state and triplet state of **1a**, **1b** and **3a** in CH_2Cl_2.

1a

	X-ray	S_0	T_1
Cl(1)-Pt(1)	2.3988(12)	2.508	2.508
N(1)-Pt(1)	2.054(3)	2.093	2.060
C(11)-Pt(1)	1.986(4)	2.009	1.981
C(12)-Pt(1)	1.898(4)	1.923	1.934
C(12)-N(2)	1.141(5)	1.166	1.166
C(5)-C(6)	1.466(6)	1.466	1.394
N(1)-Pt(1)-Cl(1)	95.21(10)	95.13	94.62
C(11)-Pt(1)-N(1)	80.85(16)	80.45	81.49
C(12)-Pt(1)-C(11)	94.18(17)	96.12	96.16
C(12)-Pt(1)-Cl(1)	89.75(13)	88.31	87.72
N(2)-C(12)-Pt(1)	179.8(5)	179.1	178.7

1b

	X-ray	S_0	T_1
Pt(1)-Cl(1)	2.3952(8)	2.507	2.494
Pt(1)-N(1)	2.057(3)	2.100	2.079
Pt(1)-C(12)	1.992(3)	2.011	1.970
Pt(1)-C(13)	1.894(3)	1.920	1.937
N(2)-C(13)	1.150(4)	1.166	1.164
C(5)-C(6)	1.460(5)	1.468	1.407
N(1)-Pt(1)-Cl(1)	96.59(8)	94.72	94.25
C(12)-Pt(1)-N(1)	80.60(13)	80.29	81.78
C(13)-Pt(1)-C(12)	91.81(13)	96.19	95.96
C(13)-Pt(1)-Cl(1)	91.00(9)	88.79	88.01
N(2)-C(13)-Pt(1)	175.7(3)	179.0	178.3

3a

	X-ray	S_0	T_1
N(1)-Pt(1)	2.042(6)	2.099	2.066
C(11)-Pt(1)	2.019(7)	2.038	2.015
C(12)-Pt(1)	1.940(9)	1.940	1.944
C(17)-Pt(1)	2.011(10)	2.056	2.050
C(12)-N(2)	1.139(10)	1.163	1.164
C(17)-N(3)	1.133(12)	1.163	1.164
C(5)-C(6)	1.451(10)	1.466	1.393
N(1)-Pt(1)-C(17)	95.5(3)	96.25	96.02
C(11)-Pt(1)-N(1)	81.2(3)	80.18	80.77
C(12)-Pt(1)-C(11)	94.6(3)	93.74	93.66
C(12)-Pt(1)-C(17)	88.9(4)	89.84	89.56
N(2)-C(12)-Pt(1)	178.7(8)	179.54	179.48
N(3)-C(17)-Pt(1)	175.5(10)	178.58	178.63

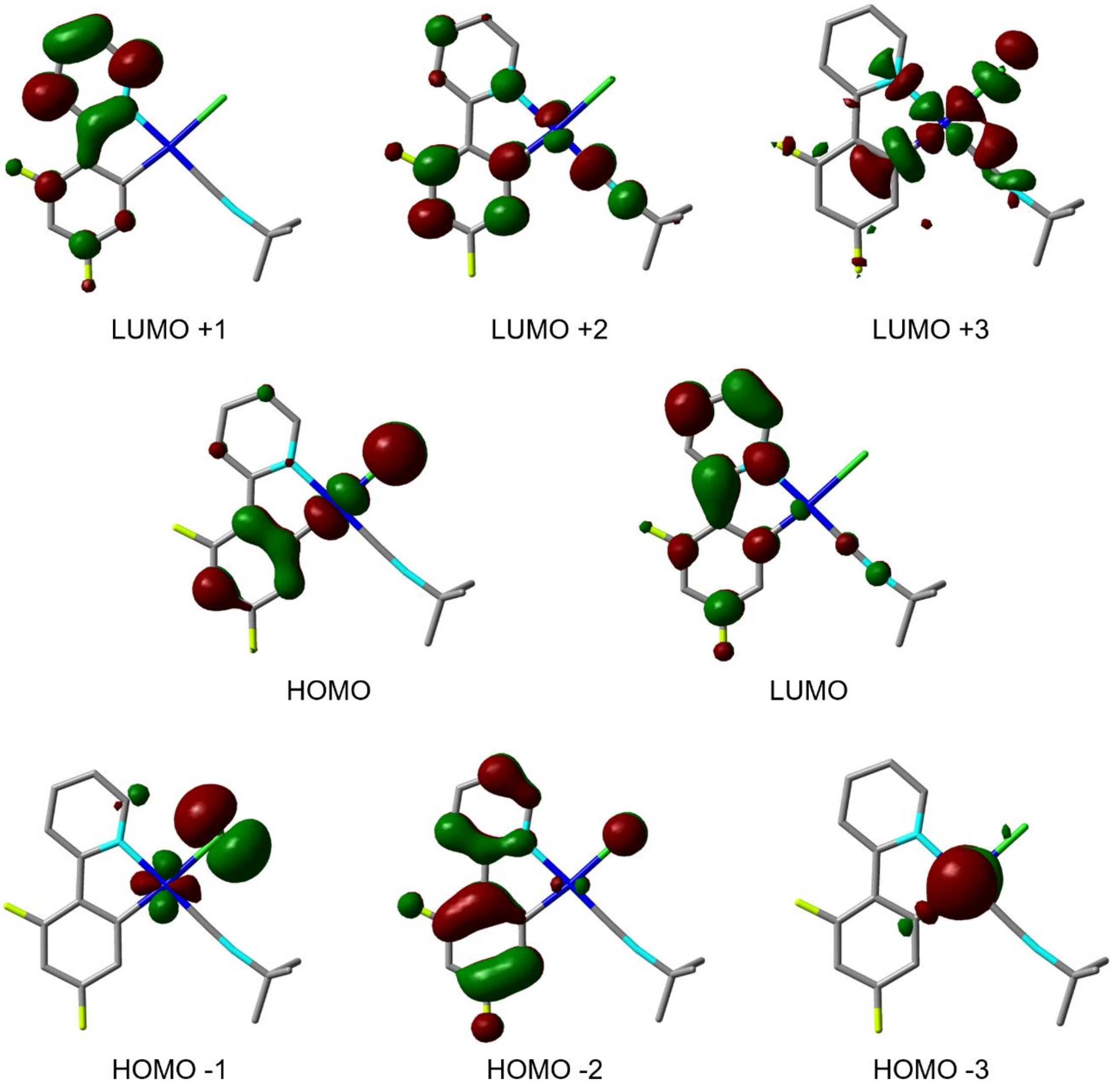

Figure S13. Selected frontier Molecular Orbitals for **1a** in the ground state.

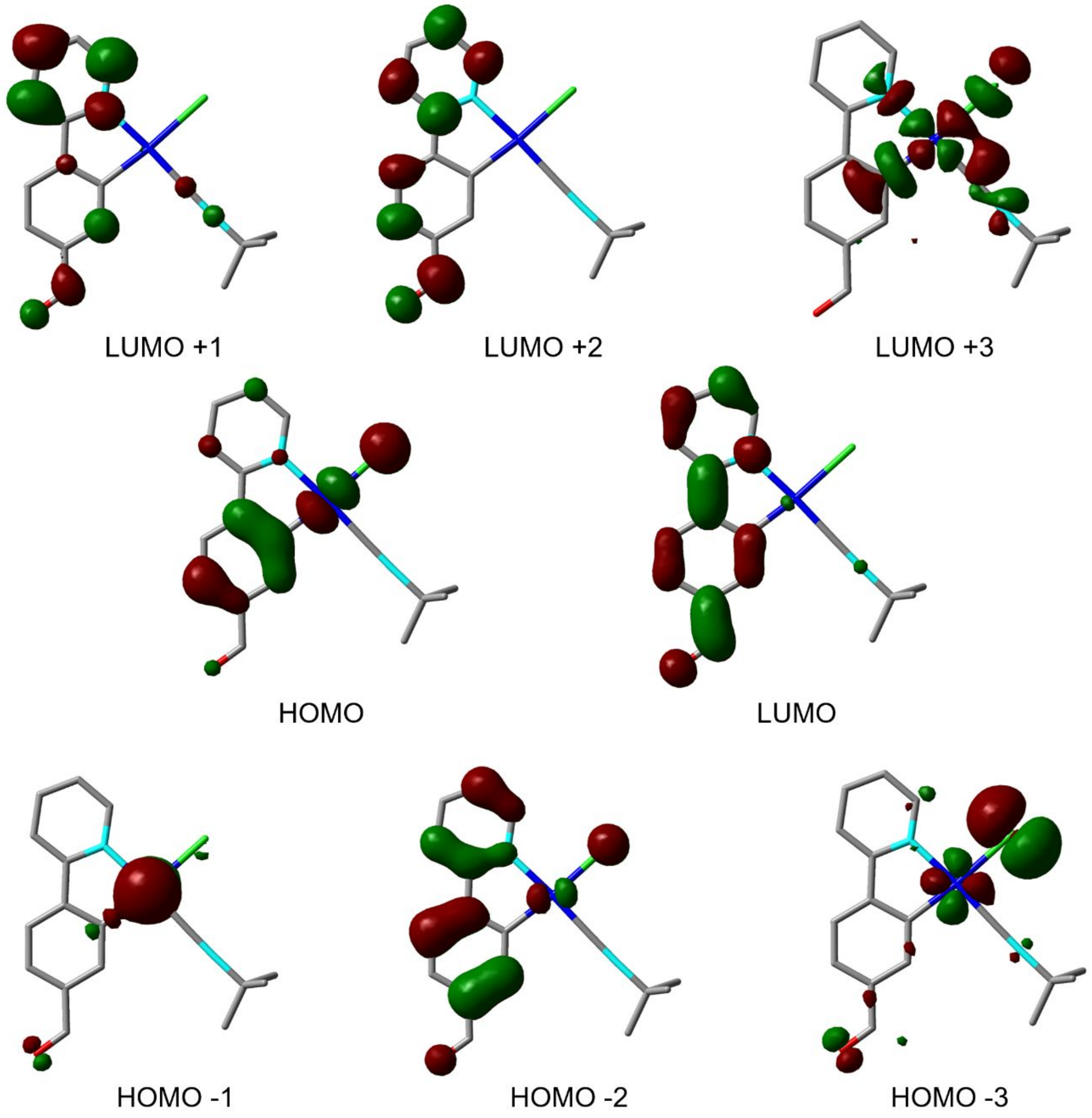

Figure S14. Selected frontier Molecular Orbitals for **1b** in the ground state.

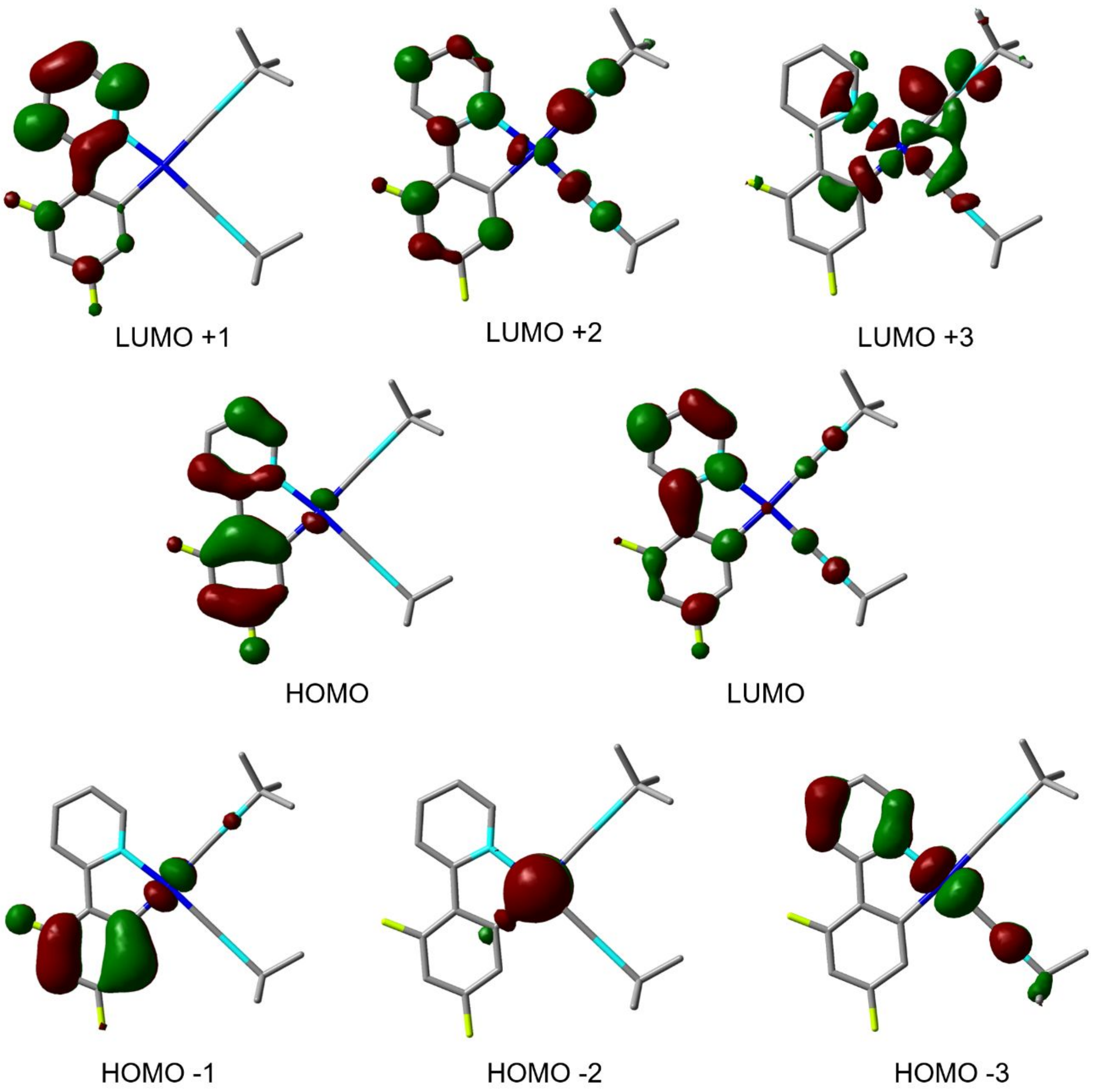

Figure S15. Selected frontier Molecular Orbitals for **3a** in the ground state.

Table S8. Composition (%) of Frontier MOs in terms of ligands and metals in the ground state for **1a**, **1b** and **3a** in CH_2Cl_2.

1a

MO	eV	Pt	dfppy	CNBu^t	Cl
LUMO+5	0.2	17	8	75	0
LUMO+4	0.06	15	52	33	0
LUMO+3	-0.53	24	57	18	1
LUMO+2	-0.65	45	31	15	8
LUMO+1	-1.1	1	98	1	0
LUMO	-1.98	8	86	6	0
HOMO	-6.16	30	55	0	15
HOMO-1	-6.57	7	85	0	7
HOMO-2	-6.78	92	5	1	2
HOMO-3	-6.95	19	4	1	76
HOMO-4	-7.26	25	36	3	35
HOMO-5	-7.37	41	28	12	19

1b

MO	eV	Pt	ppy-CHO	CNBu^t	Cl
LUMO+5	0.05	13	61	26	0
LUMO+4	-0.51	26	53	20	1
LUMO+3	-0.58	46	28	18	8
LUMO+2	-0.73	2	97	1	0
LUMO+1	-1.45	4	91	5	0
LUMO	-2.43	4	94	2	0
HOMO	-6.14	34	49	0	17
HOMO-1	-6.75	89	8	1	1
HOMO-2	-6.84	6	85	0	9
HOMO-3	-6.93	18	10	1	71
HOMO-4	-7.12	5	85	0	9
HOMO-5	-7.29	27	39	4	30

3a

MO	eV	Pt	dfppy	CNBu^t
LUMO+5	-0.38	7	68	25
LUMO+4	-0.45	16	9	76
LUMO+3	-1.11	38	24	38
LUMO+2	-1.38	23	44	33
LUMO+1	-1.51	1	99	1
LUMO	-2.54	13	73	14
HOMO	-6.77	5	94	1
HOMO-1	-7.13	14	83	2
HOMO-2	-7.59	92	8	1
HOMO-3	-8.05	56	30	14
HOMO-4	-8.57	9	90	1
HOMO-5	-8.67	48	39	13

Table S9. Selected vertical excitation energies singlets (S_0) and the first triplet computed by TDDFT/SCRF (CH_2Cl_2) with the orbitals involved for **1a**, **1b** and **3a**.

	State	λ/nm	f	Transition (% Contribution)
**1a**	T_1	436.99	-	H-1->LUMO (19%). HOMO->LUMO (66%)
	T_2	363.04	-	H-1->LUMO (57%). HOMO->LUMO (27%)
	T_3	334.42	-	H-1->LUMO (17%). H-1->L+1 (17%). HOMO->L+1 (37%). H-7->L+1 (9%)
	S_1	359.98	0.0533	HOMO->LUMO (97%)
	S_2	316.84	0.0125	H-2->LUMO (99%)
	S_3	309.22	0.1516	H-1->LUMO (90%)
	S_6	284.02	0.0256	H-4->LUMO (19%). HOMO->L+1 (72%)
	S_7	271.91	0.0633	H-4->LUMO (11%). H-2->L+2 (69%)
	S_8	269.88	0.1623	H-5->LUMO (17%). H-4->LUMO (40%). H-2->L+2 (23%)
	S_9	265.02	0.0267	H-5->LUMO (60%). H-4->LUMO (23%)
	S_{10}	254.28	0.1231	H-5->LUMO (12%). H-1->L+1 (19%). HOMO->L+3 (58%)
	State	λ/nm	f	Transition (% Contribution)
**1b**	T_1	506.99	-	H-2->LUMO (16%). HOMO->LUMO (77%)
	T_2	407.71	-	H-4->LUMO (60%). H-4->L+2 (11%). H-4->L+1 (8%)
	T_3	404.86	-	H-2->LUMO (70%). HOMO->LUMO (17%)
	S_1	401.18	0.0772	HOMO->LUMO (97%)
	S_2	357.88	0.002	H-4->LUMO (27%). H-1->LUMO (58%)
	S_3	346.86	0.0038	H-4->LUMO (44%). H-1->LUMO (41%)
	S_5	318.01	0.1864	H-2->LUMO (67%). HOMO->L+1 (28%)
	S_6	308.89	0.1504	H-5->LUMO (13%). H-2->LUMO (22%). HOMO->L+1 (60%)
	S_7	293.41	0.214	H-6->LUMO (13%). H-5->LUMO (67%)
	S_9	286.14	0.0529	H-6->LUMO (79%). H-5->LUMO (15%)
	State	λ/nm	f	Transition (% Contribution)
**3a**	T_1	441.22	-	HOMO->LUMO (83%)
	T_2	351.75	-	H-1->LUMO (92%)
	T_3	338.38	-	H-4->L+1 (10%), HOMO->L+1 (62%)
	S_1	346.61	0.0797	HOMO->LUMO (92%)
	S_2	315.45	0.1348	H-1->LUMO (89%)
	S_3	296.76	0.0226	H-2->LUMO (99%)
	S_4	269.10	0.0062	H-3->LUMO (40%), HOMO->L+1 (49%)
	S_5	261.01	0.0027	H-1->L+3 (12%), HOMO->L+3 (80%)
	S_6	259.57	0.2571	HOMO->L+2 (71%)
	S_7	255.50	0.4544	H-3->LUMO (47%), H-1->L+1 (10%), HOMO->L+1 (35%)
	S_8	248.65	0.0339	H-2->L+3 (63%), H-1->L+1 (23%)
	S_9	246.58	0.1663	H-2->L+3 (24%), H-1->L+1 (50%), HOMO->L+2 (12%)
	S_{11}	241.10	0.0502	H-1->L+2 (82%)

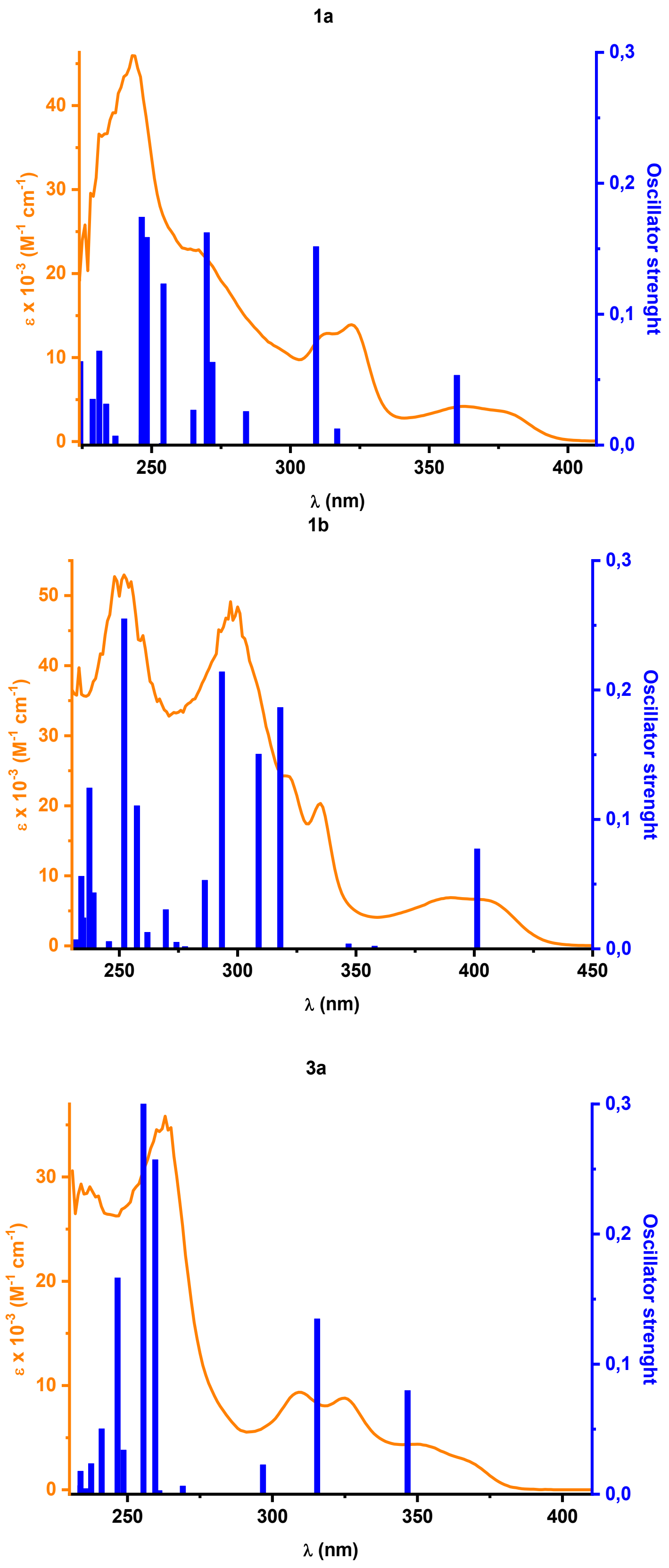

Figure S16. Calculated stick absorption spectra for **1a**, **1b** and **3a** in the ground state in CH_2Cl_2 compared with the experimental data.

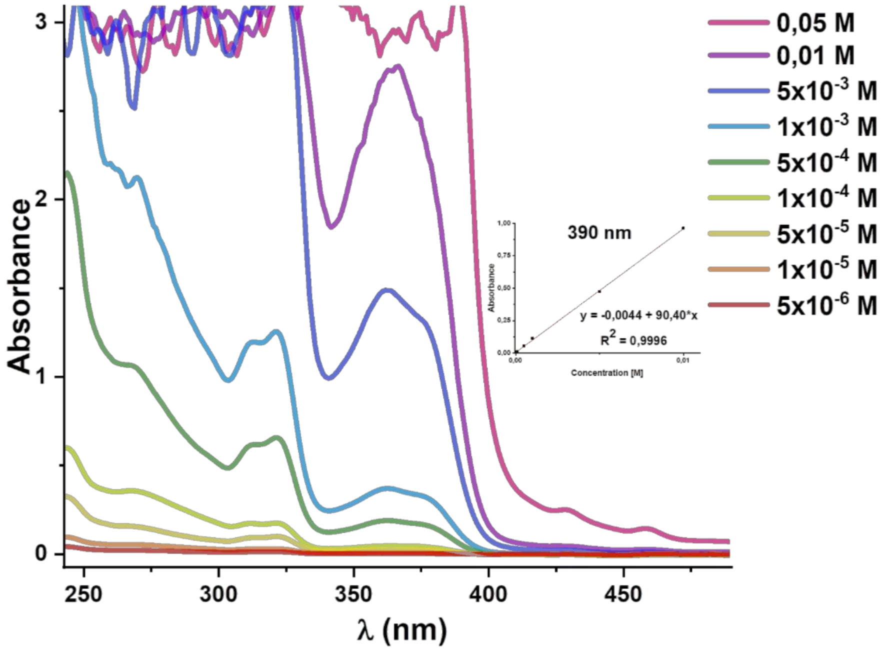

Figure S17. Low-energy region of the UV−vis absorption spectra of **1a** in CH_2Cl_2 at different concentrations. c) Representation of the absorbance at the 390 nm band *vs* concentration.

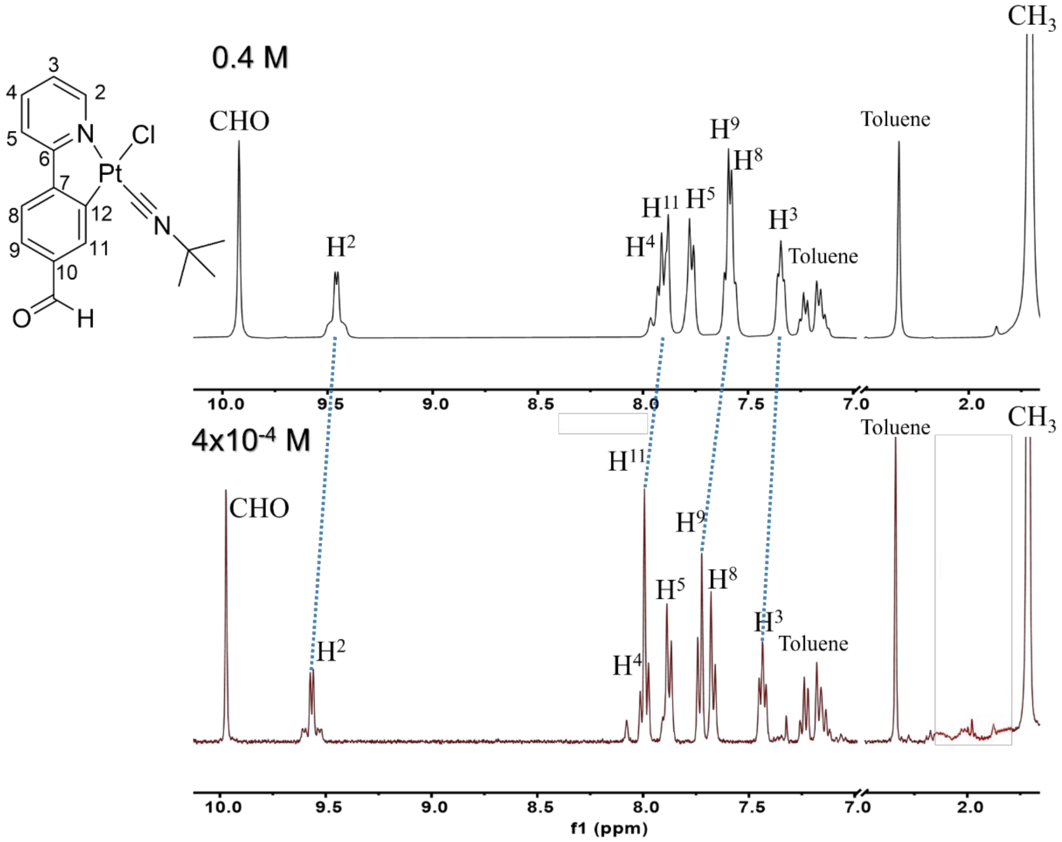

Figure S18. ^{1}H NMR spectra of **1b·0.5Toluene** in CD_2Cl_2 at different concentrations.

Table S10. Photophysical data for compounds **1a**, **1b**, **3a** and **3b** in solution and in polystyrene film (PS).

Compound	Media	[M]	T[a]/K	λ_{em}/nm (λ_{ex}/nm)	τ/μs	ϕ
**1a**	CH_2Cl_2	5x10^{-5}	77	465[a], 552_{max}, 645 (365)<br>558, 645_{max} (440)	14.9 (465)<br>9.7 (552)<br>6.4 (645)	
	THF	5x10^{-5}	77	460[a], 558 (365)<br>460[a], 564_{max} (420)	26.9 (460)<br>6.5 (564)	
	PS	(10% *wt*)	298	467[a], 580_{max} (385)<br>585 (410)<br>635 (480)	0.2 (76%), 2.6 (24%) (467)<br>0.2 (59%), 1.3 (41%) (580)<br>0.2 (54%), 1.6 (46%) (635)	0.06 (385)<br>0.19 (480)
	PS	(1% *wt*)	298	467[a], 570 (385)<br>575 (420)	0.2 (65%), 2.1 (35%) (467)<br>0.2 (69%), 1.8 (31%) (575)	0.05
**1b**	CH_2Cl_2	5x10^{-4}	298	529[a] (410)	1.6[b]	0.03
		5x10^{-5}	77	540[a], 640 (440)	14.8 (540)<br>10.1 (640)	
	THF	5x10^{-4}	298	529[a] (415)	<1%[b]	0.02
		5x10^{-5}	77	517[a] (415)	26.0[b]	
	PS	(10% *wt*)	298	536[a]	0.4 (24%), 6.6 (76%)[b]	0.25
	PS	(1% *wt*)	298	524[a]	1.0[b]	0.27
**3a**	CH_2Cl_2	5x10^{-4}	298	471[a] (365)	0.1 (24%), 0.7 (76%)[b]	0.01
		5x10^{-5}	77	475[a], 577 (365)<br>594 (420)	39.1 (475)<br>6.6 (580)	
	PS	(1% *wt*)	298	474_{sh}, 600_{max} (365)	<1% (474)<br>1.8 (61%), 3.0 (39%) (600)	0.27
**3b**	CH_2Cl_2	5x10^{-4}	298	511[a] (375)	0.3 (12%), 3.1 (88%)[b]	0.02
		5x10^{-5}	77	524[a] (385)<br>524[a], 618 (420)	24.5 (524)<br>14.5 (618)	
	PS	(1% *wt*)	298	524[a]	3.2[b]	0.09

[a]) the $\nu_{0\rightarrow 0}$ transition of the structured emission, [b]) Measured in the highest energy peak

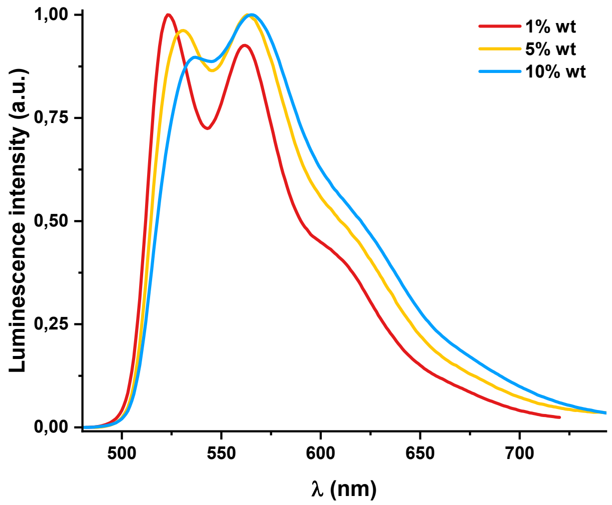

Figure S19. Normalized excitation (dashed line) and emission (solid line) spectra in PS at 1-10% *wt* at 298 K of **1b** (λ_{ex} 420 nm).

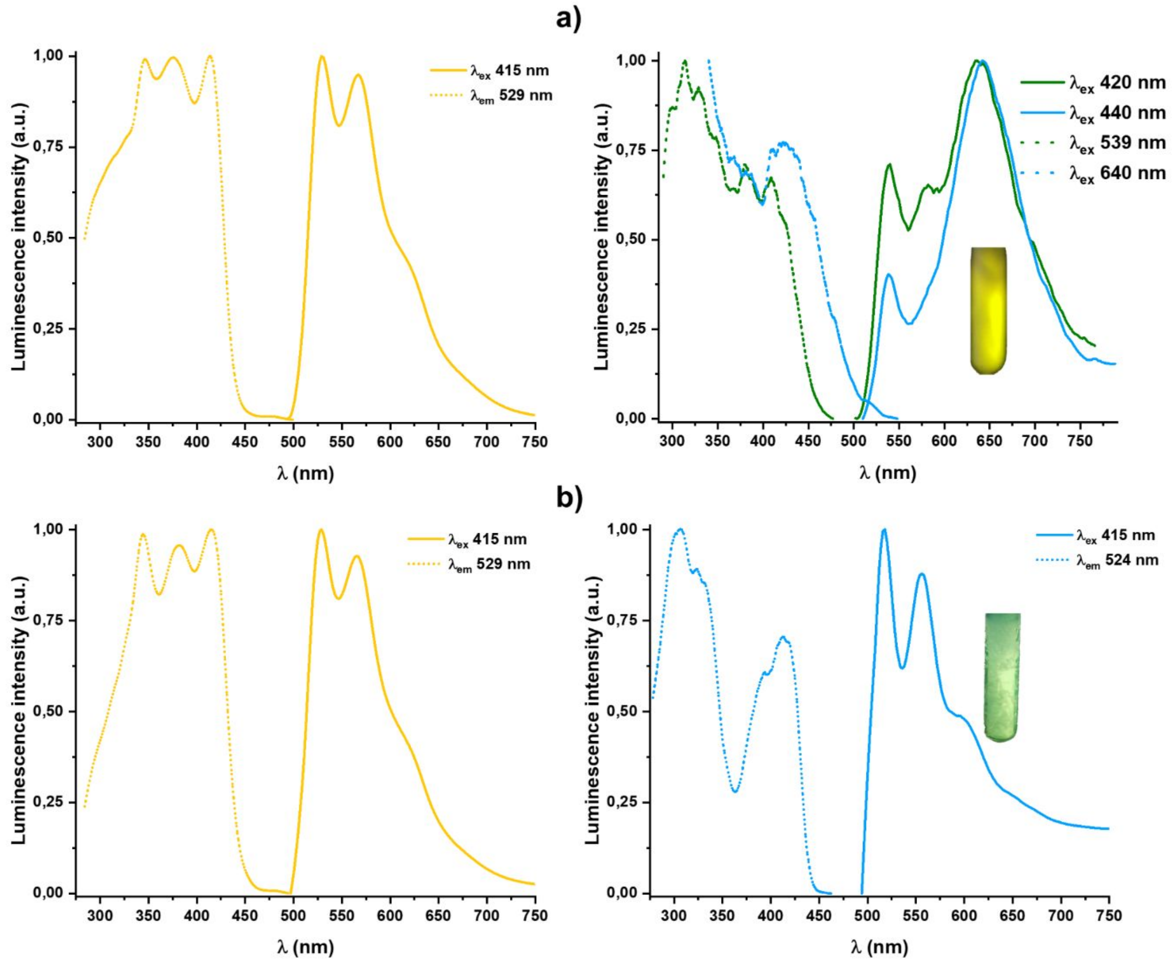

Figure S20. Normalized excitation (dashed line) and emission (solid line) spectra of **1b** in CH_2Cl_2 (a) and THF (b) at 5×10^{-4} M at 298 K (left) and at 5×10^{-5} M at 77 K (right).

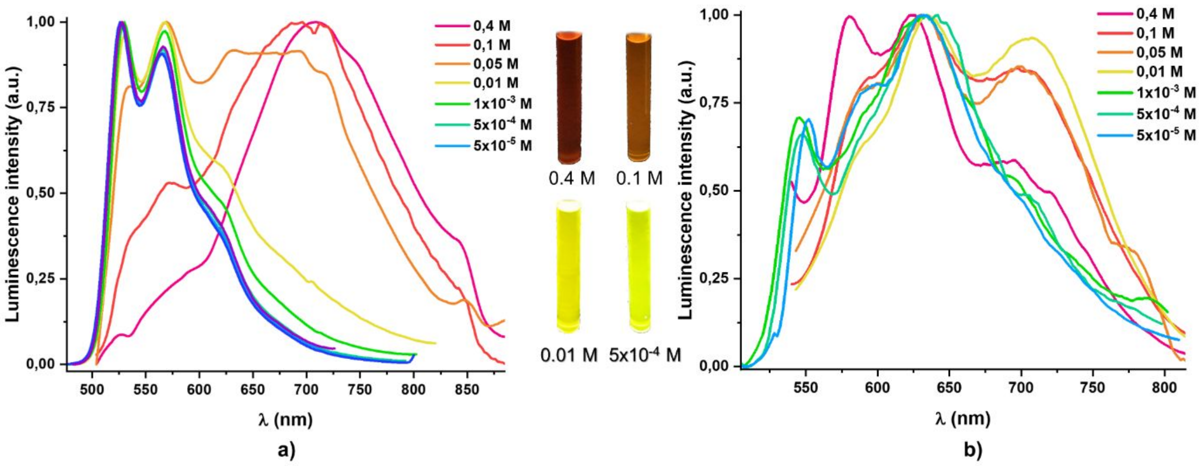

Figure S21. Emission spectra of **1b** in CH_2Cl_2 at different concentrations at a) 298 K (5 x 10^{-5} to 0.01 M, λ_{ex} 420 nm; 0.01-0.4 M, λ_{ex} 480 nm) and b) 77 K (5 x 10^{-5} to 10^{-3} M, λ_{ex} 420 nm; 0.01-0.4 M, λ_{ex} 520 nm). Images under UV light (365 nm).

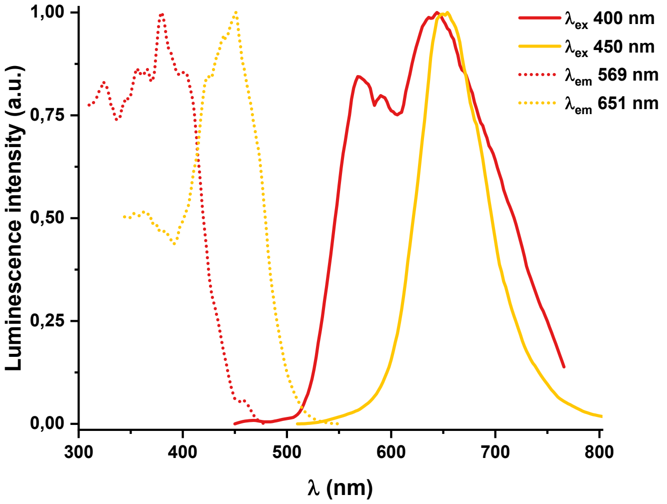

Figure S22. Normalized excitation and emission spectra of **1a** in CH_2Cl_2 5 x 10^{-4} M at 77 K.

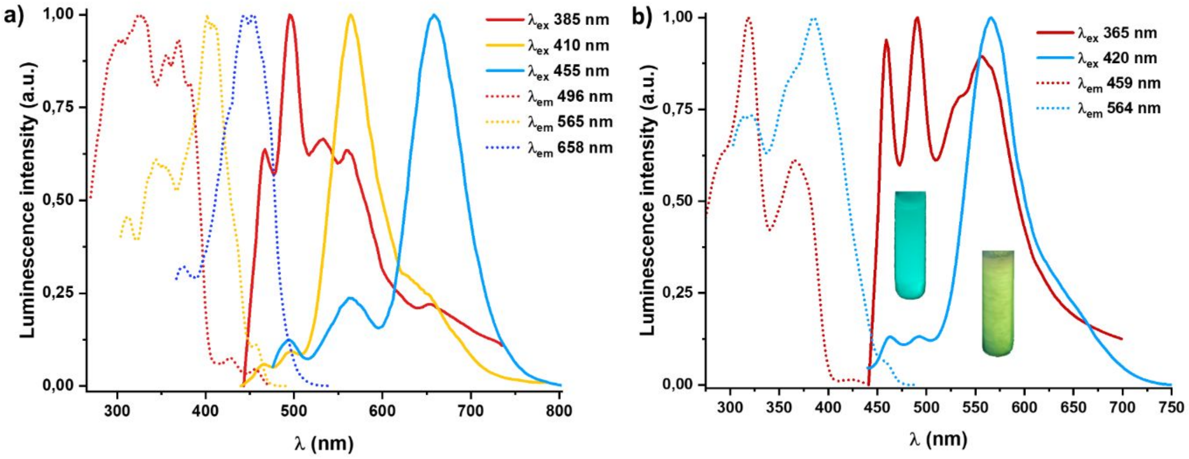

Figure S23. Normalized excitation and emission spectra of **1a** in THF 5 x 10^{-4} M (a), 5 x 10^{-5} M (b) at 77 K. Photographs taken under lamps of the ~λ_{exc} indicated.

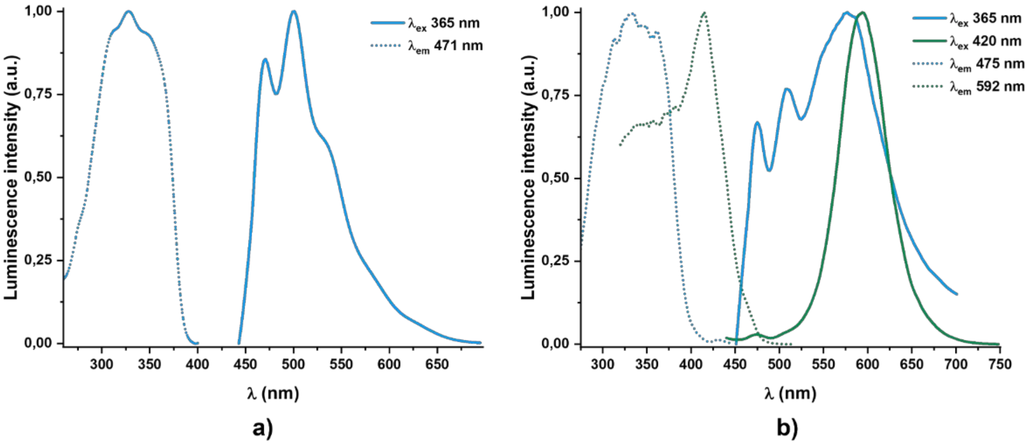

Figure S24. Normalized emission spectra of **3a** in CH_2Cl_2 at 5×10^{-4} M at 298 K (a) and 77 K (b).

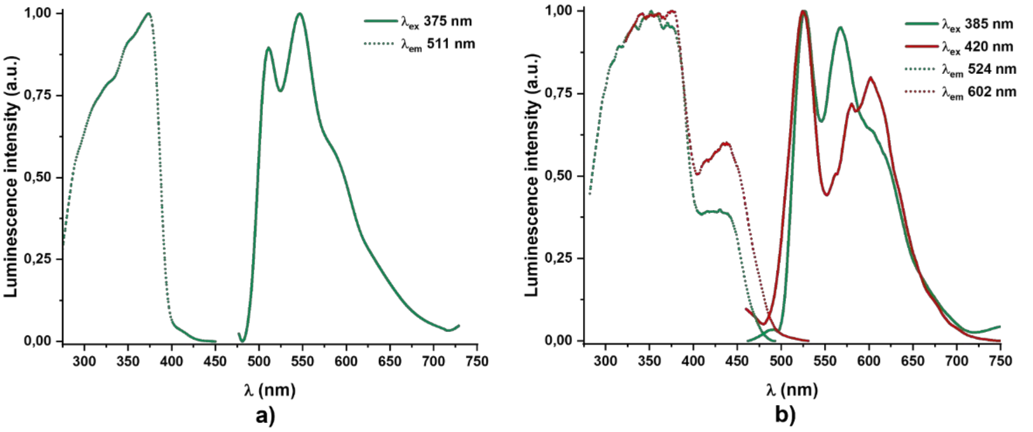

Figure S25. Normalized emission spectra of **3b** in CH_2Cl_2 at 5×10^{-4} M at 298 K (a) and 77 K (b).

Table S11. Composition (%) of Frontier MOs in terms of ligands and metals in the first triplet state for **1a**, **1b** and **3a** in CH_2Cl_2.

1a

MO	eV	Pt	dfppy	CNBu^t	Cl
SOMO	-3.61	6	91	2	1
SOMO-1	-4.64	9	90	0	1

1b

MO	eV	Pt	ppy-CHO	CNBu^t	Cl
SOMO	-4	4	95	1	0
SOMO-1	-4.7	16	81	0	3

3a

MO	eV	Pt	dfppy	CNBu^t
SOMO	-4.15	7	88	5
SOMO-1	-5.13	3	96	1

	**1a**	**1b**	**3a**
Spin density on Pt	0.137727	0.191229	0.084966
E of emission (T_1-S_0 opt)	521 nm	619 nm	531 nm

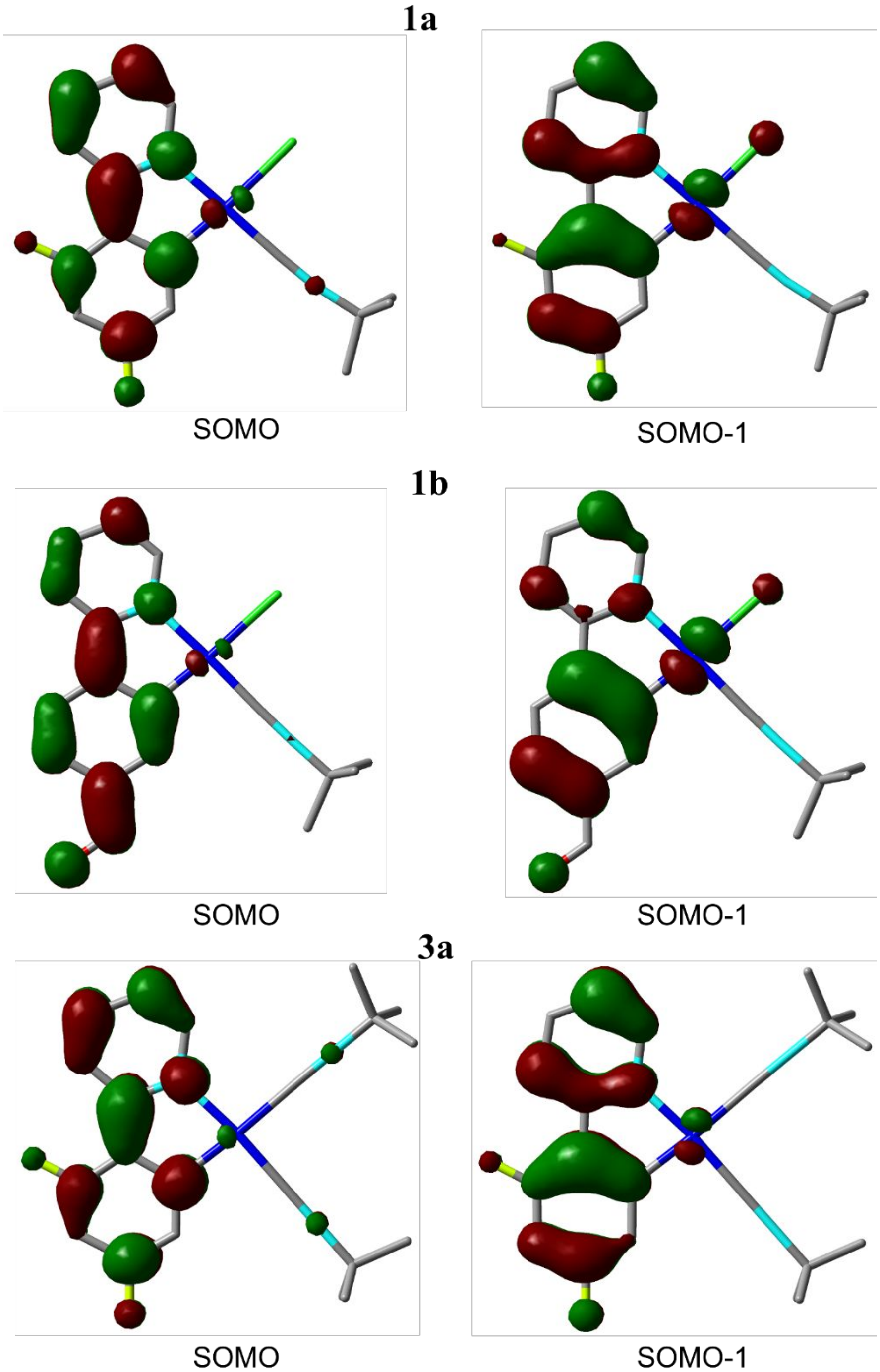

Figure S26. Frontier orbitals plots obtained by DFT for the first triplet state of **1a**, **1b** and **3a**.

Table S12. Absorption data for **1a**, **1b**, **3a** and **3b** in the solid state.

Compound	λ_{abs}/nm
**1a**	238, 258, 320, 388, 425, 472, tail to 550
**1a-$CHCl_3$-R**	288, 317, 370, 508, 545, tail to 600
**1a-$CHCl_3$-G**	320, 370, 390, 435, 465, tail to 500
**1a-$CHCl_3$-R with vapors of acetone**	288, 300, 318, 375, 410, 450, tail to 520
**1a-$CHCl_3$-G-ground**	296, 318, 372, 393, 445, tail to 550
**1a-$CHCl_3$-G-Δ**	298, 320, 372, 393, 470, tail to 550
**1a(THF)**	280, 315, 376, 449, tail to 520
**1a(MeOH)**	280, 315, 375, 493, tail to 610
**1a(EtOH)**	282, 319, 371, 505, tail to 610
**1a($CHCl_3$)**	320, 385, 461, 487, 510, 530, 550, tail to 630
**1a(CH_2Cl_2)**	235, 270, 318, 368, 519, 560, tail to 650
**1b**	297, 335, 356, 398, 420, 493_{sh}, 530_{sh}, tail to 550
**1b·ground**	297, 322, 342, 401, 427, 500_{sh}, 530_{sh}, tail to 550
**1b·0.5Toluene**	297, 334, 373, 425, 509, 550, tail to 610
**1b-0.5Toluene after 1 month**	297, 334, 395, 425, 550, tail to 610
**1b-0.5Toluene with vapors of acetone**	297, 332, 357, 401, 422, 494_{sh}, 532_{sh}, tail to 550
**2a**	293, 321, 381, 425, 516, tail to 620
**2b**	293, 311, 333, 398, 420, tail to 500
**3a**	317, 353, 370, 404_{sh}, 462_{sh}, tail to 480
**3a·0.25CH_2Cl_2**	236, 328, 351, 373, 436_{sh}, 476_{sh}, tail to 485
**3a-Acetone**	302, 319, 355, 370, 432_{sh}, 525, tail to 600
**3a-$CHCl_3$**	279, 317, 334, 352, 370, 465_{sh}, 530, tail to 610
**3a-THF**	277, 317, 349, 370, 432_{sh}, 462_{sh}, 525, tail to 600
**3b**	283, 299, 315, 340, 358, 380, 440_{sh}, tail to 480

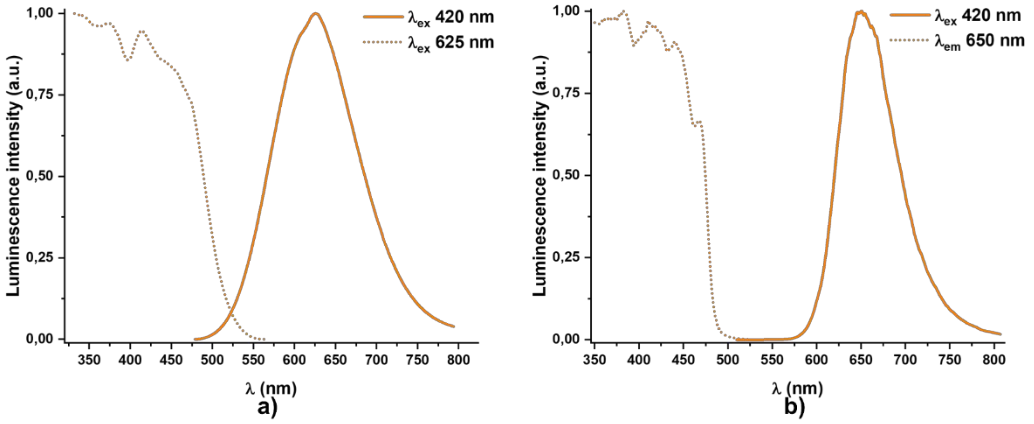

Figure S27. Normalized excitation and emission spectra of **1a** in solid state at 298 K (a) and 77 K (b).

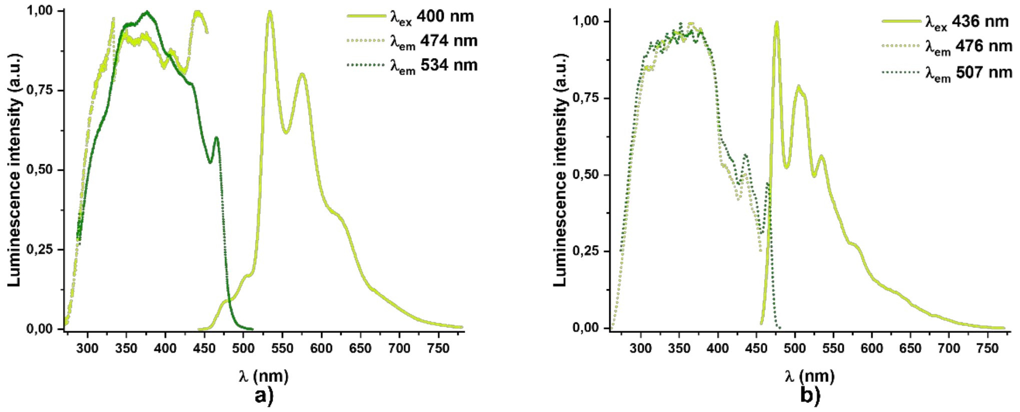

Figure S28. Normalized excitation and emission spectra of **1a-CHCl$_3$-G** (identical to the emission of crystals of **1a·CHCl$_3$**) in solid state at 298 K (a) and 77 K (b).

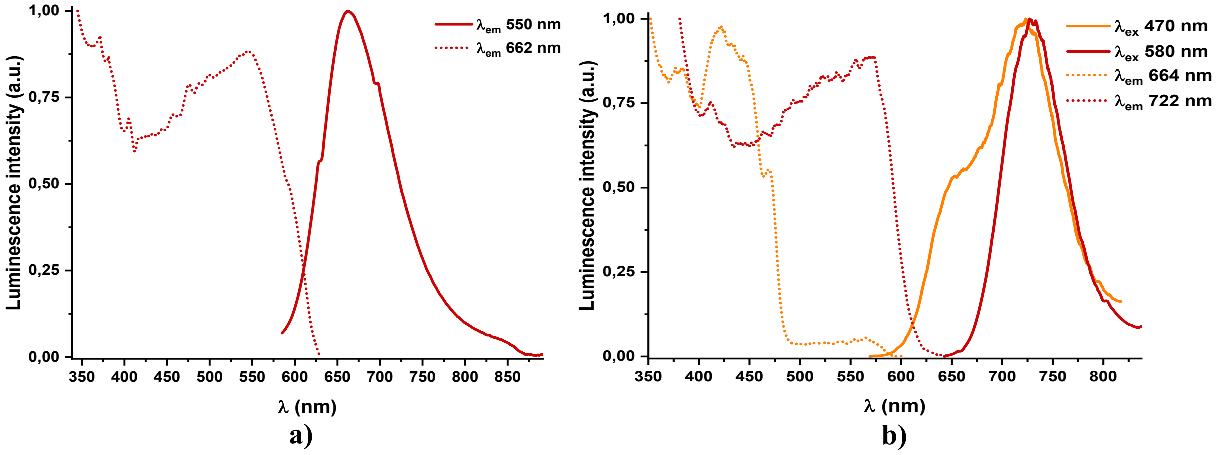

Figure S29. Normalized excitation and emission spectra of **1a-CHCl$_3$-R** in solid state at 298 K (a) and 77 K (b).

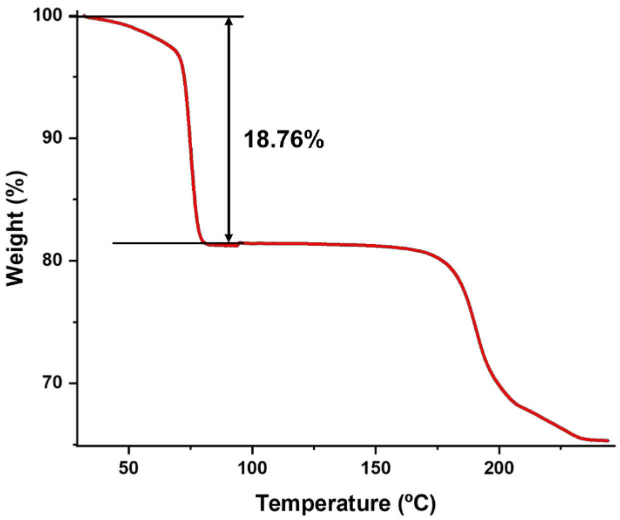

a)

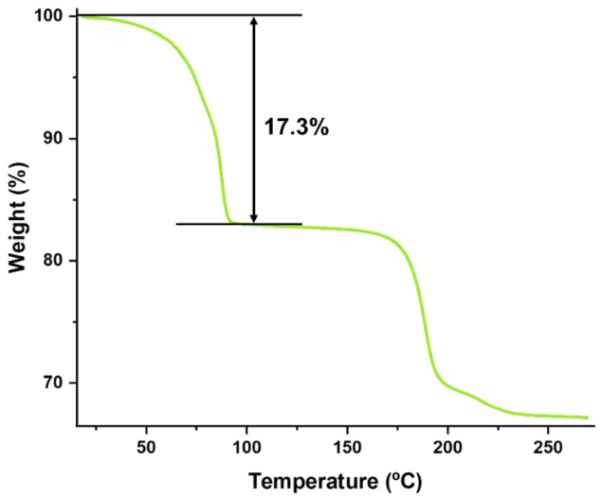

b)

Figure S30. Thermogravimetric analysis (scan rate of 5 °C min^{-1}) of a) (**1a-CHCl$_3$-R** form), and b) (**1a-CHCl$_3$-G** form), revealing their composition as 1a·CHCl$_3$.

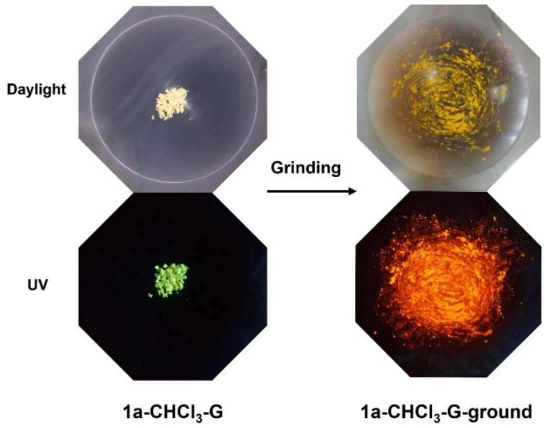

Figure S31. Photographs showing the color and the luminescence change of **1a-CHCl$_3$-G** after grinding.

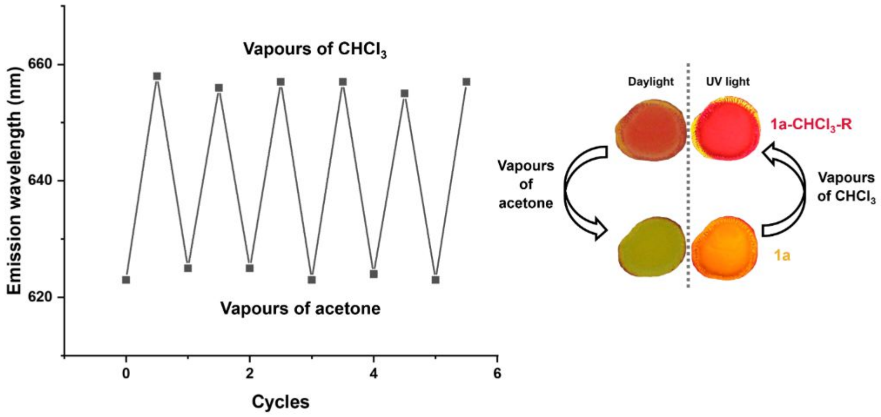

Figure S32. Plot of emission wavelength changes of **1a** during 5-cycles of being exposed to $CHCl_3$ and acetone vapors. The procedure is accomplished by depositing a drop of **1a** in acetone on a glass and letting it to dry, once the solid is dried (orange emission) its emission is measured from the glass holder. Then, the solid is exposed to the $CHCl_3$ vapor, producing a red-shift in the color of the solid and its emission. After measuring the new emission spectrum, the solid reverts to the initial color with acetone vapors. This procedure is carried out for five cycles without noticing an apparent change in the sample.

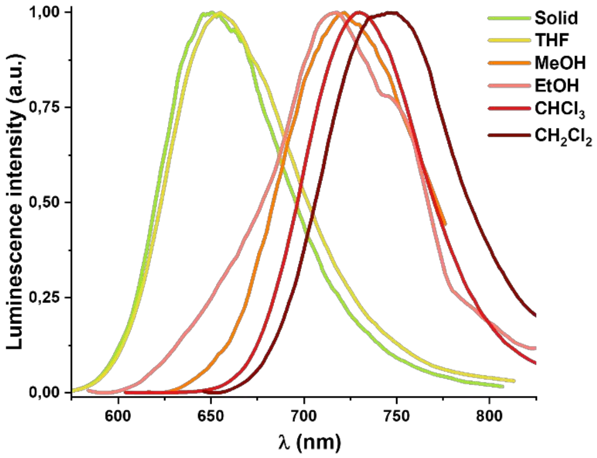

Figure S33. Normalized emission spectra of **1a** solid and those solids obtained by evaporation of the appropriate solutions of **1a** in different solvents at 77 K. (**1a** Solid and THF λ_{ex} 420 nm; MeOH and EtOH λ_{ex} 530 nm; $CHCl_3$ and CH_2Cl_2 λ_{ex} 570 nm).

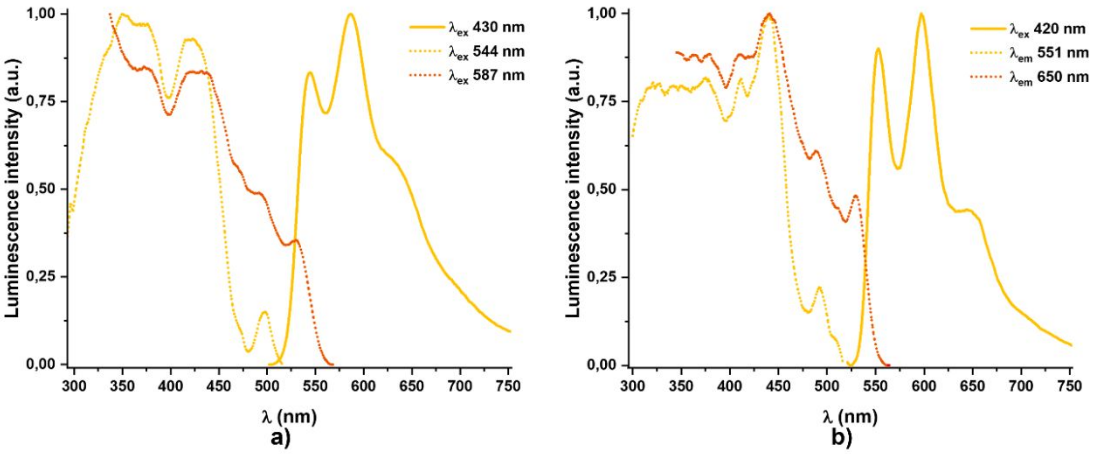

Figure S34. Normalized excitation and emission spectra of **1b** in solid state at 298 K (a) and 77 K (b).

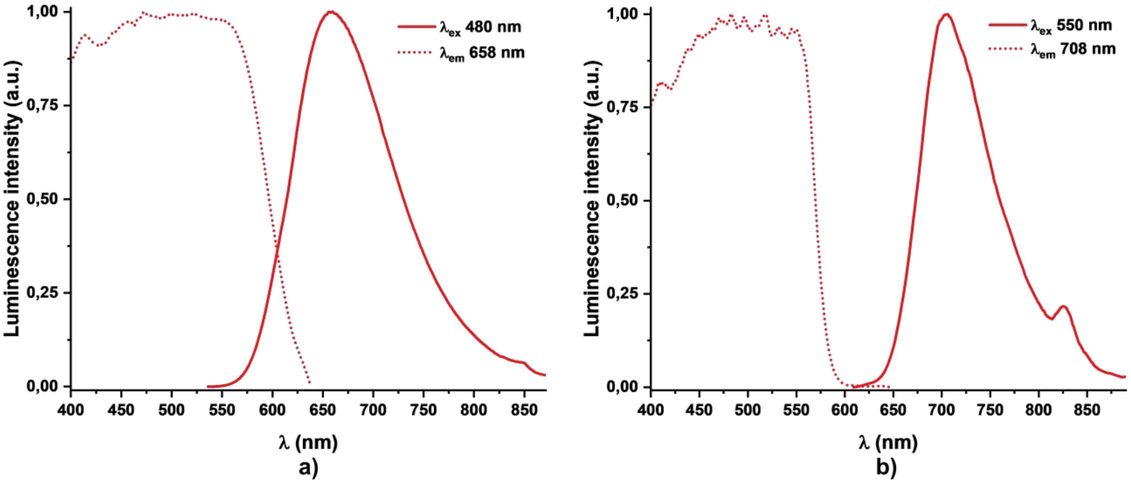

Figure S35. Normalized excitation and emission spectra of **1b·0.5Toluene** in solid state at 298 K (a) and 77 K (b).

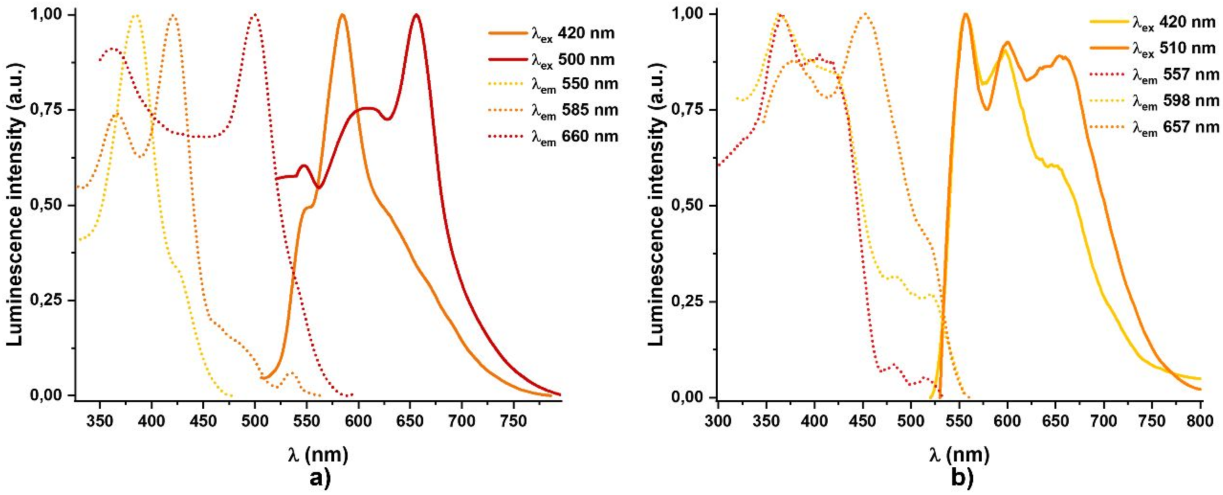

Figure S36. Normalized excitation and emission spectra of **1b-ground** in solid state at 298 K (a) and 77 K (b).

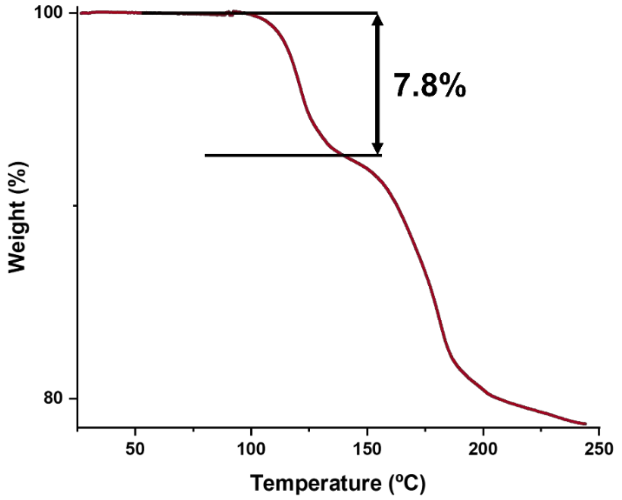

Figure S37. Thermogravimetric analysis (scan rate of 5 °C min^{-1}) of the pristine solid **1b** after exposure to saturated vapors of toluene, revealing its composition as **1b·0.5Toluene**.

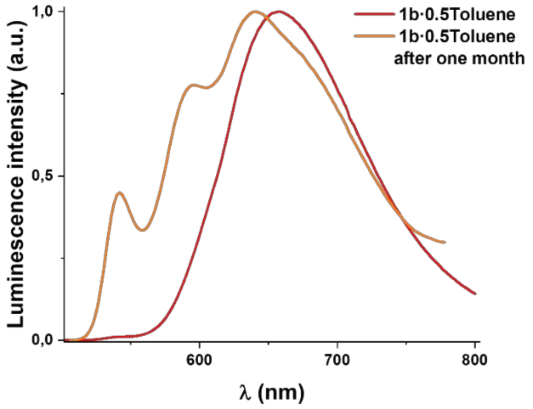

Figure S38. Normalized emission and excitation spectra of **1b·0.5Toluene** after one month exposed to air at 298 K.

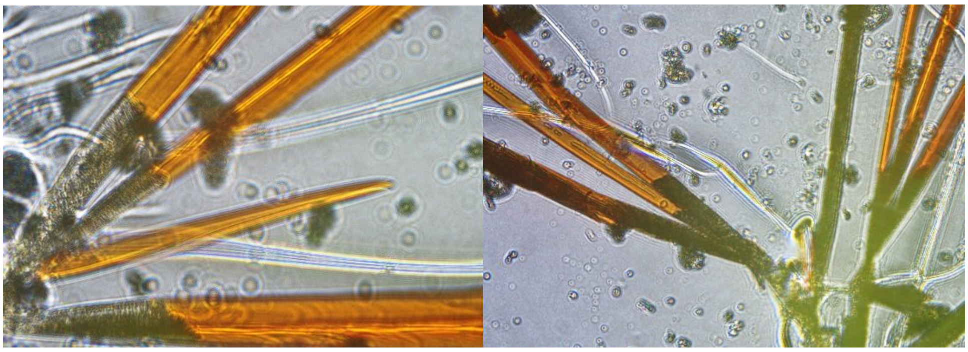

Figure S39. Images of the red crystals in the PS film (10%) of **1b**, obtained by exposition to toluene, after two months on standing at air. The left image has a magnification of 40x, while right image has a magnification of 10x.

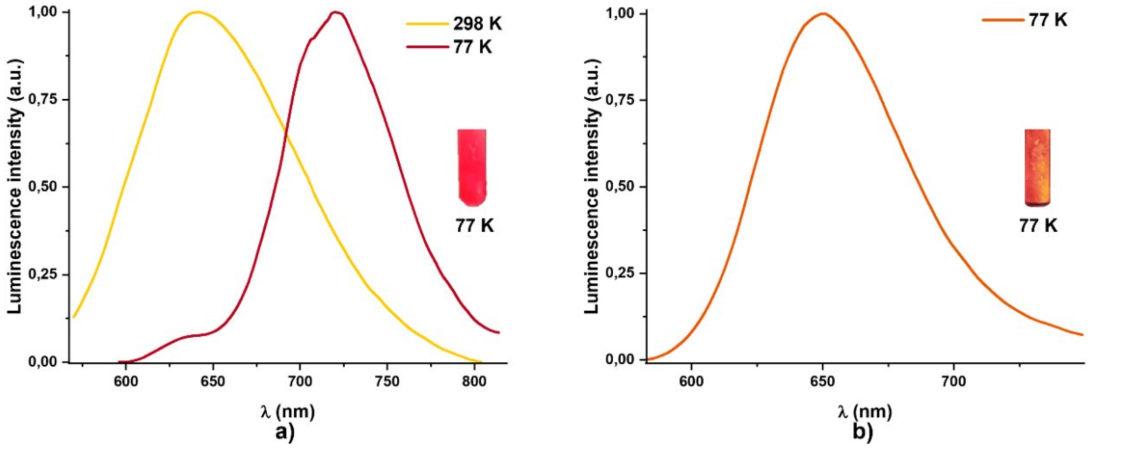

Figure S40. Normalized emission spectra of **2a** (λ_{ex} 550 nm 298 K, λ_{ex} 580 nm 77 K) (a) and **2b** (λ_{ex} 515 nm 77 K) (b) in solid state.

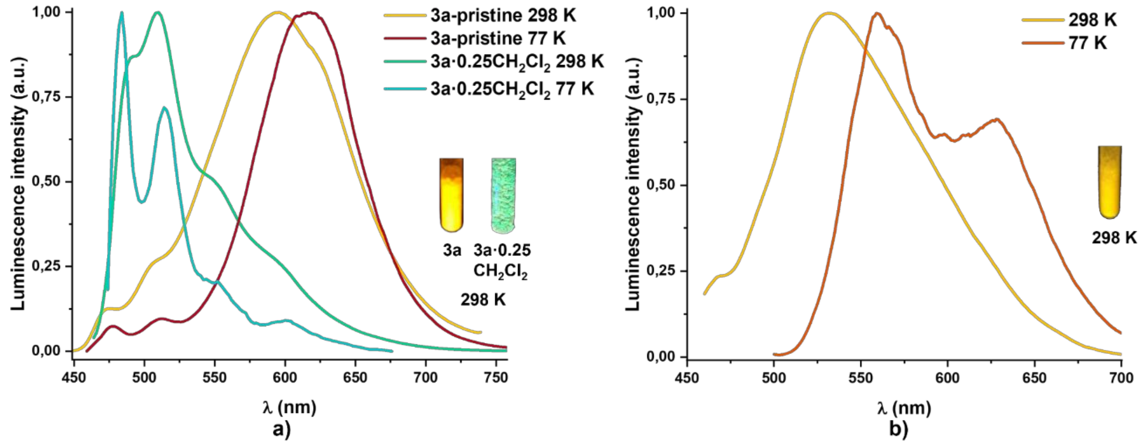

Figure S41. Emission spectra in solid state at 298 K and 77 K of **3a-pristine** and **3a·0.25CH$_2$Cl$_2$** (a) and **3b-pristine** (b).

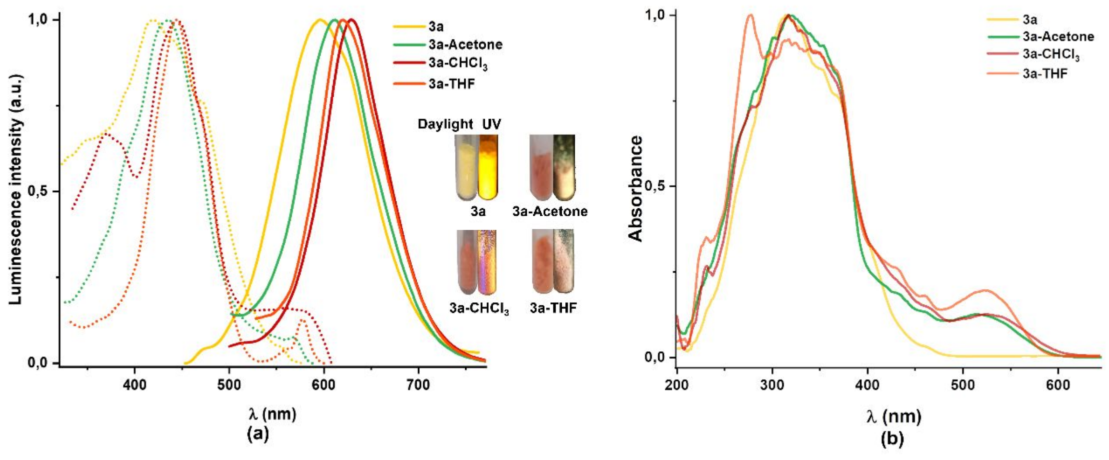

Figure S42. a) Normalized excitation and emission spectra of vapochromic response of **3a-pristine** in solid state at 298 K (λ_{ex} 420-430 nm). b) Normalized absorption spectra calculated from their reflectance spectra in the solid state.

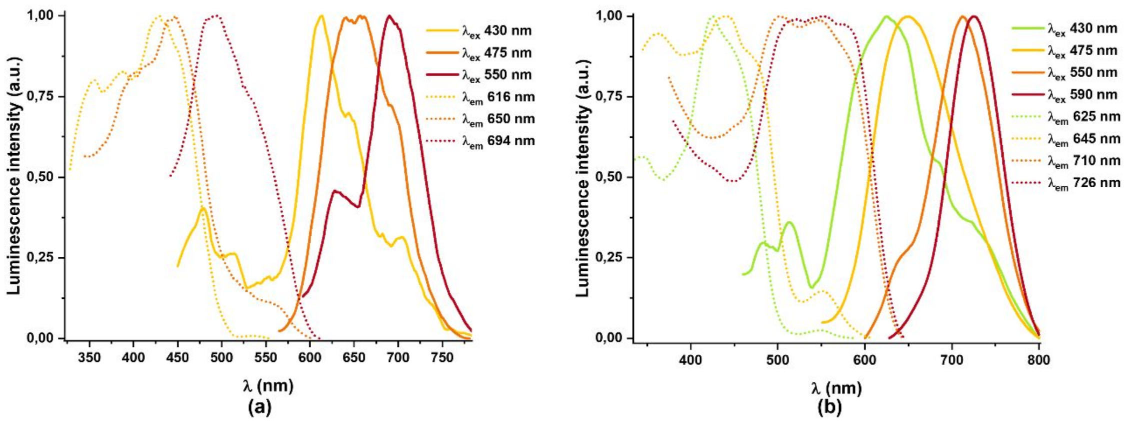

Figure S43. a) Normalized emission and excitation spectra of **3a-acetone** (a) and **3a-CHCl$_3$** (b) in solid state at 77 K.

Table S13. Composition (%) of Frontier MOs in terms of ligands and metals in the ground state for **1a, [1a]$_2$, [1a]$_3$** and **1b, [1b]$_4$** in gas phase.

1a

MO	eV	Pt	dfppy	CNBu^t	Cl
LUMO+3	-0.28	44	30	15	10
LUMO+2	-0.37	23	54	22	1
LUMO+1	-1.04	1	98	1	0
LUMO	-1.88	7	87	5	1
HOMO	-5.82	30	32	0	37
HOMO-1	-6.34	13	3	1	83
HOMO-2	-6.39	2	88	0	10
HOMO-3	-6.63	91	7	1	1

[1a]$_2$

MO	eV	Pt	dfppy	CNBu^t	Cl
LUMO+3	-0,91	1	98	1	0
LUMO+2	-1,15	1	97	1	0
LUMO+1	-1,57	3	94	2	0
LUMO	-2,02	9	84	6	1
HOMO	-5,7	84	6	2	7
HOMO-1	-5,74	30	47	1	22
HOMO-2	-5,97	37	37	1	26
HOMO-3	-6,27	5	77	0	18

[1a]$_3$

MO	eV	Pt	dfppy	CNBu^t	Cl
LUMO+3	-1,15	2	97	2	0
LUMO+2	-1,39	3	96	2	0
LUMO+1	-1,72	5	91	3	0
LUMO	-2,04	11	82	7	1
HOMO	-5,22	86	8	3	3
HOMO-1	-5,6	28	53	1	19
HOMO-2	-5,78	33	38	1	28
HOMO-3	-5,94	37	33	1	30

1b

MO	eV	Pt	ppy-CHO	CNBu^t	Cl
LUMO+3	-0.40	25	47	27	1
LUMO+2	-0.66	1	98	1	0
LUMO+1	-1.46	3	94	4	0
LUMO	-2.35	4	93	2	0
HOMO	-5.83	31	32	0	37
HOMO-1	-6.38	14	4	1	82
HOMO-2	-6.64	77	19	1	3
HOMO-3	-6.66	3	71	0	26

[1b]$_4$

MO	eV	Pt	ppy-CHO	CNBu^t	Cl
LUMO+3	-2.09	1	98	0	0
LUMO+2	-2.15	2	97	1	0
LUMO+1	-2.29	5	92	2	0
LUMO	-2.46	8	88	4	1
HOMO	-5.43	81	14	3	2
HOMO-1	-5.77	33	54	1	12
HOMO-2	-5.83	37	39	1	24
HOMO-3	-5.83	34	38	1	27

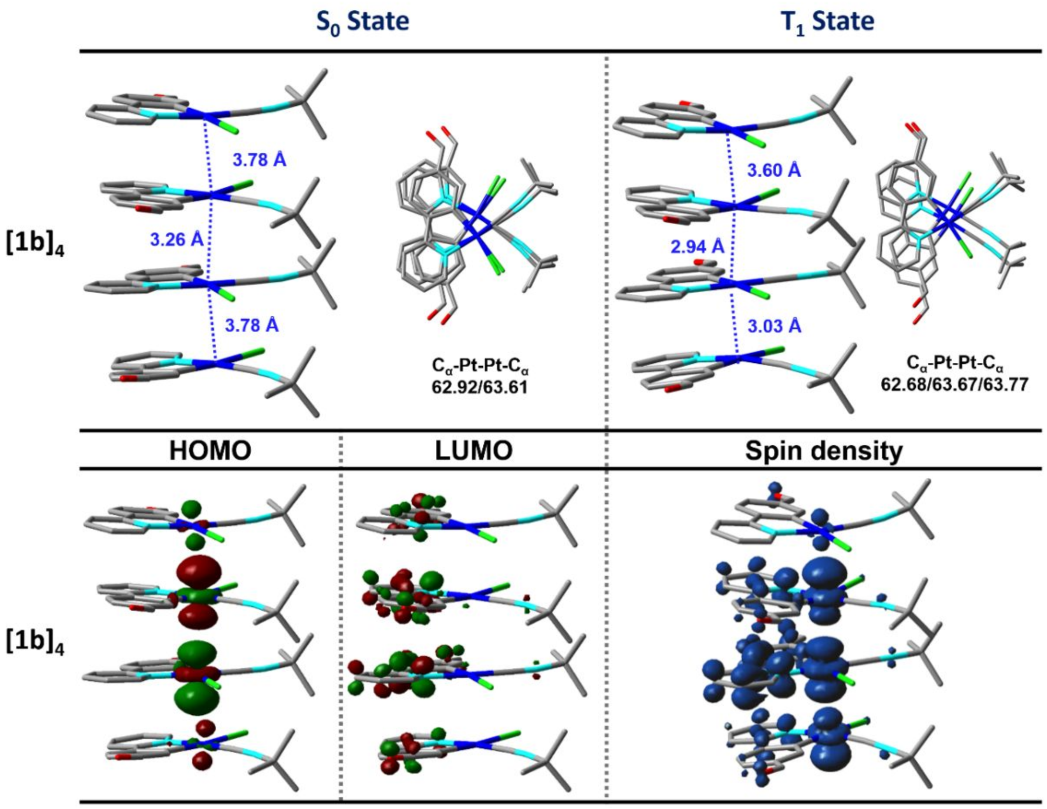

Figure S44. Optimized geometries of **[1b]$_4$** model at the S_0 and T_1 states. Contour plots of HOMO and LUMO at the S_0 and spin density at the T_1 optimized geometries [B3LYP/6-31G(d,p)].

References

(1) Cho, J.-Y.; Suponitsky, K. Y.; Li, J.; Timofeeva, T. V.; Barlow, S.; Marder, S. R. Cyclometalated platinum complexes: High-yield synthesis, characterization, and a crystal structure. *J. Organomet. Chem.* **2005**, *690*, 4090-4093.

(2) Karakus, C.; Fischer, L. H.; Schmeding, S.; Hummel, J.; Risch, N.; Schaferling, M.; Holder, E. Oxygen and temperature sensitivity of blue to green to yellow light-emitting Pt(II) complexes. *Dalton Trans.* **2012**, *41*, 9623-9632.

(3) Micutz, M.; Iliş, M.; Staicu, T.; Dumitraşcu, F.; Pasuk, I.; Molard, Y.; Roisnel, T.; Cîrcu, V. Luminescent liquid crystalline materials based on palladium(II) imine derivatives containing the 2-phenylpyridine core. *Dalton Trans.* **2014**, *43*, 1151-1161.

(4) Kvam, P.-I.; Songstad, J. Preparation and Characterization of Some Cyclometalated Pt(II) Complexes from 2-Phenylpyridine and 2-(2'-Thienyl)pyridine. *Acta Chem. Scan.* **1995**, *49*, 313-324.

(5) Farrugia, L. J. WinGX suite for small-molecule single-crystal crystallography. *Appl. Crystallogr.* **1999**, *32*, 837-838.

(6) Sheldrick, G. M. SHELXT – Integrated space-group and crystal structure determination. *Acta Crystallogr., Sect. A: Found. Crystallogr.* **2015**, *71*, 3-8.

(7) Sheldrick, G. M. Crystal structure refinement with SHELXL. *Acta Crystallogr C Struct Chem* **2015**, *71*, 3-8.

(8) M. J. Frisch, G. W. Truks, H. B. Schlegel, G. E. Scuseria, M. A. Robb, J. R. Cheeseman, G. Scalmani, V. Barone, G. A. Petersson, H. Nakatsuji, X. Li, M. Caricato, A. V. Marenich, J. Bloino, B. G. Janesko, R. Gomperts, B. Mennucci, H. P. Hratchian, J. V. Ortiz, A. F. Izmaylov, J. L. Sonnenberg, D. Williams-Young, F. Ding, F. Lipparini, F. Egidi, J. Goings, B. Peng, A. Petrone, T. Henderson, D. Ranasinghe, V. G. Zakrzewski, J. Gao, N. Rega, G. Zheng, W. Liang, M. Hada, M. Ehara, K. Toyota, R. Fukuda, J. Hasegawa, M. Ishida, T. Nakajima, Y. Honda, O. Kitao, H. Nakai, T. Vreven, K. Throssell, J. A. Montgomery, Jr., J. E. Peralta, F. Ogliaro, M. J. Bearpark, J. J. Heyd, E. N. Brothers, K. N. Kudin, V. N. Staroverov, T. A. Keith, R. Kobayashi, J. Normand, K. Raghavachari, A. P. Rendell, J. C. Burant, S. S. Iyengar, J. Tomasi, M. Cossi, J. M. Millam, M. Klene, C. Adamo, R. Cammi, J. W. Ochterski, R. L. Martin, K. Morokuma, O. Farkas, J. B. Foresman, and D. J. Fox, Gaussian 16, Revision A.03, Inc., Wallingford CT, 2016.

(9) (a) Becke, A. D. Density-functional thermochemistry. III. The role of exact exchange. *J. Chem. Phys.* **1993**, *98*, 5648-5652. (b) Becke, A. D. Density-functional exchange-energy approximation with correct asymptotic behavior. *Phys. Rev. A* **1988**, *38*, 3098-3100.

(10) Wadt, W. R.; Hay, P. J. Ab initio effective core potentials for molecular calculations. Potentials for main group elements Na to Bi. *J. Chem. Phys.* **1985**, *82*, 284-298.

(11) Barone, V.; Cossi, M. Quantum Calculation of Molecular Energies and Energy Gradients in Solution by a Conductor Solvent Model. *J. Phys. Chem. A* **1998**, *102*, 1995-2001.

(12) (a) Grimme, S.; Antony, J.; Ehrlich, S.; Krieg, H. A consistent and accurate ab initio parametrization of density functional dispersion correction (DFT-D) for the 94 elements H-Pu. *J. Chem. Phys.* **2010**, *132*, 154104. (b) Grimme, S.; Ehrlich, S.; Goerigk, L. Effect of the damping function in dispersion corrected density functional theory. *J. Comput. Chem.* **2011**, *32*, 1456-1465.

(13) O'Boyle, N. M.; Tenderholt, A. L.; Langner, K. M. cclib: A library for package-independent computational chemistry algorithms. *J. Comput. Chem.* **2008**, *29*, 839-845.

(14) Speck, A. L. Single-crystal structure validation with the program PLATON. *J. Appl. Cryst.* **2003**, *36*, 7-13.
